# *ZIC1* is a context-dependent medulloblastoma driver in the rhombic lip

**DOI:** 10.1038/s41588-024-02014-z

**Published:** 2025-01-03

**Authors:** John J. Y. Lee, Ran Tao, Zhen You, Parthiv Haldipur, Anders W. Erickson, Hamza Farooq, Liam D. Hendriske, Namal Abeysundara, Cory M. Richman, Evan Y. Wang, Neha Das Gupta, Jennifer Hadley, Melissa Batts, Christopher W. Mount, Xiaochong Wu, Alex Rasnitsyn, Swneke Bailey, Florence M. G. Cavalli, Sorana Morrissy, Livia Garzia, Kulandaimanuvel Antony Michealraj, Abhi Visvanathan, Vernon Fong, Jonelle Palotta, Raul Suarez, Bryn G. Livingston, Miao Liu, Betty Luu, Craig Daniels, James Loukides, Anne Bendel, Pim J. French, Johan M. Kros, Andrey Korshunov, Marcel Kool, Fernando Chico Ponce de León, Mario Perezpeña-Diazconti, Boleslaw Lach, Sheila K. Singh, Sarah E. S. Leary, Byung-Kyu Cho, Seung-Ki Kim, Kyu-Chang Wang, Ji-Yeoun Lee, Teiji Tominaga, William A. Weiss, Joanna J. Phillips, Shizhong Dai, Gelareh Zadeh, Ali G. Saad, László Bognár, Almos Klekner, Ian F. Pollack, Ronald L. Hamilton, Young-shin Ra, Wieslawa A. Grajkowska, Marta Perek-Polnik, Reid C. Thompson, Anna M. Kenney, Michael K. Cooper, Stephen C. Mack, Nada Jabado, Mathieu Lupien, Marco Gallo, Vijay Ramaswamy, Mario L. Suva, Hiromichi Suzuki, Kathleen J. Millen, L. Frank Huang, Paul A. Northcott, Michael D. Taylor

**Affiliations:** 1 Department of Laboratory Medicine and Pathobiology, University of Toronto, Toronto, Ontario Canada; 2 The Arthur and Sonia Labatt Brain Tumor Research Center, The Hospital for Sick Children, Toronto, Ontario Canada; 3 Developmental and Stem Cell Biology Program, The Hospital for Sick Children, Toronto, Ontario Canada; 4 Department of Pathology and Krantz Family Center for Cancer Research, Massachusetts General Hospital and Harvard Medical School, Boston, MA USA; 5 Broad Institute of Harvard and Massachusetts Institute of Technology (MIT), Cambridge, MA USA; 6 Center of Excellence in Neuro-Oncology Sciences, St. Jude Children’s Research Hospital, Memphis, TN USA; 7 Department of Developmental Neurobiology, St. Jude Children’s Research Hospital, Memphis, TN USA; 8 Department of Biochemistry and Molecular Biology, Mayo Clinic College of Medicine and Science, Rochester, MN USA; 9 Norcliffe Foundation Center for Integrative Brain Research, Seattle Children’s Research Institute, Seattle, WA USA; 10 Department of Medical Biophysics, University of Toronto, Toronto, Ontario Canada; 11 Texas Children’s Cancer and Hematology Center, Houston, TX USA; 12 Department of Pediatrics—Hematology/Oncology, Baylor College of Medicine, Houston, TX USA; 13 Inserm, Paris, France; 14 Institut Curie, PSL Research University, Paris, France; 15 MINES ParisTech, CBIO—Centre for Computational Biology, PSL Research University, Paris, France; 16 Department of Biochemistry and Molecular Biology, University of Calgary, Calgary, Alberta Canada; 17 Department of Surgery, McGill University and RI-MUHC Cancer Research Program, Montreal, Quebec Canada; 18 Department of Pediatric and Adolescent Medicine, Mayo Clinic College of Medicine and Science, Rochester, MN USA; 19 Department of Pediatric Hematology-Oncology, Children’s Hospital of Minnesota, Minneapolis, MN USA; 20 Department of Neurology, Erasmus University Medical Center, Rotterdam, The Netherlands; 21 Department of Pathology, Erasmus University Medical Center, Rotterdam, The Netherlands; 22 Clinical Cooperation Unit Neuropathology, German Cancer Research Center (DKFZ), Heidelberg, Germany; 23 Division of Pediatric Neurooncology, German Cancer Research Center (DKFZ) and German Cancer Research Consortium (DKTK), Heidelberg, Germany; 24 Hopp Children’s Cancer Center (KiTZ), Heidelberg, Germany; 25 Princess Maxima Center for Pediatric Oncology, Utrecht, the Netherlands; 26 Utrecht University Medical Center (UMCU), Utrecht, the Netherlands; 27 Department of Neurosurgery, Hospital Infantil de Mexico Federico Gomez, Mexico City, Mexico; 28 Department of Pathology, Instituto Nacional de Pediatria, Mexico City, Mexico; 29 Department of Pathology and Molecular Medicine, McMaster University, Hamilton, Ontario Canada; 30 Department of Surgery, McMaster University, Hamilton, Ontario Canada; 31 Cancer and Blood Disorders Center, Seattle Children’s Hospital, Seattle, WA USA; 32 Division of Pediatric Neurosurgery, Seoul National University Children’s Hospital, Seoul, Republic of Korea; 33 Neuro-Oncology Clinic, National Cancer Center, Goyang, Republic of Korea; 34 Department of Neurosurgery, Tohoku University Graduate School of Medicine, Sendai, Japan; 35 Department of Neurological Surgery, University of California San Francisco, San Francisco, CA USA; 36 Department of Cellular and Molecular Pharmacology, University of California San Francisco, San Francisco, CA USA; 37 Princess Margaret Cancer Centre, University Health Network, Toronto, Ontario Canada; 38 Department of Pediatric Pathology and Neuropathology, University of Miami Miller School of Medicine, Miami, FL USA; 39 Department of Neurosurgery, University of Debrecen, Debrecen, Hungary; 40 Department of Neurological Surgery, University of Pittsburgh School of Medicine, Pittsburgh, PA USA; 41 Department of Pathology, University of Pittsburgh School of Medicine, Pittsburgh, PA USA; 42 Department of Neurosurgery, University of Ulsan Asan Medical Center, Ulsan, Republic of Korea; 43 Department of Pathology, The Children’s Memorial Health Institute, Warsaw, Poland; 44 Department of Oncology, The Children’s Memorial Health Institute, Warsaw, Poland; 45 Department of Neurological Surgery, Vanderbilt Medical Center, Nashville, TN USA; 46 Department of Pediatrics, Emory University, Atlanta, GA USA; 47 Department of Neurology, Vanderbilt Medical Center, Nashville, TN USA; 48 Division of Experimental Medicine, McGill University, Montreal, Quebec Canada; 49 Department of Human Genetics, McGill University, Montreal, Quebec Canada; 50 Department of Pediatrics, Baylor College of Medicine, Houston, TX USA; 51 Cancer and Hematology Center, Texas Children’s Hospital, Houston, TX USA; 52 Dan L Duncan Comprehensive Cancer Center, Baylor College of Medicine, Houston, TX USA; 53 Division of Brain Tumor Translational Research, National Cancer Center Research Institute, Tokyo, Japan; 54 Department of Pediatrics, University of Washington, Seattle, WA USA; 55 Department of Neurosurgery, Baylor College of Medicine, Houston, TX USA; 56 Department of Neurosurgery, Texas Children’s Hospital, Houston, TX USA

**Keywords:** Epigenomics, Oncogenes, CNS cancer, Mutagenesis

## Abstract

Transcription factors are frequent cancer driver genes, exhibiting noted specificity based on the precise cell of origin. We demonstrate that *ZIC1* exhibits loss-of-function (LOF) somatic events in group 4 (G4) medulloblastoma through recurrent point mutations, subchromosomal deletions and mono-allelic epigenetic repression (60% of G4 medulloblastoma). In contrast, highly similar SHH medulloblastoma exhibits distinct and diametrically opposed gain-of-function mutations and copy number gains (20% of SHH medulloblastoma). Overexpression of ZIC1 suppresses the growth of group 3 medulloblastoma models, whereas it promotes the proliferation of SHH medulloblastoma precursor cells. SHH medulloblastoma ZIC1 mutants show increased activity versus wild-type ZIC1, whereas G4 medulloblastoma ZIC1 mutants exhibit LOF phenotypes. Distinct *ZIC1* mutations affect cells of the rhombic lip in diametrically opposed ways, suggesting that *ZIC1* is a critical developmental transcriptional regulator in both the normal and transformed rhombic lip and identifying *ZIC1* as an exquisitely context-dependent driver gene in medulloblastoma.

## Abstract (web summary)

Analysis of medulloblastomas in humans and mice shows that the functional consequences of *ZIC1* mutations are exquisitely dependent on the cells of origin that give rise to different subgroups of medulloblastoma.

## Main

Malignant transformation of the human rhombic lip results in medulloblastoma, with group 3 (G3), group 4 (G4) and sonic hedgehog (SHH) tumors arising from the upper rhombic lip, and wingless/integrated (WNT) medulloblastoma arising from the lower rhombic lip^1–13^. There are a number of well-known driver genes for medulloblastoma, particularly SHH pathway genes in SHH medulloblastoma. However, G4 medulloblastoma is less well understood, with mutations of histone modifier genes, members of the *CBFA* complex and amplifications of *MYCN* and *OTX2* (refs. ^3,14^). A tail of less well understood but recurrent somatically altered genes has been observed across medulloblastoma subgroups^14^.

The zinc finger protein in the cerebellum (ZIC) family of transcription factors (TFs) has crucial roles in the development of the central nervous system (CNS), including hindbrain development^15–17^. There are five human ZIC family genes (*ZIC1*–*ZIC5*), all of which contain conserved tandem C2H2 zinc finger motif repeats that can interact with DNA or other proteins^15–18^. While ZICs exhibit some overlapping expression patterns throughout the CNS, different mutations are associated with distinct congenital disorders^15,16,19^. Somatic mutations of *ZIC1* have been identified in distinct medulloblastoma subgroups, and although *ZIC1* is a pan-medulloblastoma master TF associated with an active super-enhancer (SE)^20^, the specific role of ZIC TFs in the etiology of medulloblastoma is obscure.

*ZIC1* and *ZIC4* have multiple critical roles in cerebellar development^15,16,21^. Heterozygous deletion of the *ZIC1*/*ZIC4* locus in humans^22^ is a rare cause of Dandy–Walker malformation (DWM), which includes cerebellar hypoplasia^16^. Gain-of-function (GOF) mutations at the carboxy terminus of *ZIC1* have been identified in children with craniosynostosis and learning disabilities^23^. We now demonstrate that *ZIC1* mutations in medulloblastoma are context dependent, with loss-of-function (LOF) mutations and epigenetic alterations in G4 medulloblastoma, contrasted with GOF mutations in SHH medulloblastoma. Concordantly, expression of *ZIC1* represses malignant phenotypes in G3/G4 medulloblastoma while enhancing malignant phenotypes in SHH medulloblastoma in model systems. *ZIC1* is therefore a stark example of how the same gene can have distinct driver mechanisms in highly similar cancers depending on their specific lineage of origin.

## Results

### The subgroup-specific H3K27ac/H3K27me3 landscape of medulloblastoma

**Fig. 1:**
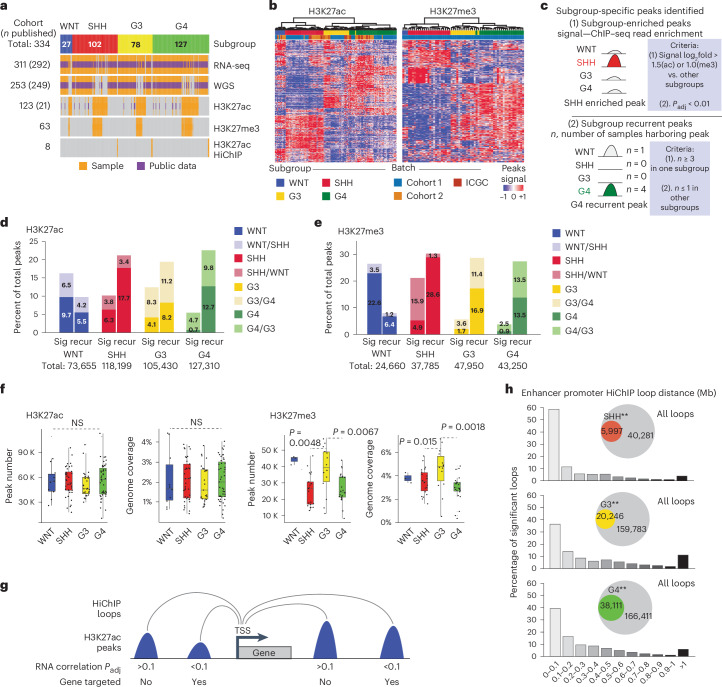
Characterization of subgroup-specific chromatin landscape of medulloblastoma. **a**, Summary of the newly generated and public datasets. Number within the bracket indicates the number of tumors with previously published data. **b**, Hierarchical clustering plots generated using the top 10,000 variable H3K27ac and H3K27me3 ChIP–seq peaks. **c**, Schematic representation summarizing different types of ChIP–seq peaks used in downstream analysis. Subgroup-specific peaks were defined by identifying peaks that (1) exhibit subgroup enrichment in ChIP–seq read counts or (2) are recurrently present only for specific subgroups even if the average ChIP–seq read count is not strongly subgroup enriched on average. **d**, Number of subgroup-specific peaks for each subgroup in the H3K27ac cohort. After batch correction, peaks annotated as subgroup enriched for ChIP–seq reads or subgroup recurrent were characterized separately. **e**, Number of subgroup-specific H3K27me3 peaks using the same annotations/criteria as **d**. **f**, Number of peaks and proportion of genome covered by H3K27ac and H3K27me3 peaks across the medulloblastoma subgroups. *P* values were calculated by the tailed Mann–Whitney *U* test. Biological sample size for H3K27ac—G3/G4/SHH/WNT = 27/47/39/10 and H3K27me3—G3/G4/SHH/WNT = 14/24/22/3. Center of box, median. Bounds of box, 25% and 75% percentile. Whiskers show minimum and maximum values within the 1.5× interquartile range. **g**, Schematic representation summarizing how high-confidence enhancer–promoter interactions were identified from HiChIP and ChIP–seq data. Adjusted *P* values were calculated using Pearson correlation between target gene transcript and enhancer H3K27ac read levels, which was corrected for multiple testing. **h**, Summary of distance distribution for high-confidence enhancer–promoter interactions. Proportion of SCLs (**g**; Methods) over a total number of loops is depicted as overlapping Venn diagrams. Double asterisk (**) indicates a significant correlation (P.adj < 0.1).

**Extended Data Fig. 1:**
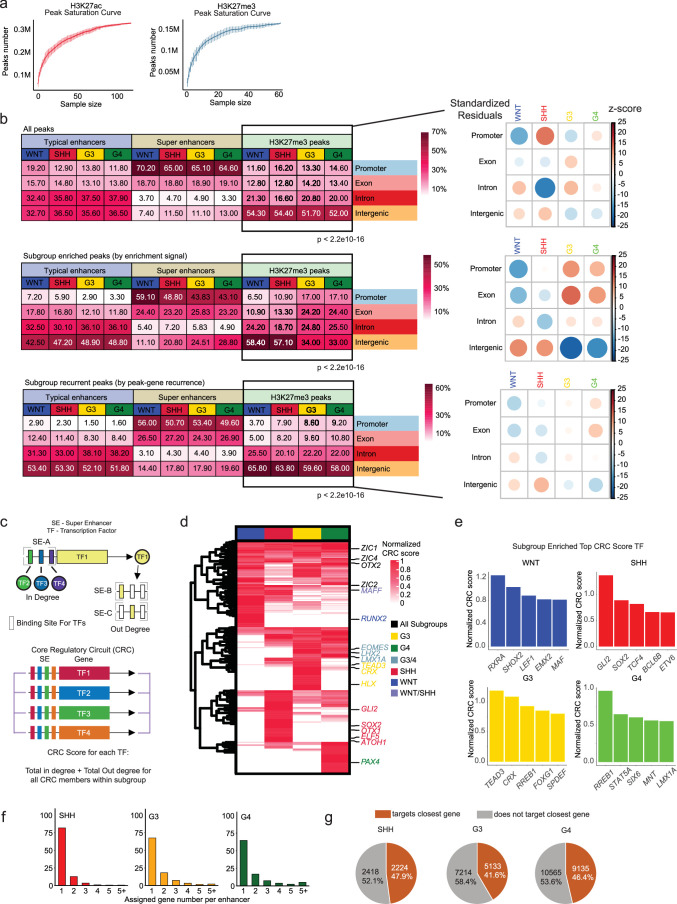
Medulloblastoma exhibits subgroup-specific master transcription factors (TFs) and chromatin landscape. **a**, Saturation analysis for H3K27ac and H3K27me3 peak identification. For each number of samples shown on the x axis, a subset of total cohort of ChIP–seq samples corresponding to this number was randomly selected. Number of non-overlapping peaks identified from this subset were recorded for each iteration of random sampling. Average and standard deviation for 10 iterations were plotted for each number up to total cohort size. Number of peaks identified starts to plateau toward the end of the curve, suggesting that addition of new samples will likely lead to diminishing returns. **b**, Annotation for typical enhancers, super-enhancers (SE) and H3K27me3 peaks that are classified as (1) all peaks found in the subgroup, (2) subgroup-enriched peaks (defined in Fig. 1c) and (3) subgroup-recurrent peaks (defined in Fig. 1c). P values were calculated by performing two-tailed chi-square test on H3K27me3 peaks. Standardized residuals for chi-square tests performed on H3K27me3 peak distributions were also calculated. **c**. Strategy used to define core regulatory circuit (CRC) score for each transcription factor for each subgroup. In degree (number of TFs that target the TF of interest) and out degree (number of TF promoters targeted by the TF of interest) were calculated for each TF to identify subgroup-specific and pan-subgroup core TFs. **d**, Heatmap summarizing pan subgroup and subgroup-specific core TFs crucial for shaping core circuitry landscape for each subgroup. **e**, Top 5 subgroup-specific master transcription factors identified for each subgroup according to CRC score. **f**, Number of genes assigned for each enhancer across enhancer–promoter interactions identified using HiChIP and 27ac ChIP–seq data. **g**, Proportion of enhancers that target the closest genes for SHH, G3 and G4 subgroups.

Due to the high prevalence and recurrence of somatic mutations in genes associated with chromatin modulation in medulloblastoma (~30% of medulloblastomas)^14^, we hypothesized that some medulloblastomas might acquire somatic histone modification alterations (chromatin variants^24,25^) for driver genes. To test this hypothesis, we profiled H3K27ac and H3K27me3 landscapes across the four medulloblastoma subgroups (including 123 matching samples for H3K27ac and 63 matching samples for H3K27me3) and integrated the data with matching RNA sequencing (RNA-seq), as well as an independent cohort of tumors characterized by H3K27ac HiChIP (Fig. 1a, Extended Data Fig. 1a and Supplementary Tables 1 and 2). Hierarchical clustering using either H3K27ac or H3K27me3 chromatin immunoprecipitation followed by sequencing (ChIP–seq) data recapitulated the four subgroups (Fig. 1b). We categorized subgroup-specific H3K27 modification as either subgroup-enriched peaks (signal enrichment) or subgroup-recurrent peaks (peak called recurrently for one subgroup; Fig. 1c–e). A subset of the identified peaks was shared by either SHH/WNT (enriched in SHH versus G3 or G4, but not WNT) or G3/G4 (enriched in G3 versus SHH or WNT, but not G4; Fig. 1d, e) and were documented as such.

The average number of peaks and the proportion of genome coverage for H3K27ac did not significantly differ between subgroups (Fig. 1f). However, H3K27me3 deposition was markedly increased in G3 medulloblastoma (Fig. 1f). Additionally, G3/G4 medulloblastoma-enriched H3K27me3 peaks exhibited a strong preference for gene promoters as compared to WNT/SHH (Extended Data Fig. 1b). Core regulatory circuit analysis of H3K27ac ChIP–seq data identified known and new medulloblastoma subgroup-specific master TFs, including the pan-subgroup master TFs *ZIC1* and *ZIC4* as we reported previously (Extended Data Fig. 1c–e)^20^. Additionally, H3K27ac HiChIP was used to define the enhancer–promoter interactome across medulloblastoma subgroups (Fig. 1g). Integration of H3K27ac HiChIP, H3K27ac ChIP–seq and RNA-seq allowed the identification of loops connecting enhancers and promoters of protein-coding genes. Among the enhancer–promoter interacting loops, those with enhancer H3K27ac read counts exhibiting significant positive correlations with the expression of target genes were also identified (adjusted *P* < 0.1) and defined as significantly correlated loops (SCL; Fig. 1g,h). Many SCL-associated enhancers target more than one gene (Extended Data Fig. 1f), and notably, enhancers frequently target genes that are not the most proximal gene (Extended Data Fig. 1g).

We conclude that post-translational modification of H3K27 in medulloblastoma varies by subgroup.

### Recurrent single-nucleotide variations (SNVs) and hemizygous H3K27me3 affect *ZIC1* in G4 medulloblastoma

**Fig. 2:**
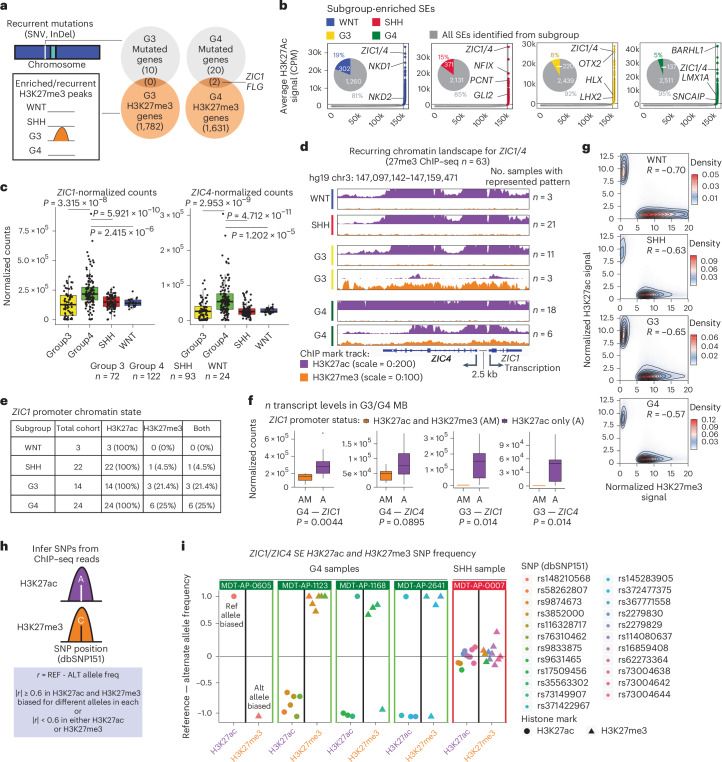
*ZIC1* is recurrently mutated and repressed by H3K27me3 in G4 medulloblastoma. **a**, Overlap between genes regulated by subgroup-specific H3K27me3 peaks in G3, G4 medulloblastoma and genes recurrently mutated in each subgroup. **b**, Ranking of SEs across medulloblastoma subgroups, showcasing the number of total SEs identified (in gray) as well as the proportion of subgroup-enriched SEs in pie charts. **c**, *ZIC1* and *ZIC4* expression patterns across medulloblastoma subgroups. Biological sample size—G3/G4/SHH/WNT = 72/122/93/24. *P* values from two-tailed Mann–Whitney *U* test. Center of box, median. Bounds of box, 25% and 75% percentile. Whiskers show minimum and maximum values within 1.5× interquartile range. **d**, Sequencing depth normalized bigwig tracks showcasing recurrent (*n* ≥ 3 per subgroup) *ZIC1* and *ZIC4* chromatin states across four subgroups. **e**, Summary of chromatin states observed at the *ZIC1* promoter across all samples in the ChIP–seq libraries with both H3K27ac and H3K27me3 modifications. **f**, Expression levels of *ZIC1* and *ZIC4* in G3/G4 medulloblastoma samples that harbor both H3K27ac and H3K27me3 (AM) or just H3K27ac (A) peaks on the *ZIC1* promoter. Biological sample size for G4—AM/A = 6/18 (24 total) and G3—AM/A = 3/11 (14 total). *P* values from two-tailed Mann–Whitney *U* test. Same whisker box plot parameters as **c**. **g**, Density plot summarizing H3K27ac versus H3K27me3 signal at H3K27ac and H3K27me3 peaks. Correlations between H3K27ac and H3K27me3 were calculated by Pearson correlation on merged peak coordinates. **h**, Method for inferring heterozygous SNPs using H3K27ac and H3K27me3; two mutually exclusive histone modification marks. **i**, Distribution of inferred heterozygous SNPs across H3K27ac and H3K27me3 libraries of four G4 samples and one SHH sample with H3K27ac and H3K27me3 peaks on the *ZIC1* promoter.

**Extended Data Fig. 2:**
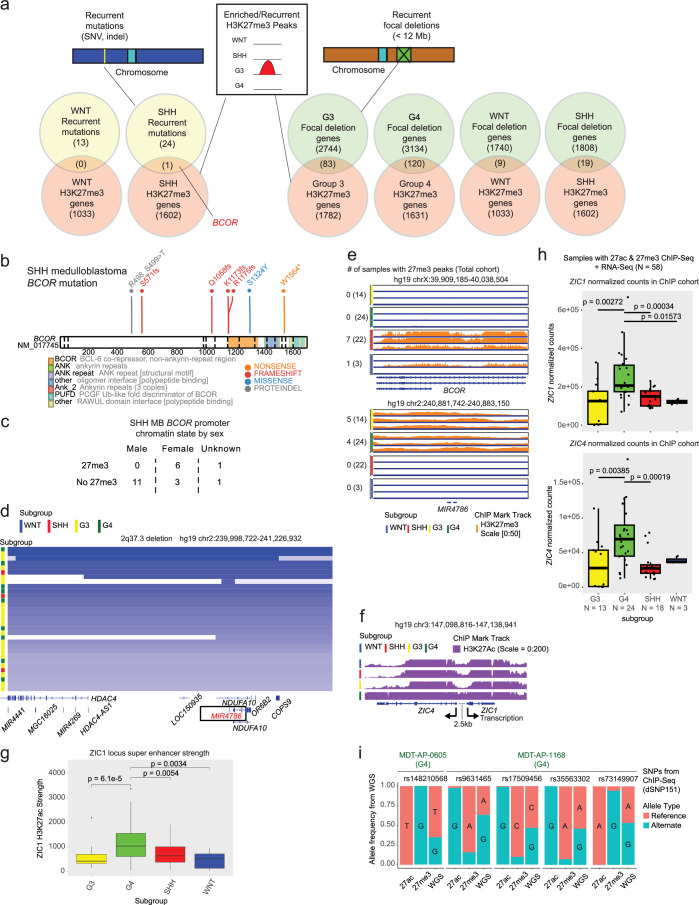
Overlap between recurrent copy number deletions and subgroup enriched/recurrent H3K27me3 peaks for group 3 (G3)/group 4 (G4) medulloblastoma. **a**, Venn diagram depicting overlap between subgroup-enriched H3K27me3 peaks with recurrently mutated genes in WNT, SHH as well as genes recurrently affected by focal deletion (<12 Mb) in all 4 subgroups (Supplementary Table 13). **b**, *BCOR* mutation pattern identified in SHH medulloblastoma. **c**, Breakdown of *BCOR* H3K27me3 pattern in SHH medulloblastoma. Highly female-enriched pattern is observed, suggesting that X inactivation may have a role in the observed chromatin phenomenon. **d**, Showcase of recurrent deletion of 2q37.3 locus identified in G3 and G4. *MIR4786* locus exhibits a G3/G4-enriched copy loss pattern (Supplementary Table 13). **e**, Representative H3K27me3 ChIP–seq signal patterns for all subgroups on *BCOR* and *MIR4786* locus, which exhibit SHH-enriched and G3/G4-enriched H3K27me3 signal, respectively (Supplementary Tables 12 and 13). **f**, Read depth normalized 27ac bigwig tracks for a representative sample from each subgroup. Bidirectional promoters regulating *ZIC1* and *ZIC4* transcription are regulated by a common super-enhancer identified across all subgroups. **g**, H3K27ac signal strength of SE overlapping *ZIC1*/*4* promoter across MB subgroups. Biological sample size: G3/G4/SHH/WNT = 27/47/39/10. Center of box—median. Bounds of box—25% and 75% percentile. Whiskers show minimum and maximum values within the 1.5× interquartile range. P values from two-tailed Mann–Whitney *U* test. **h**, *ZIC1-* and *ZIC4*-normalized transcript count levels in ChIP cohort samples with matching H3K27ac, H3K27me3 and RNA-seq data (N = 58). Biological sample size: G3/G4/SHH/WNT = 13/24/18/3. Box plot parameters same as **g**. P values from two-tailed Wilcoxon rank-sum test. **i**, Allelic frequencies for the inferred heterozygous single-nucleotide polymorphisms (from Fig. 2h,i) in 2 G4 samples with matching WGS data.

We hypothesized that a subset of medulloblastoma LOF driver genes somatically altered by SNVs, small insertions/deletions (InDels) or copy number aberrations (CNAs) might also be targeted through somatic H3K27me3-mediated repression to achieve the common endpoint of tumor suppressor gene LOF. We determined the intersection between genes affected by genetic mutations and those overlapping either ‘enriched’ or ‘recurrent’ subgroup-specific H3K27me3 peaks (Fig. 2a and Extended Data Fig. 2a)^14^. While no overlapping genes were identified for WNT or G3, *BCOR* for SHH, and both *ZIC1* and *FLG* in G4 are affected by both mutation and H3K27me3-modified chromatin. H3K27me3 peaks on the *BCOR* promoter (chromosome Xp11.4) were found predominantly in female SHH tumors, suggesting a link to X chromosome inactivation (Extended Data Fig. 2b,c). Broadening the analysis to genes encompassed by focal deletions identified from our published Affymetrix SNP6 array data^26,27^ identified genes targeted by both deletions and H3K27me3, including the MIR4786 locus in G3 and G4 medulloblastoma (Extended Data Fig. 2d,e and Supplementary Tables 3–13).

The *ZIC1* and *ZIC4* genomic loci are separated by an interposed, shared, bidirectional promoter (Extended Data Fig. 2g). They are coregulated by a SE that is highly active across all four subgroups (Fig. 2b and Extended Data Fig. 2f,g). Both genes are highly expressed across all medulloblastoma subgroups as previously described^20^, particularly in the G4 (Fig. 2c and Extended Data Fig. 2h). We now describe a subset of G3 and G4 tumors that exhibit atypical hemizygous H3K27me3 deposition across the *ZIC1*/*ZIC4* SE locus while showing a robust H3K27ac mark in *trans* on the other allele (Fig. 2d,e). This pattern was associated with reduced *ZIC1*/*ZIC4* transcript levels (Fig. 2f) and was not recurrently observed in either SHH or WNT medulloblastoma (Fig. 2e). These two functionally opposing marks are usually mutually exclusive at the vast majority of loci, with the ‘H3K27ac–H3K27me3 hemizygous state’ being exceedingly rare (Fig. 2g). We hypothesized therefore that somatic repression of *ZIC1* through acquisition of the ‘H3K27ac–H3K27me3 hemizygous state’ is a chromatin-based driver event in G4 medulloblastoma.

To determine if the H3K27ac and H3K27me3 are indeed found in *trans* on separate alleles within the same cells, allelic frequencies for dbSNP151 annotated heterozygous single-nucleotide polymorphisms (SNPs) were examined in our H3K27ac and H3K27me3 libraries for samples harboring the H3K27ac–H3K27me3 hemizygous state at the *ZIC1*/*ZIC4* locus (Fig. 2h). While the G3 samples lacked heterozygous SNPs, all SNPs within the examined G4 samples exhibited a strong bias for distinct alleles in the H3K27ac versus H3K27me3 libraries (Fig. 2i), suggesting that the two chromatin marks occur in *trans* within single cells. Inferred SNPs were verified with matching whole-genome sequencing (WGS) data when possible (Extended Data Fig. 2i). While a plurality of G4 medulloblastomas alter activity of *ZIC1* through genetic mutation, an additional nonoverlapping cohort (Supplementary Table 1) of G4 tumors reduce *ZIC1*/*ZIC4* expression through uni-allelic chromatin variant repression mediated by H3K27me3 deposition, suggesting a convergence of mechanisms underlying *ZIC1* alteration and that *ZIC1* might be a LOF driver gene in G4 medulloblastoma.

### Mono-allelic SEs regulate *ZIC1*/*ZIC4* expression in G3/G4 medulloblastoma

**Fig. 3:**
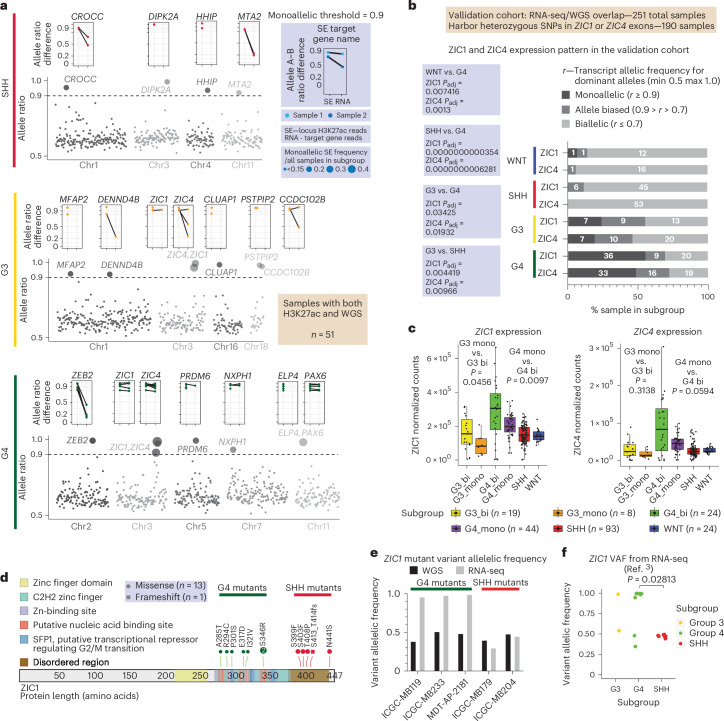
*ZIC1*/*ZIC4* exhibit mono-allelic expression patterns in G3 and G4. **a**, SEs that are recurrently (*n* ≥ 3 for G4 and *n* ≥ 2 for others) mono-allelic across different medulloblastoma subgroups. SEs that harbor SNPs (phased and pooled for each allele) that are heterozygous in WGS but homozygous (normalized allelic frequency ≥ 0.9) in H3K27ac ChIP–seq reads (same SNPs) from the same sample were defined as mono-allelic. Dot plots above each SE show differences in pooled allelic frequencies for heterozygous SNPs (allele A–B) in (1) H3K27ac reads from the SE (left) and (2) RNA-seq reads from the SE target gene (right). Matching samples are connected by lines between SE and RNA. **b**, Allelic frequency summary for heterozygous germline SNPs for *ZIC1* and *ZIC4* transcripts in RNA-seq within the validation cohort (251 samples with both WGS and RNA-seq data). Adjusted *P* values from two-tailed pairwise Fisher’s exact test. **c**, Whisker box plots summarizing *ZIC1* and *ZIC4* expression cross the medulloblastoma subgroups, but G3 and G4 are divided according to mono-allelic (mono) versus bi-allelic (bi) expression of *ZIC1* or *ZIC4*. Biological sample size: G3_bi/G3_mono = 19/8, G4_bi/G4_mono = 24/44 and SHH/WNT = 93/24. *P* values from two-tailed Mann–Whitney *U* test. Center of box, median. Bounds of box, 25% and 75% percentile. Whiskers show minimum and maximum values within the 1.5× interquartile range. **d**, Mutational landscape of *ZIC1* in G4 and SHH. **e**, Allelic frequency distribution for *ZIC1* mutations in G4 (*n* = 3) and SHH (*n* = 2) samples from the assembled validation cohort. **f**, *ZIC1* VAF obtained from published medulloblastoma RNA-seq data. *P* value from two-tailed Mann–Whitney *U* test.

**Extended Data Fig. 3:**
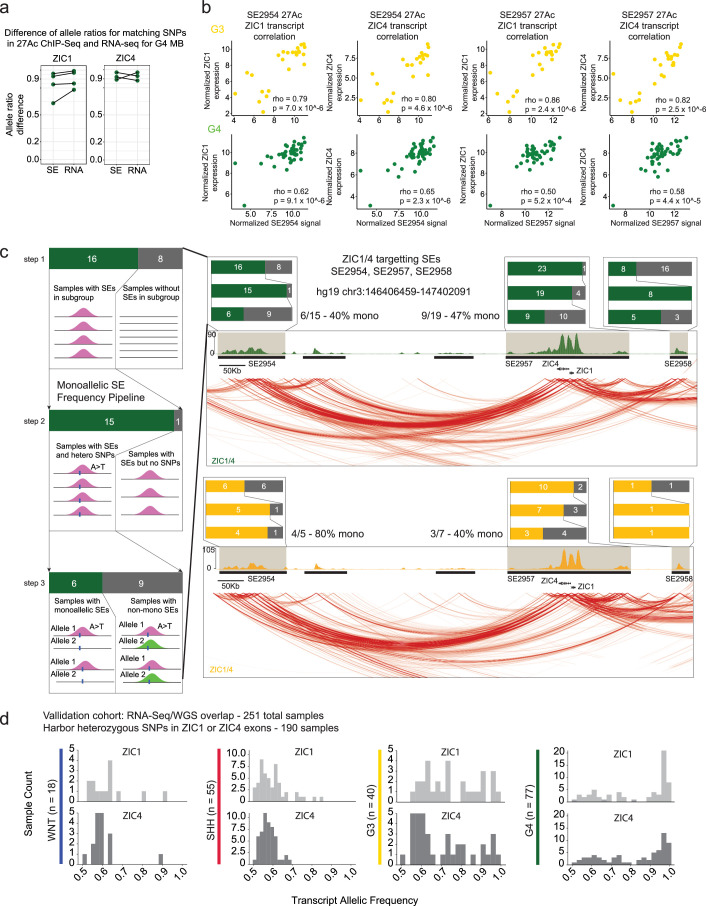
*ZIC1*/*4* locus is regulated by multiple super-enhancers (SE) that are recurrently epigenetically repressed on single alleles. **a**, Allelic frequencies for heterozygous SNPs present in both H3K27ac ChIP–seq reads on *ZIC1*/*4* SE as well as RNA-seq reads on *ZIC1*/*4* exons. Identical schematic to dot plots from Fig. 3a, but only the exact match heterozygous SNPs identified in both H3K27ac ChIP–seq and RNA-seq data were used. Matching samples are connected by lines between SE and RNA columns. Y axis shows difference in pooled allelic frequency between SNPs from the two different alleles. *ZIC1*/*4* RNA and SE exhibit bias for the same alleles from the heterozygous single-nucleotide polymorphisms (SNPs), suggesting that the monoallelic SE drives monoallelic expression. **b**, Correlation between H3K27ac reads on two SEs that target ZIC1/4 locus (from Extended Data Fig. 2g), SE2954 and SE2957, and *ZIC1*/*ZIC4* transcript levels in group 3 (G3) and group 4 (G4) medulloblastoma. P values generated from two-tailed Spearman correlation analysis. **c**, *ZIC1*/*4* targeting SEs, their interaction maps with ZIC1/4 locus and frequency of their monoallelic status in G3 and G4 medulloblastoma. SE directly on top of *ZIC1*/*4* genes (SE2957) was monoallelic in 9 out of 19 samples in G4 and 3 out of 7 samples in G3. SEs upstream (SE2954) and downstream (SE2958) of *ZIC1*/*4* locus are also recurrently monoallelic and were identified as high-confidence enhancer–promoter interactions with HiChIP, H3K27ac ChIP–seq and RNA-seq data. While most samples harbored SE2957, a smaller proportion of G3 and G4 samples harbored SE2954 and SE2958. **d**, Allelic frequency distribution of heterozygous germline SNPs for *ZIC1* and *ZIC4* transcripts in RNA-seq within the validation cohort (total of 251 samples with both WGS and RNA-seq data). A total of 190 samples contain heterozygous SNPs within *ZIC1*/*4* exons in both normal control and tumor DNA.

Our observation that the *ZIC1*/*ZIC4* locus undergoes recurrent repression in G4 medulloblastoma through hemizygous deposition of H3K27me3 on its SE prompted us to look for additional mono-allelic SEs in a cohort of 51 medulloblastoma tumors with matching H3K27ac ChIP–seq and WGS data (Fig. 3a). Mono-allelic SEs were rare in SHH medulloblastoma, although a number of further examples were identified for G3 and G4 medulloblastoma, including the known example of *PRDM6* enhancer hijacking in G4 (Fig. 3a)^14^. Of the 19 G4 medulloblastoma samples harboring heterozygous SNPs at the *ZIC1*/*ZIC4* SE locus (to allow assessment of heterozygosity), 9/19 tumors (47% of cases) exhibited a mono-allelic SE in keeping with the H3K27ac–H3K27me3 hemizygous state. A similar, albeit less frequent pattern, was observed in G3 medulloblastoma, but only very rarely in SHH medulloblastoma. Notably, samples with mono-allelic *ZIC1*/*ZIC4* SE exhibit expression of *ZIC1*/*ZIC4* mRNA predominantly from the H3K27ac allele (Extended Data Fig. 3a), in keeping with a bona fide repression effect of H3K27me3 deposition. Aside from the SE directly overlapping the *ZIC1*/*ZIC4* locus, several other genomically proximate SEs that target *ZIC1*/*ZIC4* were also identified to be recurrently mono-allelic (Extended Data Fig. 3b, c).

We determined the mono-allelic expression pattern of *ZIC1*/*ZIC4* in a validation cohort of 251 medulloblastomas with matching RNA-seq and WGS data, assembled by combining publicly available and newly generated datasets^3,4,14,27,28^. We found frequent mono-allelic expression in G3 and G4, but neither SHH nor WNT medulloblastomas (Fig. 3b). Indeed, 55% of G4 tumors (36/65) and 24% of G3 tumors (7/29) exhibit mono-allelic expression of *ZIC1*, and 48.5% (33/68) of G4 tumors and 18.9% (7/37) of G3 tumors have mono-allelic expression of *ZIC4* (Fig. 3b and Extended Data Fig. 3d). In both G3 and G4, mono-allelic expression is associated with reduced expression of *ZIC1*/*ZIC4*, consistent with chromatin-based suppression (Fig. 3c). The importance of diminished, mono-allelic expression of the *ZIC1*/*ZIC4* locus in medulloblastomas arising from the rhombic lip is underscored by humans who have hypoplastic cerebella (DWM) secondary to germline hemizygous deletions of *ZIC1*/*ZIC4* (ref. ^16^). We conclude that haploinsufficiency of *ZIC1* due to either germline or somatic events, with consequent diminished transcription, has critical effects on the biology of the rhombic lip, either in toto (DWM) or possibly in distinct somatic subclones (medulloblastoma).

*ZIC1* is a presumed medulloblastoma driver gene that recurrently harbors SNVs in G4 and SHH medulloblastoma^14^. We now demonstrate that *ZIC1* mutations in G4 medulloblastoma are found in the DNA-binding zinc finger domain, whereas SHH medulloblastoma SNVs are found in the 3′ end of the gene, encoding a carboxy-terminal intrinsically disordered region (IDR) of currently unknown function (Fig. 3d)^14^. Intriguingly, SHH medulloblastoma *ZIC1* somatic mutations are found in the same 3′ region of the *ZIC1* gene as previously reported germline GOF *ZIC1* mutations in humans with craniosynostosis^23^. Within our 251 medulloblastoma validation cohort, three G4 tumors and two SHH tumors with *ZIC1* mutations were identified. In all three G4 tumors, the variant allele frequency (VAF) of mutants comprised nearly 100% of all *ZIC1* reads from RNA-seq, whereas they were below 50% in the matching WGS libraries (Fig. 3e). Conversely, SHH medulloblastoma mutants exhibited VAF near 50% in both WGS and RNA-seq reads. Examination of *ZIC1* VAF from our published medulloblastoma RNA-seq cohort^3,27^ produced similar results (Fig. 3f). These data are consistent with a model in which G4 medulloblastomas acquire LOF genetic and chromatin variants, while SHH medulloblastomas acquire GOF variants.

### Mono-allelic *ZIC1* expression occurs in a subset of G4 medulloblastoma

**Fig. 4:**
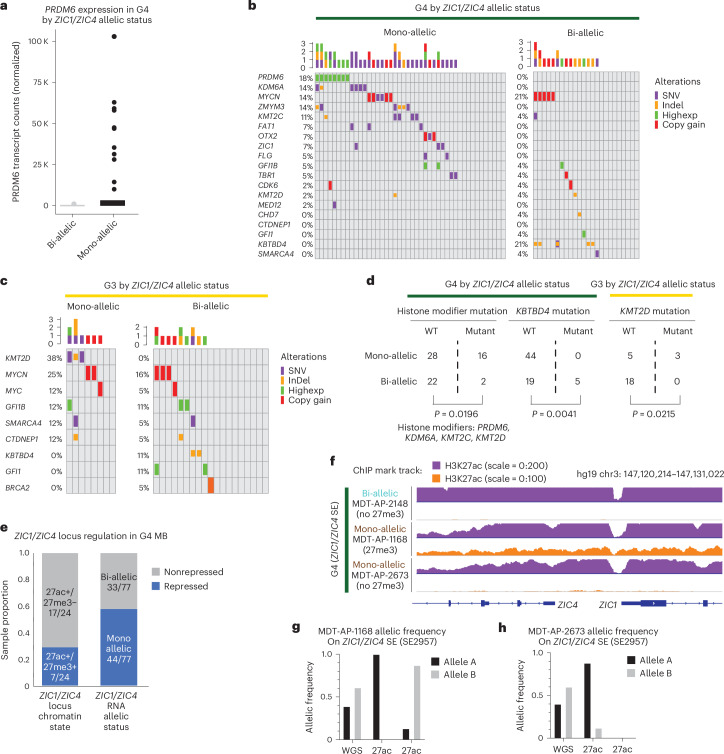
*ZIC1*/*ZIC4* mono-allelic and bi-allelic G3/G4 medulloblastomas enrich for distinct mutations. **a**, Whisker box plot of normalized *PRDM6* transcript counts in bi-allelic versus mono-allelic *ZIC1*/*ZIC4* G4 samples. *PRDM6* transcription occurs exclusively in the context of single allele inactivation of *ZIC1*/*ZIC4*. **b**, Oncoplot showcasing mutation status of previously published recurrently mutated genes in mono-allelic and bi-allelic G4 samples. Each column represents different samples. Each row represents different genes that are recurrently mutated in medulloblastoma. Distinct types of mutations for a gene in each patient are depicted with different size/colored bars. **c**, Oncoplot showcasing mutation status of previously published recurrently mutated genes in mono-allelic and bi-allelic G3 samples. **d**, Sample distribution summary and two-tailed Fisher’s exact test outputs for the significance of enrichment for chromatin modifier mutations in *ZIC1*/*ZIC4* mono-allelic G4 and G3, as well as *KBTBD4* mutation in *ZIC1*/*ZIC4* bi-allelic G4. **e**, Summary of different proportions of G4 medulloblastoma samples exhibiting transcriptional repression within the chromatin (H3K27me3) data or RNA (mono-allelic expression) data. **f**, Sequencing depth normalized bigwig tracks for H3K27ac and H3K27me3 in one G4 sample with bi-allelic *ZIC1*/*ZIC4* SE and two G4 samples with mono-allelic *ZIC1*/*ZIC4* locus SE. Not all G4 samples with mono-allelic *ZIC1*/*ZIC4* SE harbor H3K27me3 peak on the locus. **g**,**h**, Allelic frequencies for heterozygous SNPs in WGS, H3K27ac and H3K27me3 ChIP–seq data in the two mono-allelic G4 samples: MDT-AP-1168, where H3K27me3 is observed, and MDT-AP-2673, where H3K27me3 is absent on the *ZIC1*/*ZIC4* locus.

**Extended Data Fig. 4:**
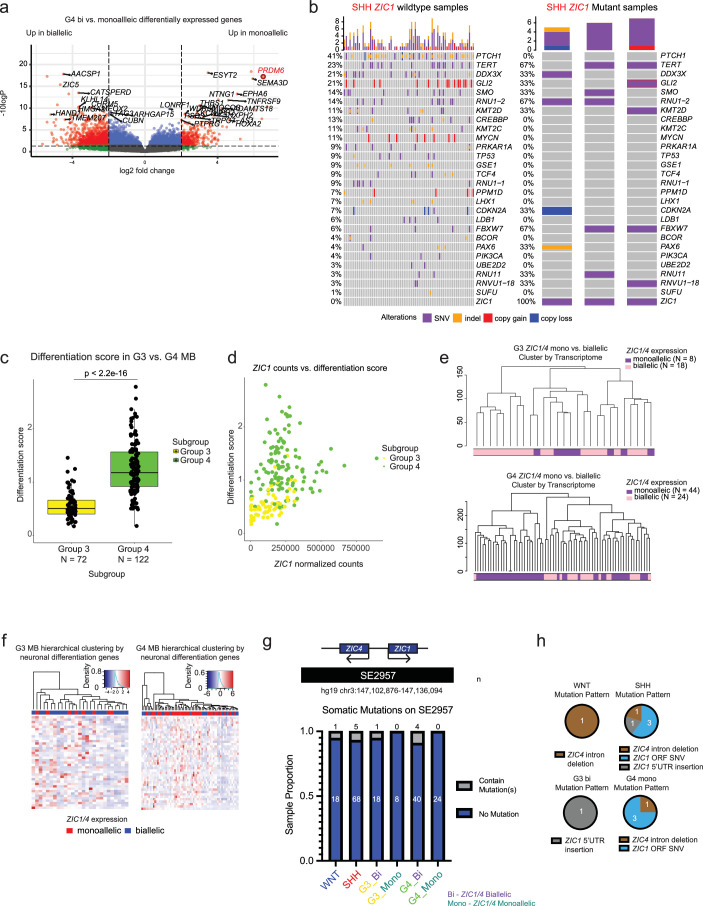
Genetic and transcriptional patterns associated with biallelic and monoallelic status of *ZIC1*/*4* across medulloblastoma. **a**, Volcano plot summarizing differentially expressed genes between *ZIC1*/*4* monoallelic and biallelic group 4 (G4) samples. Q value threshold of 0.01 and log_2_(fold change) threshold of 2 were used. **b**, Oncoplot summarizing the mutational landscape of SHH tumors with or without *ZIC1* mutations. *U1* snRNA mutations were always mutated together (RNU1-2, RNVU1-18) with *ZIC1*. **c**, Whisker box plot summarizing neuronal differentiation score for group 3 (G4) and G4 medulloblastoma tumors. Previously published 39 G3/G4 neuronal differentiation signature genes (Supplementary Table 14) were used to calculate the overall differentiation score for each tumor. Biological sample size: G3/G4 = 72/122. P value was calculated by two-tailed Mann–Whitney U test. Center of box—median. Bounds of box—25% and 75% percentile. Whiskers show minimum and maximum values within the 1.5× interquartile range. **d**, Scatter plot showing expression level of *ZIC1* across G3 and G4 medulloblastoma tumors vs. differentiation score in the same tumors. **e**, Hierarchical clustering of G3/G4 samples by top 10,000 variable genes from transcriptome. *ZIC1*/*4* monoallelic G3/G4 samples do not form distinct clusters from the biallelic samples. **f**, Hierarchical clustering of G3/G4 samples by expression level of the neuronal differentiation signature genes from **c**. *ZIC1*/*4* monoallelic G3/G4 samples do not form distinct clusters from the biallelic samples. **g**, Frequency of somatic mutations on super-enhancer (SE) on top of *ZIC1*/*4* locus (SE2957) across WNT, SHH, G3 *ZIC1*/*4* biallelic, monoallelic, G4 *ZIC1*/*4* biallelic and monoallelic samples. **h**, Breakdown of somatic mutation patterns on SE2957 for all subgroups.

*PRDM6* overexpression secondary to a tandem duplication of the *SNCAIP* locus is a suspected G4 medulloblastoma driver gene^14^, and in our dataset it is found only in G4 tumors with mono-allelic expression of *ZIC1* or *ZIC4* (Fig. 4a and Extended Data Fig. 4a). G4 *ZIC1*/*ZIC4* mono-allelic samples were significantly enriched (*P* = 0.0196) for mutations in chromatin modifiers including *KDM6A*, *KMT2C* and *KMT2D* (Fig. 4b). In G3, *KMT2D* mutation was significantly enriched (*P* = 0.0215) in *ZIC1*/*ZIC4* mono-allelic samples (Fig. 4c,d). Conversely, *KBTBD4* InDel mutations were enriched (*P* = 0.0041) in G3/4 *ZIC1*/*ZIC4* bi-allelic samples (Fig. 4b,c). SHH tumors with *ZIC1* mutations always co-occurred with mutations of the *U1* splicing factor (Extended Data Fig. 4b), consistent with our previous publication in which *ZIC1* mutations were found in SHHα and SHHδ tumors where *U1* mutations occur^27^. Notably, we observe cases of G4 medulloblastoma with mono-allelic *ZIC1*/*ZIC4* expression but without H3K27me3 deposition, suggesting that additional cryptogenic genetic/epigenetic routes to allelic silencing of *ZIC1*/*ZIC4* exist (Fig. 4e–h). G3/G4 medulloblastoma tumors exhibit a spectrum of ZIC1 expression levels as well as differentiation signatures (Supplementary Table 14), with G4 medulloblastoma exhibiting higher levels of both (Extended Data Fig. 4c,d), potentially rehighlighting the known role of ZIC1 in cerebellar development^29^.

One possible explanation for the H3K27ac–H3K27me3 hemizygous state is that it occurs naturally during the differentiation of the rhombic lip subventricular zone (RL-SVZ), where G4 medulloblastoma is thought to arise^2,3^. However, hierarchical clustering of G3 and G4 medulloblastoma by both overall transcriptome or neuronal gene expression does not segregate tumors by *ZIC1*/*ZIC4* expression status, suggesting that the observed repression of the *ZIC1*/*ZIC4* locus from chromatin variants is not purely secondary to a transient developmental state in the RL-SVZ (Extended Data Fig. 4e,f). mono-allelic *ZIC1*/*ZIC4* expression may also arise from local or distal mutations/structural variations affecting *ZIC1*/*ZIC4* transcription. However, mutational mining of the region surrounding the *ZIC1*/*ZIC4* locus for the presence of noncoding mutations that could account for the observed epigenetic repression failed to yield any likely candidates (Extended Data Fig. 4g,h). Taken together, we hypothesize that the acquisition of somatic mutations and/or aberrant activity of histone-modifying complexes may result in unusual regulation of the *ZIC1*/*ZIC4* locus, although this concept remains largely speculative.

### Opposing *ZIC1*/*ZIC4* CNAs in G3/G4 versus SHH medulloblastoma

**Fig. 5:**
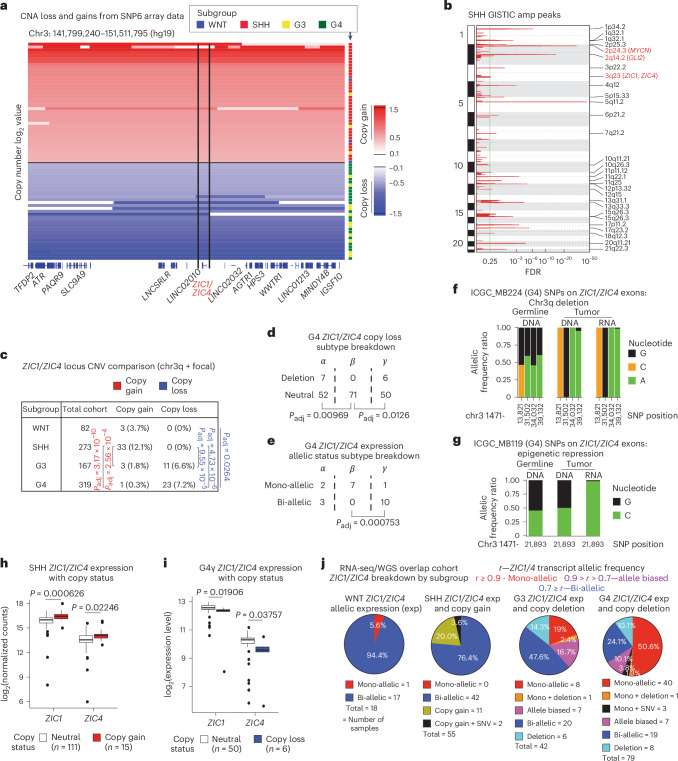
*ZIC1*/*ZIC4* locus exhibits distinct genomic rearrangements in G3/G4 and SHH medulloblastoma. **a**, CNA track for medulloblastoma samples exhibiting *ZIC1*/*ZIC4* locus copy gain/loss. **b**, GISTIC output for SHH medulloblastoma, highlighting 2p24.3 (*MYCN*), 2q14.2 (*GLI2*) and 3q23 (*ZIC1*/*ZIC4*) gain. FDR, false discovery rate. **c**, CNA summary for the *ZIC1*/*ZIC4* locus per medulloblastoma subgroups. Adjusted *P* values from two-tailed pairwise Fisher’s exact test. **d**, Chr3q and *ZIC1*/*ZIC4* focal copy deletion frequency across three subtypes of G4 medulloblastoma. *P* values from two-tailed Fisher’s exact test and Hochberg correction. **e**, Breakdown of chromatin repression of a single allele of *ZIC1*/*ZIC4* locus across three subtypes of G4 medulloblastoma. *P* values were calculated by two-tailed Fisher’s exact test followed by Hochberg multiple correction. **f**,**g**. Allelic frequencies for heterozygous germline SNPs across normal tumor DNA and tumor RNA from a representative G4 sample with (**f**) chr3q deletion and (**g**) epigenetic suppression of the *ZIC1*/*ZIC4* locus. **h**, Whisker box plots for *ZIC1* and *ZIC4* expression in SHH medulloblastoma tumors with chr3 copy gain versus neutral. Expression values from RNA-seq data with matching SNP6 array data. *P* values were calculated from the two-tailed Mann–Whitney *U* test. Center of box, median. Bounds of box, 25% and 75% percentile. Whiskers show minimum and maximum values within the 1.5× interquartile range. **i**, Whisker box plots for *ZIC1* and *ZIC4* expression in G4γ medulloblastoma with chr3q copy loss versus copy neutral. Expression values from expression array data with matching SNP6 array data. Same statistical test and whisker box plot parameters as **h**. **j**, Breakdown of *ZIC1*/*ZIC4* allelic expression pattern, *ZIC1*/*ZIC4* CNA and *ZIC1* SNVs in medulloblastoma samples with both RNA-seq and WGS data available, as well as harboring heterozygous germline SNPs in *ZIC1*/*ZIC4* exons.

Previous studies have reported recurrent copy loss of chromosome 3q (chr3q), which contains the *ZIC1*/*ZIC4* locus, in G4 medulloblastoma^26,30^. Examining CNAs at the *ZIC1*/*ZIC4* locus using published SNP6 array data^26^ validates this finding and further showcases an intriguing pattern—the *ZIC1*/*ZIC4* locus was recurrently deleted in G3/G4; however, the same locus exhibits recurrent genomic gains in SHH (Fig. 5a), as determined by GISTIC^31^, and pairwise comparison of CNAs across subgroups (Fig. 5b,c). Frequencies of chr3q deletions and focal deletions harboring the *ZIC1*/*ZIC4* locus within G4 medulloblastoma were examined at the subtype level as we annotated previously^30^. These deletions exhibited subtype specificity, being notably depleted in G4β (Fig. 5d), whereas chromatin-based repression of the locus is very frequent in G4β (Fig. 5e). Tumors that target *ZIC1* through either a genetic or a chromatin route show loss of heterozygosity at the level of mRNA (Fig. 5f,g). SHH samples affected by copy number gains exhibited concomitant increased expression of both *ZIC1* and *ZIC4* (Fig. 5h). SNP6 and expression array data^26,30^ demonstrate that G4γ samples with focal and broad deletions of the *ZIC1*/*ZIC4* locus exhibit diminished expression of *ZIC1* and *ZIC4* transcripts as compared to balanced controls (Fig. 5i). Because the *ZIC1*/*ZIC4* locus can be targeted by both genetic- and chromatin-based mechanisms, we examined the overall proportion of samples within the validation cohort medulloblastomas (251 tumors with RNA-seq and WGS) affected by either chromatin or genetic variants. We identified the copy number status for the *ZIC1*/*ZIC4* locus within these samples using control-FREEC on the WGS data^32^. Annotating samples by *ZIC1*/*ZIC4* allelic expression status, copy gain within SHH, copy loss within G3/G4 medulloblastoma and *ZIC1* SNV status revealed that close to 20% of SHH samples harbor genetic variants promoting *ZIC1*/*ZIC4* expression (Fig. 5j). Conversely, approximately 33% of G3 and 60% of G4 samples harbored genetic/epigenetic variants associated with repression of *ZIC1*/*ZIC4* expression (Fig. 5j). These results are consistent with a model in which *ZIC1*, and possibly *ZIC4*, are LOF drivers in G4 medulloblastoma and GOF drivers in SHH medulloblastoma.

### *ZIC1*/*ZIC4* represses G3 medulloblastoma model growth in vitro and in vivo

**Fig. 6:**
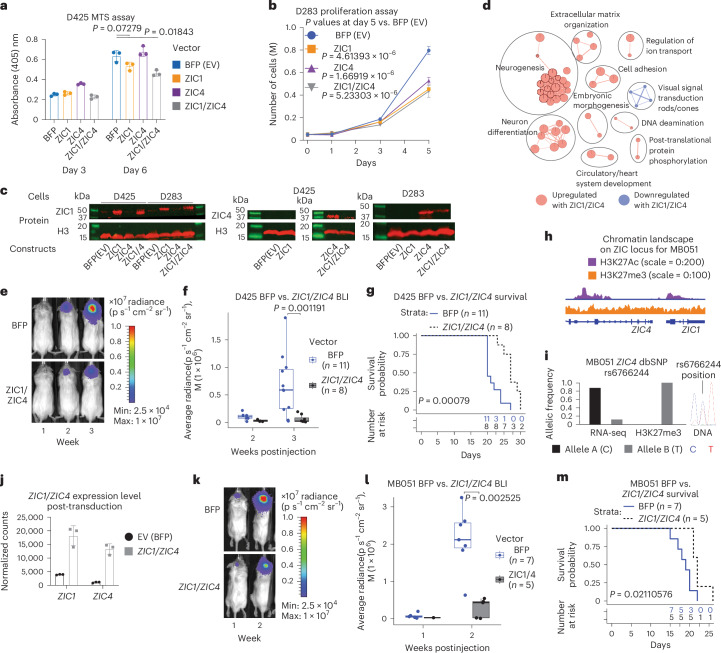
*ZIC1*/*ZIC4* reduces G3 medulloblastoma cell proliferation both in vitro and in vivo*.* **a**, 3-(4,5-dimethylthiazol-2-yl)-5-(3-carboxymethoxyphenyl)-2-(4-sulfophenyl)-2H-tetrazolium (MTS) cell proliferation assay results (mean ± s.d.) for D425. Three biological replicates. *P* values from two-tailed Welch *t*-test. **b**, Cell proliferation assay results for D283. *P* values from two-tailed Welch *t*-test. Data points show mean ± s.d. Five biological replicates. Center of box, median. Bounds of box, 25% and 75% percentile. Whiskers show minimum and maximum values within the 1.5× interquartile range. **c**, Western blot validation of *ZIC1*/*ZIC4* overexpression in D283 and D425. **d**, Pathway analysis for *ZIC1*/*ZIC4* versus EV (BFP) overexpressing D425 (RNA-seq, biological *n* = 3). **e**,**f**. Representative images (**e**) and whisker box plots (**f**) summarizing BLI signals in BFP versus *ZIC1*/*ZIC4* overexpressing D425-injected mice. *P* values were calculated by two-tailed Welch *t*-test. Same whisker box plot parameters as **b**. **g**, Survival curves for BFP versus *ZIC1*/*ZIC4*-transduced D425-injected mice. *P* values from two-tailed log-rank test. **h**, Normalized bigwig tracks showcasing chromatin state of *ZIC1*/*ZIC4* locus in patient-derived G3 xenograft line MB051. **i**, Allelic frequency of heterozygous SNP rs6766244 on coding exon of *ZIC4* from MB051 RNA-seq and H3K27me3 ChIP–seq counts, and Sanger sequencing result from the tumor DNA for the same SNP. **j**, *ZIC1*/*ZIC4*-normalized counts from RNA-seq in MB051 (biological *n* = 3 for EV and *ZIC1*/*ZIC4* constructs). Mean ± s.d. **k**,**l**. Representative images (**k**) and whisker box plots (**l**) summarizing BLI signals in BFP versus *ZIC1*/*ZIC4* overexpressing MB051-injected mice. *P* values were calculated by two-tailed Welch *t*-test. Same whisker box plot parameters as **b**. **m**, Survival curves for BFP versus *ZIC1*/*ZIC4*-transduced MB051-injected mice. *P* values from two-tailed log-rank test. H3, histone 3; EV, empty vector. Source data

**Extended Data Fig. 5:**
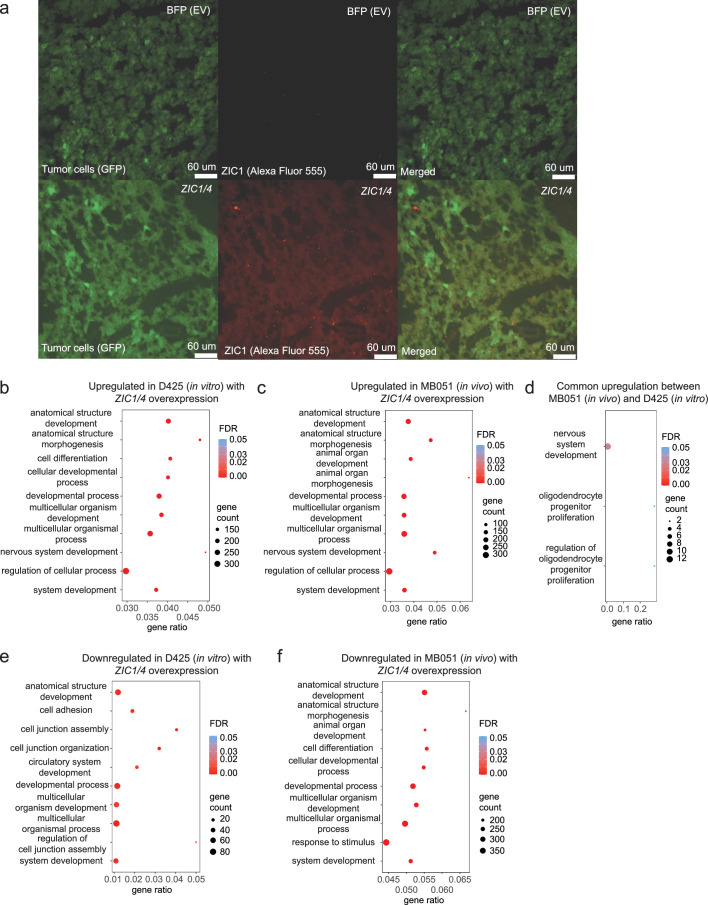
MB051 exhibits similar transcriptional changes as D425 upon ZIC1/4 overexpression in vivo. **a**, Immunofluorescence showing tumor cells (GFP+) and ZIC1 protein level (Alexa Fluor 555), both separately and merged, for BFP (empty vector) or *ZIC1*/4-transduced MB051 patient-derived group 3 (G3) medulloblastoma xenograft intracranially injected into NOD SCID γ (NSG) mice. One biological replicate for BFP-transduced MB051, and two biological replicates for *ZIC1*/4-transduced MB051. Two fields of views captured for BFP, and four fields of views captured for ZIC1/4-transduced MB051 (3 for one biological replicate and 1 for another). All views exhibited identical observations. **b**, Top 10 pathways upregulated in D425 in vitro upon overexpression of *ZIC1*/*4* compared to BFP empty vector. **c**, Top 10 pathways upregulated in MB051 in vivo upon overexpression of *ZIC1*/*4* compared to BFP empty vector. **d**, Pathway analysis depicting commonly upregulated pathways between D425 in vitro and MB051 in vivo. While there was a small overlap, neuronal differentiation pathway emerged as a commonly upregulated pathway between two different models. **e**, Top 10 pathways downregulated in D425 in vitro upon overexpression of *ZIC1*/*4* compared to BFP empty vector. **f**, Top 10 pathways downregulated in MB051 in vivo upon overexpression of *ZIC1*/*4* compared to BFP empty vector.

**Extended Data Fig. 6:**
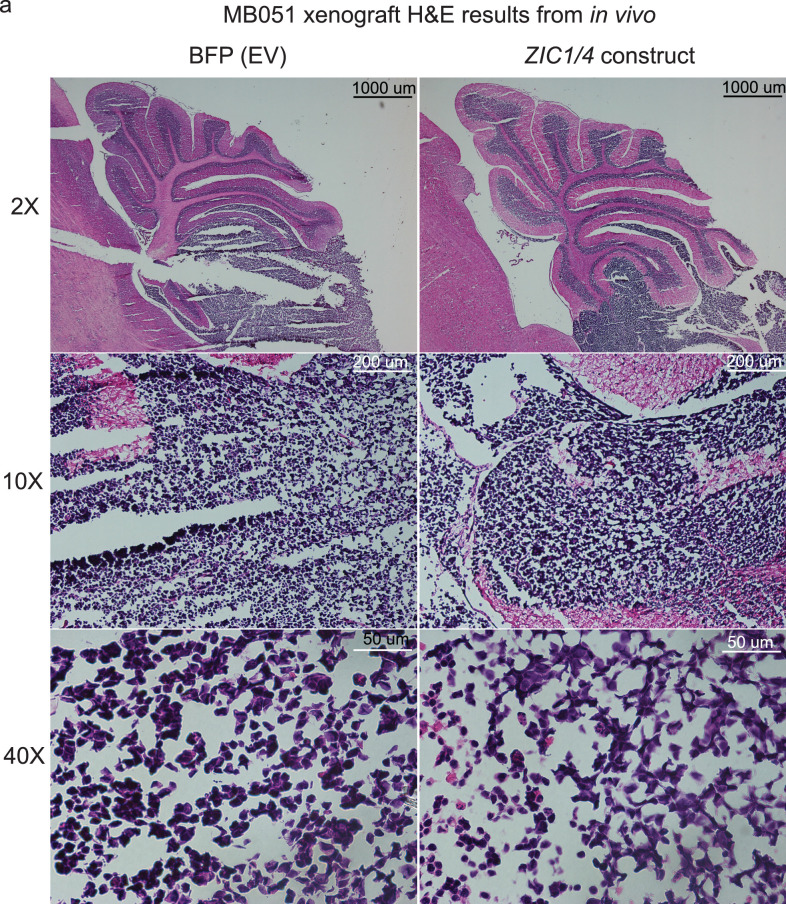
*ZIC1*/*4* overexpression does not result in morphological differences for MB051 at the H&E level. Representative H&E results at various magnifications generated from injecting MB051 into NOD SCID γ (NSG) mice. Magnifications are shown on the left side of the panels. MB051 was transduced with BFP (empty vector) or *ZIC1*/*4* overexpression construct prior to injection. Minimal morphological differences were observable between the different constructs. One biological replicate for BFP-transduced MB051, and two biological replicates for *ZIC1*/*4*-transduced MB051. Three fields of views captured for BFP and each biological replicate of *ZIC1*/*4-*transduced MB051. Twenty-one fields of views for BFP-transduced MB051, 20 fields of views for one replicate of *ZIC1*/4-transduced MB051 and 27 fields of views for the other replicates. Images were captured at varying magnifications ranging from ×2, ×10, and ×40.

Due to the lack of accurate, robust G4 medulloblastoma cell lines, we examined the functional importance of *ZIC1*/*ZIC4* by overexpressing blue fluorescence protein (BFP) empty vector, ZIC1, ZIC4 or ZIC1 and ZIC4 together in D425 and D283 G3 medulloblastoma cell lines. Because G3 and G4 medulloblastomas are (1) molecularly similar and (2) exhibit highly similar genetic and epigenetic dysregulation of the *ZIC1*/*ZIC4* locus, G3 medulloblastoma cell lines were considered relevant for these experiments. Overexpression of ZIC1 led to a significant reduction in the proliferative potential of D425 with evidence for some additive activity with ZIC4 (Fig. 6a). Similar results were observed for D283 in a cell proliferation assay (Fig. 6b,c). Overexpression of ZIC1/ZIC4 in G3 medulloblastoma lines followed by transcriptional profiling revealed increased expression of genes involved in neuronal differentiation, consistent with a model in which LOF of *ZIC1*/*ZIC4* might hinder differentiation (Fig. 6d). Cerebellar xenografting of NOD SCID γ (NSG) mice with D425 cells overexpressing *ZIC1*/*ZIC4* or BFP empty vector demonstrated a significant difference in both bioluminescence imaging (BLI) signal and survival (Fig. 6e–g). The patient-derived G3 xenograft, MB051, harbors single allele chromatin-based suppression of the *ZIC1*/*ZIC4* locus (Fig. 6h,i and Supplementary Table 15). Restoring *ZIC1*/*ZIC4* expression in MB051 significantly reduces BLI signal, as well as prolonging survival in vivo (Fig. 6j–m) in a setting with pre-existing *ZIC1*/*ZIC4* chromatin repression. Upon endpoint, *ZIC1* expression was minimal with the *ZIC1*/*ZIC4* overexpression construct (but higher than an empty vector), suggesting a possible negative selection for cells highly expressing *ZIC1* over time in vivo (Extended Data Fig. 5a). MB051 also exhibited upregulation of neuronal differentiation-associated genes with *ZIC1*/*ZIC4* overexpression in vivo (Extended Data Fig. 5b–f), although morphological changes were not evident (Extended Data Fig. 6). Taken together, our results show tumor suppressive roles of genes in the *ZIC1*/*ZIC4* locus, especially *ZIC1*.

### SHH and G4 medulloblastoma *ZIC1* mutants exert opposite phenotypes

**Fig. 7:**
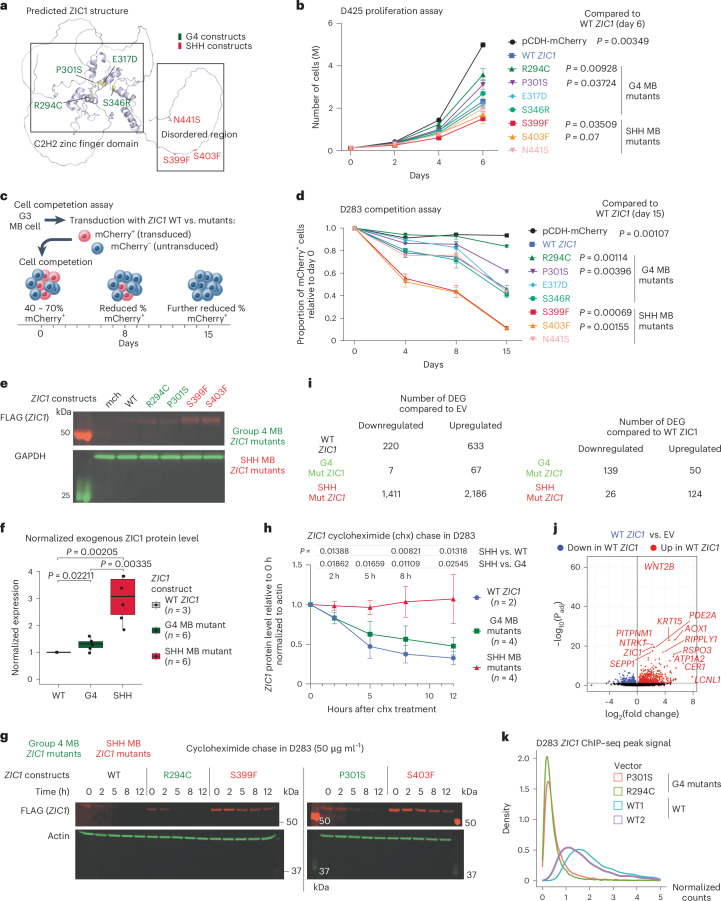
*ZIC1* mutations from G4 and SHH medulloblastoma are functionally distinct. **a**, AlphaFold2 predicted structure of *ZIC1*. Mutant constructs generated and used in the study are summarized in the structure. **b**, Proliferation assay for D425 G3 cell line transduced with *ZIC1* mutant constructs and mCherry EV. Three technical replicates for each construct. Mean ± s.d. *P* values from two-tailed Welch *t*-test. **c**, Schematic representation for the cell competition assay using D283. **d**, Cell competition assay results using D283 transduced with *ZIC1* mutant constructs and mCherry EV. Three technical replicates for each construct. Mean ± s.d. *P* values from two-tailed Welch *t*-test. **e**, Representative western blot visualization of exogenous ZIC1 expression in D283 transduced with FLAG-*ZIC1* constructs. **f**, Whisker box plots showing exogenous ZIC1 expression in D283 transduced with FLAG-*ZIC1* constructs. Signals were normalized by transduction efficiency and *GAPDH* levels. Center of box—median. Bounds of box—25% and 75% percentile. Whiskers show minimum and maximum values within the 1.5× interquartile range. *P* values from two-tailed Welch *t*-test. **g**, Representative cycloheximide chase results for WT and mutant *ZIC1* constructs in D283. **h**, Comparison of ZIC1 protein level across varying exposure times to cycloheximide for WT (*n* = 2), G4 medulloblastoma mutant (*n* = 4) and SHH medulloblastoma *ZIC1* mutant (*n* = 4) constructs. *n*, biological replicates. Mean ± s.d. *P* values from two-tailed Welch *t*-test. **i**, Number of DEG (DESeq2 output) for *ZIC1* constructs when compared against EV or WT *ZIC1*. *Q* value cutoff of 0.05. **j**, Volcano plot summarizing differentially expressed genes between WT *ZIC1* and EV. **k**, Distribution of normalized reads from FLAG ChIP–seq peaks from FLAG-tagged WT versus G4 medulloblastoma mutant *ZIC1*-transduced D283. DEG, differentially expressed genes. Source data

**Extended Data Fig. 7:**
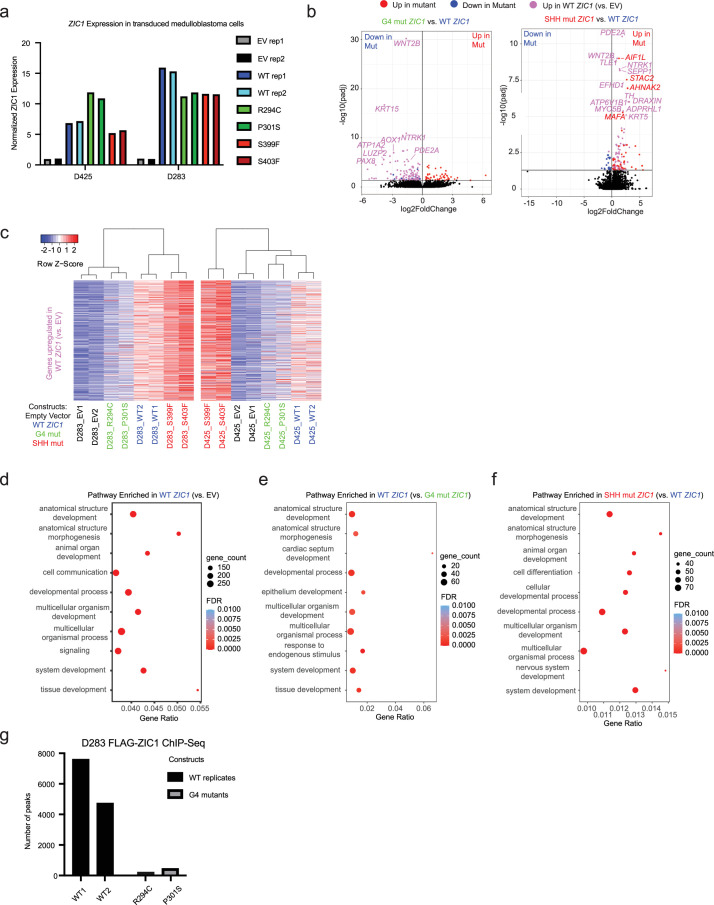
Group 4 (G4) and SHH medulloblastoma *ZIC1* mutant overexpression result in distinct transcriptional changes in group 3 (G3) cells. **a,** *ZIC1* transcript levels (qRT-PCR) across the biological and technical replicates of G3 cell lines transduced with *ZIC1* constructs. Primers used are in Supplementary Table 1. **b**, Volcano plot summarizing genes differentially expressed in G4 medulloblastoma mutant vs. wild-type (WT) *ZIC1* and SHH medulloblastoma mutant vs. WT *ZIC1*-transduced G3 medulloblastoma cells (D425 and D283). Genes that are upregulated with WT *ZIC1* compared to empty vectors are highlighted in purple. *P* adjusted threshold of 0.05 was used. **c**, Heatmap showcasing expression pattern of all WT *ZIC1*-induced genes across all *ZIC1* mutation construct overexpressing cells. G4 medulloblastoma *ZIC1* mutants exhibit reduced upregulation of the *ZIC1* target genes, whereas SHH medulloblastoma *ZIC1* mutants exhibit augmented upregulation of these genes. **d**, Pathway analysis of genes upregulated with WT *ZIC1* construct compared to empty vector. **e**, Pathway analysis of genes that are downregulated with G4 medulloblastoma *ZIC1* mutant compared to WT *ZIC1*. **f**, Pathway analysis of genes upregulated by SHH medulloblastoma *ZIC1* mutant compared to WT *ZIC1*. **g**, Number of ChIP–seq peaks identified from Flag-tagged ZIC1 ChIP–seq in D283 cells transduced with WT *ZIC1* or G4 medulloblastoma *ZIC1* mutant. Two biological replicates were generated for each arm, using different constructs for the G4 medulloblastoma *ZIC1* mutants.

As the CNAs in SHH (gain) and G4 (deletion) are diametrically opposed, we hypothesized that the SHH medulloblastoma SNVs would have divergent biological activity compared to G4 medulloblastoma SNVs, consistent with GOF and LOF phenotypes, respectively. To test this hypothesis, we generated *ZIC1* expression constructs with mutations from G4 medulloblastoma (G4 medulloblastoma *ZIC1* mutants) in the zinc finger regions or with mutations from SHH medulloblastoma (SHH medulloblastoma *ZIC1* mutants) in the carboxy terminus IDR (Fig. 7a). Consistent with our hypothesis, cell proliferation assays in D425 and cell competition assays in D283 demonstrated a reduced antiproliferative effect for the G4 medulloblastoma *ZIC1* mutants compared to the wild-type (WT) *ZIC1*, whereas SHH medulloblastoma *ZIC1* mutants exhibited even more profound growth repression (Fig. 7b–d). We noted marked overexpression after Western blotting for SHH medulloblastoma ZIC1 mutant proteins as compared to WT controls or G4 medulloblastoma ZIC1 mutant proteins (Fig. 7e,f). Cycloheximide pulse-chase assays demonstrated that SHH medulloblastoma ZIC1 mutant proteins exhibit significantly higher protein stability, as compared to WT ZIC1, or G4 medulloblastoma ZIC1 mutant proteins, suggesting that the carboxy terminus IDR exerts control over the stability of the ZIC1 protein (Fig. 7g,h). Overexpression of G4 medulloblastoma *ZIC1* mutant constructs in G3 medulloblastoma cell lines leads to tenfold fewer upregulated genes, as compared to WT *ZIC1*, whereas overexpression of the SHH medulloblastoma *ZIC1* mutant constructs resulted in more differentially expressed genes as compared to WT controls (Fig. 7i,j and Extended Data Fig. 7a–c). WT ZIC1 overexpression led to activation of pathways involved in development and organogenesis, which was dampened with the G4 medulloblastoma *ZIC1* mutants but further augmented with the SHH medulloblastoma *ZIC1* mutants (Extended Data Fig. 7d–f). ChIP–seq against Flag-ZIC1 demonstrates reduced DNA-binding affinity of G4 medulloblastoma ZIC1 mutant proteins, offering a mechanistic insight underlying the reduction of *ZIC1* target gene induction (Fig. 7k and Extended Data Fig. 7g). As the G4 medulloblastoma *ZIC1* point mutations occur in the DNA-binding domain, we conclude therefore that loss of DNA binding is at least partially responsible for the phenotype of G4 medulloblastoma *ZIC1* mutants.

### *ZIC1* is a GOF driver in SHH medulloblastoma

**Fig. 8:**
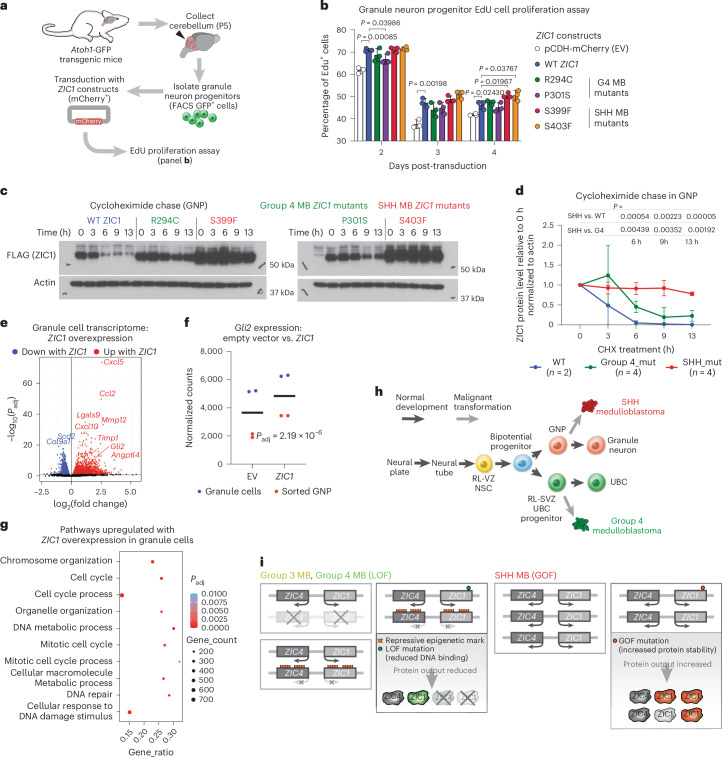
*ZIC1* is a GOF driver oncogene in SHH medulloblastoma. **a**, Schematic representation summarizing GNP 5-ethynyl-2'-deoxyuridine (EdU) proliferation assay. FACS, fluorescence activate cell sorting. **b**, Summary of EdU proliferation assay for GNP transduced with *ZIC1* mutant constructs and mCherry EV. GNPs enriched from multiple mouse cerebellums were used to generate biological triplicates for each construct. Mean ± s.d. as error bars. *P* values were calculated by two-way ANOVA. **c**, Representative results from two independent replicates from running cycloheximide (CHX) chase on GNP transduced with WT *ZIC1* construct, two G4 medulloblastoma *ZIC1* mutant constructs and two SHH medulloblastoma *ZIC1* mutant constructs. **d**, Comparison of ZIC1 protein level from GNP across varying exposure times to cycloheximide for WT (*n* = 2), G4 medulloblastoma mutant (*n* = 4) and SHH medulloblastoma *ZIC1* mutant (*n* = 4) constructs. *n*, biological replicates. Mean ± s.d. *P* values were calculated by two-tailed Welch *t*-test. **e**, RNA-seq-derived volcano plot summarizing DEG (DESeq2 output) between *ZIC1* (mch^+^ *ZIC1*^+^) and EV (mch^+^) transduced granule cells. Two biological replicates were generated for bulk granule cells and sorted GNPs (biological *n* = 4). *Q* value cutoff of 0.05. **f**, Normalized RNA-seq counts for *Gli2* transcript in EV (mch^+^) and *ZIC1* (mch^+^ *ZIC1*^+^) transduced GNPs. Adjusted *P* value from differential expression was calculated from DESeq2 differential expression analysis. **g**, Top ten pathways upregulated with *ZIC1* overexpression in bulk granule cells and GNPs. **h**,**i**, Summary of normal rhombic lip development (**h**) as well as epigenetic and genetic events (**i**) that lead to *ZIC1* LOF in G3 and G4 medulloblastoma and *ZIC1* GOF in SHH medulloblastoma. ANOVA, analysis of variance; NSC, neural stem cell. Source data

**Extended Data Fig. 8:**
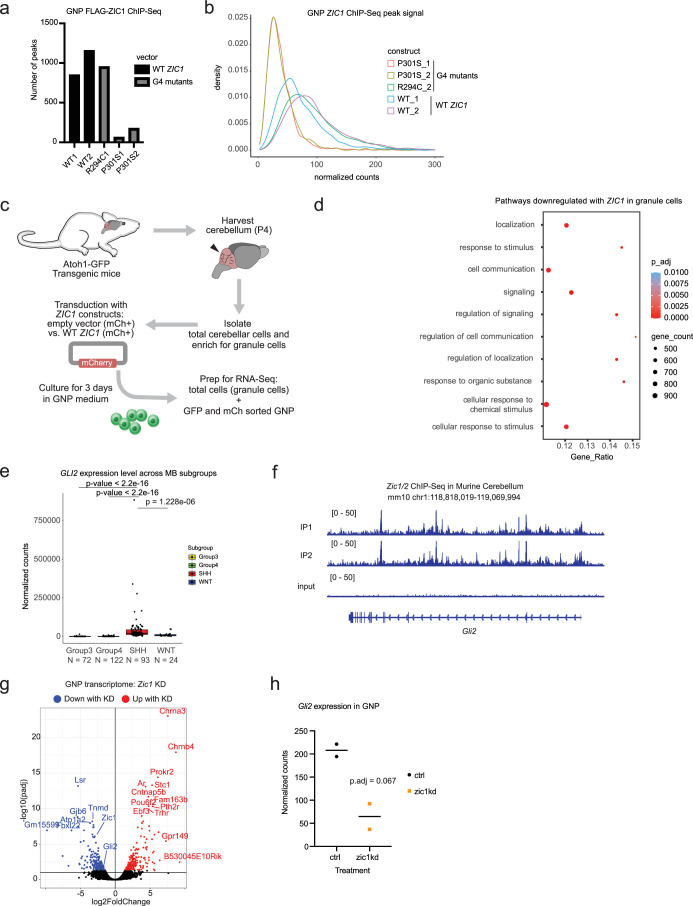
*ZIC1* regulates *Gli2* and cell cycle pathway genes in granule cells. **a**, Number of ChIP–seq peaks identified from Flag-tagged ZIC1 ChIP–seq in granule neuron progenitor (GNP) cells transduced with wild-type (WT) *ZIC1* or group 4 (G4) medulloblastoma *ZIC1* mutant. Two biological replicates were generated for WT *ZIC1* and three for G4 medulloblastoma *ZIC1* mutants. **b**, Distribution of normalized reads for WT vs. G4 medulloblastoma mutant Flag-tagged ZIC1-transduced GNP cells across peaks identified from FLAG ChIP–seq. **c**, Schematic summarizing the RNA-seq libraries generated from mouse granule lineage cells. **d**, Top 10 pathways downregulated by *ZIC1* overexpression compared to empty vector in bulk granule cells and GNPs. **e**, Expression level of *GLI2* across different medulloblastoma molecular subgroups. Plot was generated using the RNA-seq cohort used in the study (N = 311). *GLI2* exhibits a highly SHH medulloblastoma-specific expression pattern. Center of box—median. Bounds of box—25% and 75% percentile. Whiskers show minimum and maximum values within the 1.5× interquartile range. P values calculated by two-tailed Mann–Whitney U test. **f**, *Zic1*/*2* ChIP–seq track demonstrating presence of peaks on the *Gli2* promoter in 2 immunoprecipitation replicates but not in input (data for **f**–**h** from GSE60731). **g**, Volcano plot summarizing genes differentially expressed by knocking down *Zic1* from mouse GNP. *P* adjusted threshold = 0.05. **h**, Normalized counts of *Gli2* transcript in control shRNA and *Zic1* shRNA treated GNP. Biological sample size = 2 for each arm. *P* adjusted value was obtained from DESeq2 differential expression analysis.

**Extended Data Fig. 9:**
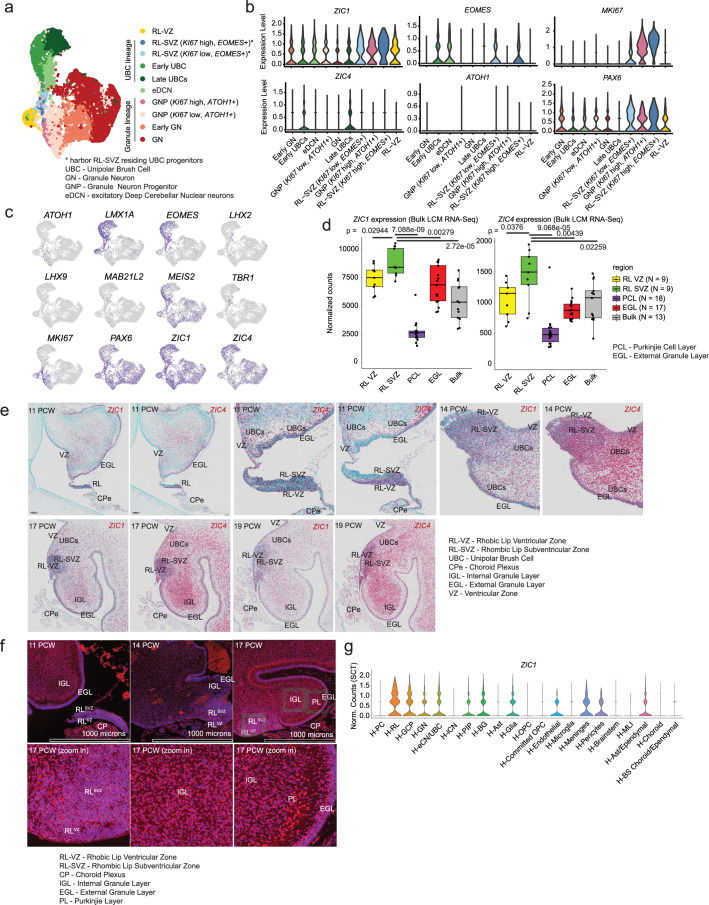
*ZIC1*/*4* are expressed throughout the rhombic lip, particularly in the rhombic lip ventricular zone (RL-VZ) and rhombic lip subventricular zone (RL-SVZ). **a**, Breakdown of glutamatergic neuronal cell lineage from developing human cerebellum (panel **a**–**c** from ref. ^59^ data). RL-SVZ cell populations were further subdivided according to expression pattern of *KI67*, *EOMES* and *ATOH1*. **b**, Violin plots summarizing expression level of *ZIC1*, *ZIC4*, *KI67* and other transcription factors critical for rhombic lip development throughout distinct glutamatergic lineage cell types. **c**, Feature plot summarizing expression levels for 12 developmental transcription factors across the developing human rhombic lip. **d**, Bulk RNA-seq quantification of *ZIC1* and *ZIC4* transcript levels across human rhombic lip regions isolated by laser capture microdissection (LCM; ref. ^37^ data). Center of box—median. Bounds of box—25% and 75% percentile. Whiskers show minimum and maximum values within the 1.5× interquartile range. P values from two-tailed Mann–Whitney U test. **e**, RNA-scope visualization of *ZIC1* and *ZIC4* expression pattern across different regions of the rhombic lip in developing human cerebellum (11–19 postconception weeks). High expression level of both transcripts is observed across all regions, particularly in the RL-VZ and RL-SVZ. Biological sample size of 1 for 11, 14, 17 and 19 post-conception weeks (PCW). **f**, Immunofluorescence result showcasing ZIC1 protein expression pattern across different regions of the rhombic lip in developing human cerebellum (11–17 postconception weeks). Biological sample size of 1 for 11, 14 and 17 PCW. Three different sections were used for each sample. Representative images are shown. **g**, Violin plots summarizing expression level of *ZIC1* transcript across different cells of the developing cerebellum (ref. ^59^ data).

Contrary to *ZIC1* suppressing the growth of G3 medulloblastoma, we hypothesized that *ZIC1* would promote the growth of SHH medulloblastoma. Indeed, overexpression of *ZIC1* constructs in mouse granule neuron progenitor (GNPs) cells (the cell of origin for SHH medulloblastoma)^10,12^ results in increased cellular proliferation, which was more pronounced with the SHH medulloblastoma *ZIC1* mutants as compared to WT ZIC1 or G4 medulloblastoma *ZIC1* mutants (Fig. 8a,b). Cycloheximide chase in GNPs transduced with *ZIC1* mutant constructs revealed that SHH medulloblastoma *ZIC1* mutants also increase protein stability in GNPs, demonstrating the conservation of mutant mechanism across different cell types (Fig. 8c,d). ZIC1 ChIP–seq in GNPs transduced with *ZIC1* mutant constructs also demonstrated reduced DNA-binding affinity for G4 medulloblastoma *ZIC1* mutants similar to results observed in D283 (Extended Data Fig. 8a,b). Transduction of GNPs with *ZIC1* constructs promoted higher expression of cell cycle pathway genes as well as *Gli2*, the main effector of SHH signaling (Fig. 8e–g and Extended Data Fig. 8c,d)^14,26^. *Gli2* is a known oncogene for SHH medulloblastoma, which exhibits a highly SHH medulloblastoma-enriched expression pattern as well as ZIC1-binding motif enrichment in its promoter (Extended Data Fig. 8e and Supplementary Table 16). Re-analysis of published datasets^33^ demonstrates that Zic1 binds the *Gli2* promoter in the mouse cerebellum and that loss of Zic1 is associated with diminished expression of *Gli2* (Extended Data Fig. 8f–h). These data are consistent with a model in which ZIC1 expression represses cell growth in maturing unipolar brush cell (UBC) progenitors of the RL-SVZ (origin of G4 medulloblastoma)^2,3^, whereas it promotes growth of GNPs (origin of SHH medulloblastoma) in the developing cerebellar external granule layer (EGL). In the mouse, after the generation of eomesodermin (EOMES)+ excitatory deep cerebellar nuclear neuron committed cells at E10.5–E12.5 (refs. ^34,35^), the RL-SVZ arises as a bipotent progenitor zone capable of producing both GNPs and UBCs from E13.5 (refs. ^35,36^). Publicly available data on developing human cerebellum^3,37^, as well as newly generated RNA-scope results, demonstrated that both *ZIC1* and *ZIC4* are highly expressed in UBC progenitors of the RL-SVZ (Extended Data Fig. 9a–g). The genetic and chromatin variants of *ZIC1* and *ZIC4* in G4 and SHH medulloblastoma suggest a model in which the activity of ZIC TFs has context-dependent roles in UBC and granule neuron lineage cells, which cumulatively constitute the majority of the neurons in a human brain (Fig. 8h,i).

## Discussion

G3 and G4 medulloblastoma are molecularly distinct medulloblastoma subgroups that are highly related to each other and share many oncogenic drivers^38^. We report similar ZIC1 LOF phenotypes manifesting in G3 and G4 (epigenetic suppression, copy deletion and LOF mutation), albeit at different proportions, suggesting that the *ZIC1*/*ZIC4* locus has similar roles within each subgroup and possibly within their cells of origin. On the other hand, while SHH medulloblastoma shares a direct developmental relationship with G4 medulloblastoma, *ZIC1*/*ZIC4* events confer a GOF phenotype. These findings suggest that *ZIC1*/*ZIC4* has opposing roles in G3/G4 medulloblastoma versus SHH medulloblastoma, raising the possibility that these genes may also have distinct roles in the cells of origin for these similar but distinct tumor types.

Our genetic and experimental data provide robust support for a model in which LOF mutations/chromatin variants in the *ZIC1*/*ZIC4* locus promote G4 medulloblastoma, while GOF mutations promote SHH medulloblastoma within the different lineages of the rhombic lip. *ZIC1* events in the current cohort are found in 20% of SHH medulloblastoma and 60% of G4 medulloblastoma, making *ZIC1* one of the most frequently affected driver genes in medulloblastoma biology. While *ZIC4* is coregulated with *ZIC1* through recurrent epigenetic suppression and copy number changes, the functional role of *ZIC4* in G3 medulloblastoma cell lines is minimal compared to that of *ZIC1*. Furthermore, somatic point mutations have only been identified for *ZIC1* and not for *ZIC4*. As such, we predict that *ZIC1* has a more dominant role in medulloblastoma tumorigenesis, with ZIC4 potentially providing some additive effects.

Our discovery of a H3K27me3/H3K27ac heterozygous chromatin state in G4 medulloblastomas at the *ZIC1*/*ZIC4* locus demonstrates a convincing complementation group in which some tumors achieve repression of *ZIC1* through deletion or somatic mutations of genomic DNA, while other tumors reach the same phenotype through chromatin variants that impose epigenetic repression. This may be through somatic acquisition of chromatin variants, akin to de novo allele-specific ‘epimutations’ that have been described to be associated with oncogenesis^39,40^. Indeed, this robust complementation group provides strong evidence for the biological importance of somatic chromatin variants in the pathogenesis of cancer. We suggest that the observed chromatin events drive the clonal selection of tumor cells and are not merely passenger events.

We were unable to use current technologies to identify local or distal cryptic noncoding mutations driving the H3K27me3/H3K27ac heterozygous chromatin state, although we acknowledge that these may occur and be currently cryptogenic. It is also possible that there exists a minor unidentified population in the rhombic lip that is temporally or anatomically restricted and passes through a state with the H3K27me3/H3K27ac heterozygous chromatin state, and that these particular cells are at increased risk for transforming to G4 medulloblastoma. An additional possible mechanism is somatic ‘epimutation’, in which aberrant H3K27me3 marks repress *ZIC1* expression, and this heritable chromatin state results in clonal expansion and eventually G4 medulloblastoma. The consistent co-occurrence of somatic mutations of histone lysine modifier genes in G4 medulloblastomas that also harbor somatic chromatin variants of *ZIC1* is consistent with a model in which aberrant control of the epigenome leads to ‘epigenetic instability’, with clones that by error contain *ZIC1* silencing chromatin events undergoing clonal selection. Similarly, it has been previously shown that succinate dehydrogenase deficiency can induce aberrant epigenetic remodeling mono-allelically^41^. Which of the three outlined mechanisms, or mechanisms not currently suspected, is responsible for the H3K27me3/H3K27ac heterozygous chromatin state is, however, not currently known, nor readily determined using current technologies, although we favor the somatic chromatin variant model.

G4 medulloblastoma comprises cells similar to the UBC progenitors within RL-SVZ, while SHH medulloblastoma cells resemble GNPs of the EGL. These highly related cell types likely arise from the same bipotential progenitors. The clear difference between the LOF phenotypes (G4) versus GOF phenotypes (SHH) suggests a model in which *ZIC1* and/or *ZIC4* have context-dependent roles in UBC progenitors and GNP during rhombic lip development. In GNPs, *ZIC1*/*ZIC4* may work in conjunction with other SHH pathway genes, such as *GLI2*, to promote cell proliferation and granule-cell-like transcriptome. Tight regulation of ZIC1/ZIC4 activity is likely critical to prevent overexpansion of GNPs during EGL formation. Conversely, UBC progenitors likely require higher levels of ZIC1/ZIC4 activity for normal differentiation, as shown by the UBC lineage-enriched *ZIC1*/*ZIC4* expression pattern. Perturbation of ZIC1/ZIC4 activities in these different contexts likely contributes to improper rhombic lip development and favors oncogenic transformation, where LOF genetic/chromatin variants promote the transformation of the UBC progenitors and GOF variants promote the transformation of the GNPs.

We maintain that LOF/GOF mutations of *ZIC1* are true driver events, as overexpression of *ZIC1* represses malignant phenotypes in G3 medulloblastoma models while promoting malignancy in SHH medulloblastoma precursor cells, both in vitro and in vivo. Indeed, our data support a model in which *ZIC1* is the paramount example of a context-specific cancer driver gene, as it appears to show diametrically opposing biological activity in these two different cell types that arise from the exact same progenitors and which occur on either side of a very specific cell fate decision during rhombic lip development.

## Methods

### Research ethics board (REB)

This study obtained full ethics approval from the Hospital for Sick Children (REB 0020020238 and REB 1000055059) as well as McGill University Health Centre (REB MCH003-26). All materials were collected after receiving written informed consent from patients, including consent to publish the generated data. All primary sample collection and experimental procedures (in vitro and in vivo) were done in accordance with guidelines from the REB of Hospital for Sick Children (REB 0020020238 and REB 1000055059), McGill University Health Centre (REB MCH003-26) and the Centre for Phenogenomics (AUP 22-0151H).

### Experimental model and subject details

#### Primary tumor collection

Primary tumors used in the study were obtained from the Medulloblastoma Advanced Genomics International Consortium and International Cancer Genome Consortium. All materials were collected after receiving written informed consents, including consent to publish the generated data, as per guidelines from REB from the following institutes: Agostino Gemelli University Hospital, Children’s Hospital of Minnesota, Cooperative Human Tissue Network, David Geffen School of Medicine at University of California Los Angeles, Duke University, Emory University, Erasmus University Medical Centre, German Cancer Research Centre (DKFZ), Hospital Cantonal De Geneve, Hospital Infantil de Mexico Federico Gomez, Hospital Sant Joan de Deu, Ludwig Maximilans University, Masaryk University, McGill University, McMaster University, Memorial Sloan Kettering Cancer Centre, Miami Children’s Hospital, Portugese Cancer Institute, Queensland Children’s Tumor Bank, Seattle Children’s Hospital Fred Hunchinson Cancer Research Centre, Seoul National University Children’s Hospital, Stanford University School of Medicine, the Chinese University of Hong Kong, Tohoku University, University of California San Francisco, University Health Network, Universitats Kinderklinik, Universite de Lyon, University of Arkansas, University of Calgary, University of Debrecen Medical and Health Science Centre, University of Pittsburgh, University of Ulsan Asan Medical Centre, University of Warsaw Children’s Memorial Health Institute, Vanderbilt Medical Centre and Wolfson Children’s Hospital. Statistical methods were not used to predetermine the sample size. Age, sex, subgroup and subtype information for used tumors are available in Supplementary Table 1. Primary tumor tissues were snap-frozen in liquid nitrogen and stored at −80 °C until use.

#### Mouse housing and husbandry

All mouse breeding and procedures were performed as approved by the Toronto Centre for Phenogenomics.

### Method details

#### G3 medulloblastoma cell lines and xenograft line

D425 and D283 cell lines were derived at Duke University (Supplementary Table 2) and verified with short tandem repeats before being used for experiments. MB051 patient-derived xenograft line was generated at the Hospital for Sick Children and passaged only by serial intracranial injection in NSG mice without expansion in vitro.

#### Source of NOD-SCID-IL2Rγ null mice

NOD-SCID-IL2Rγ null (NSG) mice were obtained from the Toronto Centre for Phenogenomics in-house breeding colony.

#### Intracranial injection of G3 medulloblastoma tumor cells

Intracranial injection was performed on NSG mice (age range of 6–10 weeks, ~50% males and females for all conditions) using D425 and MB051 xenograft lines as previously described^42^ using slightly modified stereotactic coordinates—2 mm posterior to *λ*, 1 mm lateral and 2 mm deep. In total, 2,000 Green fluorescent protein luciferase-tagged D425 cells transduced with BFP empty vector or ZIC1/ZIC4 vector were injected per mouse. In total, 4,000 GFP luciferase-tagged MB051 cells transduced with BFP empty vector or ZIC1/ZIC4 vector were injected per mouse. Humane endpoint was called independently by staff at the Toronto Centre for Phenogenomics based on physiological conditions exhibited by the injected mice. These staff were blinded from construct information. Mice that did not exhibit any BLI signal above the background (2.5 × 10^4^ p s^−1^ cm^−^^2^ sr^−1^) by the third week after injection were excluded from the cohort.

#### Bioluminescence measurement

Bioluminescence was measured in NSG mice injected with GFP Luciferase-tagged tumor cells as previously described^42^. For D425, measurements were taken on week 1 (6–7 days after injection), week 2 (13–14 days after injection) and week 3 (20 days after injection). For MB051, measurements were taken on week 1 (7 days after injection) and week 2 (14 days after injection).

#### RNA-scope on developing human cerebellum slides

Manufacturer-recommended protocols were used for RNA-scope in situ hybridization (ISH) assays as previously described^37^ using RNA-scope 2.5 High Definition-RED Assay (ACDBio, 322350). Briefly, RNA-scope was performed on mid-sagittal sections of the developing vermis, fixed in 10% formalin for 4 weeks. Manufacturer-recommended protocols (ACDBio/Bio-Techne) were used to assay the following probes: Hs-ZIC4 (525661) and Hs-ZIC1 (542991). All sections were counterstained with hematoxylin or methyl green. Stained slides were imaged using the Nanozoomer Digital Pathology slide scanner (Hamamatsu).

#### *ZIC1* mutant construct generation

WT ZIC1 was cloned into pCDH-mCherry or pCDH-GFP empty lentiviral vector using the In-Fusion Snap Assembly Starter Bundle (Takara). Mutagenesis, or N-terminal FLAG tagging of ZIC1, was also done using the In-Fusion kit.

#### Isolation of cerebellar granule cells or GNPs

Cerebellar cells were isolated from the cerebellum as described previously^43^. Briefly, cerebellum from postnatal day 5 (P5) mice was digested with high glucose Dulbecco’s Phosphate Buffered Saline (DPBS) (Thermo Fisher Scientific) containing 10 U ml^−1^ papain (Worthington), 200 μg ml^−1^ l-cysteine and 250 U ml^−1^ DNase (Sigma) for 30 min. Tissue was triturated to obtain a single-cell suspension and then centrifuged through a 35% and 65% Percoll gradient (Sigma). Cells in the layer between 35% and 65% Percoll were washed once with DPBS containing 0.02% BSA and resuspended in GNP culture medium (neurobasal supplemented with B27 (50×), sodium pyruvate (100×), penicillin–streptomycin (100×) and glutamax (100×)). Granule cells or GNPs were enriched by depleting the adherent cells through two incubations in poly-D-lysine(PDL)-coated plates for 20 min each time. Enriched granule cells and GNPs were cultured with GNP culture medium supplemented with 3 μg ml^−1^ SHH (Peprotech) in PDL-coated plates. For the isolation of pure GNPs, cerebellar cells were isolated from Atoh1-GFP mice at P5 as described above. After washing once with DPBS containing 0.02% BSA, cells were suspended with DPBS containing 5% FBS (Thermo Fisher Scientific). GNPs with strong GFP expression (~40%) were sorted and cultured with the GNP culture medium as described above.

#### 5-ethynyl-2′-deoxyuridine (EdU) assay in GNPs

GNPs isolated from P5 Atoh1-GFP mice, as described above, were infected with control (pCDH-mCherry) or ZIC1 viruses (pCDH-mCherry_ZIC1 WT/mutants) in triplicates. Cells were cultured in a GNP culture medium with SHH in PDL-coated 48-well plates. At each time point, cells were treated with 10 μM 5-ethynyl-2′-deoxyuridine (EdU) for 6 h and then dissociated for EdU staining (Click-iT Plus EdU Pacific Blue Flow Cytometry Assay Kit) and flow cytometry analysis. For data analysis, cells were first gated for mCherry^+^ cells. The percentage of proliferating cells (EdU^+^) was then calculated for each sample.

### Quantification and statistical analysis

#### ChIP–seq data processing

Raw ChIP–seq reads were aligned to hg19 genome assembly using bowtie2 (v2.2.1)^44^. PCR duplicates were removed using Picard MarkDuplicates. Reads with mapping quality lower than 20 were removed. Reads from nonchromosomal contigs, mitochondria or ENCODE blacklist regions were also filtered out before peak calling. H3K27ac peaks were identified using MACS2 (v2.1.1.20160309) with the following code: MACS2 callpeak -t IP_bam_file -f BAMPE -g hs --nomodel -B -q 1e-2 (ref. ^45^). H3K27me3 peaks were identified using the following parameters: MACS2 callpeak -t 27me3_IP_bam_file -c input_bam_file -f BAMPE -g hs --nomodel --broad -B -q 1e-5–broad-cutoff 1e-4. Peaks that could not be identified in at least two primary medulloblastomas were excluded from any further analysis. Library sizes for samples in H3K27ac and H3K27me3 samples were calculated using SAMtools^46^ and average fragment sizes of three different batches of H3K27ac and H3K27me3 were evaluated by deeptools^47^ (v3.1.3). H3K27ac and H3K27me3 peaks in each sample were annotated according to their closest genes and then categorized into different classes based on their distributions over different types of features, for example, promoter, exon, intron and distal intergenic. The distance between peaks and their assigned genes was calculated by using the center of the peak and the transcription start site as coordinates.

For ChIP–seq data from D283 cells transduced with FLAG-tagged *ZIC1* constructs, peaks were called using *Q* value threshold of 1 × 10^−5^. For ChIP–seq data from GNP cells transduced with FLAG-tagged *ZIC1* constructs, peaks were called using a *Q* value threshold of 0.05.

#### SNP inference from ChIP–seq libraries

For samples harboring both H3K27ac and H3K27me3 peaks on the *ZIC1*/*ZIC4* locus, ‘H3K27ac–H3K27me3 hemizygous region’ was defined for each sample with bedtools (v2.27) intersect on the called peaks^48^. From the bivalent region containing the *ZIC1*/*ZIC4* locus, allelic frequencies were calculated for each dbSNP151 annotated heterozygous SNP positions from H3K27ac and H3K27me3 library reads using bedtools multicov. Heterozygous SNPs were identified by first calculating allelic frequency *r* = absolute value of (reference (REF) alternate (ALT) allelic frequency). Afterward, SNPs with *r* ≥ 0.6 in both H3K27Ac and H3K27me3, but biased for different alleles in each, were used to infer heterozygous SNPs (ex, H3K27ac enriched for REF allele and H3K27me3 enriched for ALT allele). Alternatively, SNPs with *r* < 0.6 in either H3K27ac or H3K27me3 libraries were also used to identify SNPs. Only SNPs that are supported by at least ten reads from each library were used.

#### SEs analysis and subgroup consensus peak sets

SEs were defined using the Rank Ordering of Super Enhancers (v0.1) algorithm using H3K27ac peaks as input^49^. For all samples, the stitching distance was fixed at 12.5 kb to facilitate comparisons between samples. All other parameters used the default setting. Once SEs were generated for each sample, SEs were merged from samples within the same subgroup using GenomicRanges Bioconductor package^50^. Only SEs that were present at least two times per subgroup were considered for merging.

#### RNA-seq data processing

Custom hs37d5 genome assembly generated in previous study^27^ was used to align raw RNA-seq reads using STAR aligner (2.7.4) with the following parameters: *-*-outFilterMultimapNmax 20 --alignSJoverhangMin 8 --alignMatesGapMax 200000 --alignIntronMax 200000 --alignSJDBoverhangMin 10 --alignSJstitchMismatchNmax 5 -1 5 5 --outSAMmultNmax 20 --twopassMode Basic^51^. Gene expression level was quantified using HTSeq (0.6.0) based on Gencode v19 annotations with the argument ‘-stranded reverse -m union’^52^. Differential gene expression analysis between subgroups was performed using the R Bioconductor package DESeq2 (v1.26.0)^53^. An adjusted *P* value of 0.05 was used for differentially expressed gene identifications.

#### H3K27ac HiChIP data process and loop call

Raw HiChIP reads were aligned using bowtie2 (2.3.4) and HiC-pro (2.9.0) using the default parameters in HiC-pro^54^. Output directory was used as input for hichipper (v0.7.3) to call significant loops using the following parameters: min-dist 5000, max-dist 20000000, read-length 150, ‘macs2-string -q 0.01 --extsize 315 –nomodel’^55^. Intrachromosomal loops with *Q* value less than 0.01 and read counts greater than 5 were used for downstream enhancer gene interactome analysis.

#### WGS data processing and germline variants calling

WGS data were aligned to the ‘hs37d5’ reference genome from 1000 Genomes Project Phase II as previously described^28^, using Burrows–Wheeler aligner–MEM (v0.7.8) with the ‘-T 0’ parameter^56^. For germline variant call, variants identified in both normal and tumor DNA from Platypus (v0.8.1) run with default parameters were used (https://github.com/andyrimmer/Platypus). To have the final heterozygous SNP list for each sample in WGS data, we only selected those passed Platypus quality control (minBaseQual and minMapQual: 20; alleleBias and strandBias: 0.001 and badReadsWindow: 11). Second, we retained SNPs with allele depth in tumor samples ≥10, allele depth in paired blood samples ≥7, allele ratio in blood between (0.3, 0.7) and allele ratio in tumor between (0.2, 0.8). Third, only bi-allelic sites and InDels shorter than three nucleotides were used. The final heterozygous SNP candidates were retained in the following allele imbalance analysis. We used EAGLE2 for haplotype phase estimation on bcftools (v1.9)^57^ normalized variants, using a phased reference panel in 1000 Genomes Project^58^.

#### Affymetrix SNP6 array data processing

SNP6 Affymetrix array data were mapped to hg19 and processed using Affymetrix Power Tools (v1.18.2) as previously described^27^.

#### Identification of focal recurrent CNAs from SNP6 array

To identify recurrent focal copy gains and losses for each subgroup, SNP6 array-derived segmentation files were used as input for GISTIC2 (v2.0.23) from gene pattern with the following options: refgene file = Human_Hg19.mat, maxspace = 10,000, gene gistic = yes, confidence = 0.90, *Q* value threshold = 0.25, run broad analysis = no, max sample segs = 10,000, arm peel = yes, gene collapse method = extreme, amplification threshold = 0.5, deletion threshold = −0.5, focal length cutoff = 0.5, armlevelpeel = on, confidence level = 0.95, *Q* value = 0.25, run broad analysis = no, max sample segs = 10,000 (ref. ^31^). Other parameters were left as default.

#### Single-cell RNA-seq (scRNA-seq) data analysis

Publicly available scRNA-seq data were analyzed as previously described with minor modifications^3,59^. Specifically, RL-SVZ cells from the glutamatergic lineage cells were further divided into three smaller cell clusters using the following criteria: RL-SVZ (KI67 high, EOMES+)—RL-SVZ residing UBC progenitor cells; RL-SVZ (KI67 high, ATOH1+)—RL-SVZ cells more committed to GCP lineage; RL-SVZ (KI67 low, EOMES+)—RL-SVZ residing UBC progenitor cells likely mixed with some early UBC.

#### Pathway enrichment analysis

Enriched pathways for differentially expressed genes were identified by using g-profiler at default parameters, using *Q* value threshold of 0.05 (ref. ^60^). Gene Ontology-biological term outputs were used for the final list of pathways. Top ten enriched/depleted pathways were identified for ZIC1 mutant construct experiments using G3 medulloblastoma cell lines or GNP cells in vitro and G3 medulloblastoma xenograft experiments in vivo.

#### Calling CNA events from WGS data

Copy number information was derived from WGS data using Control-FREEC (v10.3)^32^ as previously described with the following parameters: breakPointType = 4, ploidy = ‘2,3,4’, step = 10,000, window = 50,000 (ref. ^28^).

Before focal CNA call from WGS data for known medulloblastoma driver genes, ploidy for all WGS samples was predicted with Control-FREEC. For samples with inferred ploidy greater than 3.5, pileup ratio was used from ploidy = 4 output. All other samples used pileup ratio from ploidy = 2 output. Median ratio values for each segmented genomic locus were used to generate a segmented (.seg) format for each sample. Merged seg file for each subgroup was used as input for GISTIC2 (v2.0.23) from gene pattern with the following options: refgene file = human_Hg19.mat, maxspace = 10,000, gene gistic = yes, confidence = 0.90, *Q* value threshold = 0.25, run broad analysis = no, max sample segs = 10,000, arm peel = yes, gene collapse method = extreme, amplification threshold = 0.25, deletion threshold = −0.25, focal length cutoff = 0.5, armlevelpeel = on, confidence level = 0.95, *Q* value = 0.25, run broad analysis = no, max sample segs = 10,000 (ref. ^31^). Other parameters were left as default. Output from focal_data_by_genes was used for genes previously identified to undergo recurrent CNA gain in G3/G4—*MYC*, *MYCN*, *OTX2* and *CDK6*, which have been previously reported^14,26^.

For CNA identification from WGS data for the *ZIC1*/*ZIC4* locus, both broad chromosomal events and focal CNA were identified using the seg files generated above. An amplification threshold of 0.25 and a copy loss threshold of −0.25 were used to estimate the proportion of samples with copy number changes in SHH or G3/G4 samples, respectively.

#### Oncoplot generation

Highly expressed genes were identified by performing *k*-means clustering on size factor normalized RNA-seq counts with *k* = 2 for the following genes: *GFI1*, *GFI1B* and *PRDM6*. Group with higher expression of genes were categorized as highly expressing. Somatic SNVs, InDels, CNA amplifications and high expression samples for each gene were annotated for all samples using complexheatmap (v2.2.0) R package^61^.

### Statistics and reproducibility

No statistical method was used to predetermine the sample size. Randomizing and blinding were not used for the experiments. For experiments involving the injection of mice with medulloblastoma cell lines or patient-derived xenograft lines, independent staff at the Toronto Centre for Phenogenomics were blinded from the experimental arm conditions before calling the endpoints. For mouse BLI experiments, mice that failed to reach the minimal detectable signal of 2.5 × 10^4^ p s^−1^ cm^−^^2^ sr^−1^ by the third week postinjection were removed from the cohort (failure to engraft).

### Reporting summary

Further information on research design is available in the Nature Portfolio Reporting Summary linked to this article.

## Online content

Any methods, additional references, Nature Portfolio reporting summaries, source data, extended data, supplementary information, acknowledgements, peer review information; details of author contributions and competing interests; and statements of data and code availability are available at 10.1038/s41588-024-02014-z.

## Supplementary information

Supplementary Information Supplementary Note (full materials and methods).

Reporting Summary

Supplementary Tables 1–16 Supplementary Table 1: Metadata for the samples used in the study. Supplementary Table 2: Reagents and resources used for the study (reagents and resources). Supplementary Tables 3–10: Recurrent CNA genomic loci for each medulloblastoma subgroup determined by using GISTIC on SNP6 array-derived segmentation files (G3_GISTIC_amp, G3_GISTIC_del, G4_GISTIC_amp, G4_GISTIC_del, SHH_GISTIC_amp, SHH_GISTIC_del, WNT_GISTIC_amp, WNT_GISTIC_del). Supplementary Tables 11–13: Subgroup-enriched and recurrent H3K27me3 regions determined from ChIP–seq data, as well as overlapping genes with loci from 2 (subgroup_enriched_27me3_peaks, subgroup_recurrent_27me3_peaks and 27me3_GISTIC_overlap_genes). Supplementary Table 14: Genes used to calculate differentiation score for G3 and G4 medulloblastoma tumors (neuron-like-gene-list). Supplementary Table 15: Primers used in the study (primers). Supplementary Table 16: Summary of transcription factors that exhibit subgroup-enriched expression as well as harbor ZIC1-binding motifs in their promoter (ZIC1_target_TFs_subgroup).

## Source data

Source Data Fig. 6 Data point values for cell proliferation, NSG mouse BLI and survival figures.

Source Data Fig. 6 Uncropped and unprocessed gel images.

Source Data Fig. 7 Uncropped and unprocessed gel images.

Source Data Fig. 7 Data point values for cell proliferation, NSG mouse BLI and survival figures.

Source Data Fig. 8 Uncropped and unprocessed gel images.

Source Data Fig. 8 Data point values for cell proliferation, NSG mouse BLI and survival figures.

## Data Availability

The FLAG ChIP–seq, RNA-seq data generated from *ZIC1* mutant construct transduced G3 medulloblastoma cell lines and granule cells have been deposited in the Gene Expression Omnibus (GEO) database under the accessions GSE217639, GSE217571 and GSE217638. Bulk H3K27ac, H3K27me3 ChIP–seq, RNA-seq, WGS and H3K27ac hichip data generated from primary medulloblastoma tumor samples in this study have been deposited in the European Genome–Phenome Archive (EGA) database under the accession code EGAS00001006741. The published medulloblastoma bulk RNA-seq data referenced in this study are available in the EGA database under the accessions EGAS00001001953, EGAD00001004347, EGAD00001004435, EGAS00001005826, EGAD00001001899, EGAD00001004958 and EGAD00001008458. The published medulloblastoma WGS data referenced in this study are available in the EGA database under the accessions EGAS00001001953, EGAD00001003125 and EGAD00001004347. The published medulloblastoma H3K27ac ChIP–seq data referenced in this study are available in the EGA database under the accessions EGAS00001001953. The Affymetrix SNP 6.0 data referenced during the study are available in the GEO database under the accession GSE37385. The expression array used for transcript abundance comparison between medulloblastoma subtypes is available in the GEO database under the accession GSE132269. Multiple databases were used for annotation of SNPs and promoters, which were referenced in this study. These include the GRCh37 dbSNP151 (https://ftp.ncbi.nlm.nih.gov/snp/organisms/human_9606_b151_GRCh37p13/VCF/), GENCODE (v.19; https://www.gencodegenes.org/human/release_19.html), the hg19 reference genome (https://hgdownload.soe.ucsc.edu/goldenPath/hg19/bigZips/), the hs37d5 reference genome (https://ftp-trace.ncbi.nih.gov/1000genomes/ftp/technical/reference/phase2_reference_assembly_sequence/), ERCC spike-in sequence (https://www.encodeproject.org/files/ENCFF908UQN/) and Caltech profile 3 spike-in sequence (https://www.encodeproject.org/references/ENCSR193ZXE/). snRNA-seq data from the developing human cerebellum were obtained through correspondence from ref. ^59^ and are available through the Human Cell Atlas (https://explore.data.humancellatlas.org/projects/85a9263b-0887-48ed-ab1a-ddfa773727b6), the UCSC Cell Browser (https://cbl-dev.cells.ucsc.edu) or from Database of Genotypes and Phenotypes (dbGaP; accession phs001908.v2.p1). Bulk RNA-seq data from the developing human cerebellum were obtained through correspondence from ref. ^37^ and are available through the dbGaP (accession phs001908.v2.p1). Source data are provided with this paper.Human material provided by the Joint MRC/Wellcome (MR/R006237/1) Human Developmental Biology Resource (HDBR; www.hdbr.org) and the Birth Defects Research Laboratory (BDRL; NIH-R24-HD000836 to I.A.G.) was covered by a material transfer agreement between SCRI and HDBR/BDRL, but samples may be requested directly from the HDBR and BDRL. Please see the Supplementary Information for full lists of the reagents, resources and bioinformatics tools used for the study (Supplementary Tables 1–16). Requests for additional information or resources and reagents should be directed to and will be fulfilled by M.D.T.

## Code availability

Original codes used for the study are available at 10.5281/zenodo.13940242 (ref. ^62^). Full details of methods used for the study can be found in Supplementary Note.

## References

[1] Gibson, P. et al. Subtypes of medulloblastoma have distinct developmental origins. *Nature* **468**, 1095–1099 (2010). 21150899 10.1038/nature09587 PMC3059767

[2] Smith, K. S. et al. Unified rhombic lip origins of group 3 and group 4 medulloblastoma. *Nature* **609**, 1012–1020 (2022). 36131015 10.1038/s41586-022-05208-9 PMC9748853

[3] Hendrikse, L. D. et al. Failure of human rhombic lip differentiation underlies medulloblastoma formation. *Nature* **609**, 1021–1028 (2022). 36131014 10.1038/s41586-022-05215-w PMC10026724

[4] Vladoiu, M. C. et al. Childhood cerebellar tumours mirror conserved fetal transcriptional programs. *Nature* **572**, 67–73 (2019). 31043743 10.1038/s41586-019-1158-7 PMC6675628

[5] Cho, Y. J. et al. Integrative genomic analysis of medulloblastoma identifies a molecular subgroup that drives poor clinical outcome. *J. Clin. Oncol.* **29**, 1424–1430 (2011). 21098324 10.1200/JCO.2010.28.5148 PMC3082983

[6] Kool, M. et al. Integrated genomics identifies five medulloblastoma subtypes with distinct genetic profiles, pathway signatures and clinicopathological features. *PLoS ONE* **3**, e3088 (2008). 18769486 10.1371/journal.pone.0003088 PMC2518524

[7] Northcott, P. A. et al. Medulloblastoma comprises four distinct molecular variants. *J. Clin. Oncol.* **29**, 1408–1414 (2011). 20823417 10.1200/JCO.2009.27.4324 PMC4874239

[8] Taylor, M. D. et al. Molecular subgroups of medulloblastoma: the current consensus. *Acta Neuropathol.* **123**, 465–472 (2012). 22134537 10.1007/s00401-011-0922-z PMC3306779

[9] Thompson, M. C. et al. Genomics identifies medulloblastoma subgroups that are enriched for specific genetic alterations. *J. Clin. Oncol.* **24**, 1924–1931 (2006). 16567768 10.1200/JCO.2005.04.4974

[10] Wechsler-Reya, R. J. & Scott, M. P. Control of neuronal precursor proliferation in the cerebellum by sonic hedgehog. *Neuron* **22**, 103–114 (1999). 10027293 10.1016/s0896-6273(00)80682-0

[11] Wallace, V. A. Purkinje-cell-derived sonic hedgehog regulates granule neuron precursor cell proliferation in the developing mouse cerebellum. *Curr. Biol.* **9**, 445–448 (1999). 10226030 10.1016/s0960-9822(99)80195-x

[12] Yang, Z.-J. et al. Medulloblastoma can be initiated by deletion of patched in lineage-restricted progenitors or stem cells. *Cancer Cell* **14**, 135–145 (2008). 18691548 10.1016/j.ccr.2008.07.003 PMC2538687

[13] Oliver, T. G. et al. Loss of patched and disruption of granule cell development in a pre-neoplastic stage of medulloblastoma. *Development* **132**, 2425–2439 (2005). 15843415 10.1242/dev.01793

[14] Northcott, P. A. et al. The whole-genome landscape of medulloblastoma subtypes. *Nature* **547**, 311–317 (2017). 28726821 10.1038/nature22973 PMC5905700

[15] Aruga, J. The role of Zic genes in neural development. *Mol. Cell. Neurosci.* **26**, 205–221 (2004). 15207846 10.1016/j.mcn.2004.01.004

[16] Grinberg, I. et al. Heterozygous deletion of the linked genes *ZIC1* and *ZIC4* is involved in Dandy–Walker malformation. *Nat. Genet.* **36**, 1053–1055 (2004). 15338008 10.1038/ng1420

[17] Merzdorf, C. S. Emerging roles forzic genes in early development. *Dev. Dyn.* **236**, 922–940 (2007). 17330889 10.1002/dvdy.21098

[18] Mizugishi, K., Aruga, J., Nakata, K. & Mikoshiba, K. Molecular properties of Zic proteins as transcriptional regulators and their relationship to GLI proteins. *J. Biol. Chem.* **276**, 2180–2188 (2001). 11053430 10.1074/jbc.M004430200

[19] Brown, S. A. et al. Holoprosencephaly due to mutations in *ZIC2*, a homologue of *Drosophila* odd-paired. *Nat. Genet.* **20**, 180–183 (1998). 9771712 10.1038/2484

[20] Lin, C. Y. et al. Active medulloblastoma enhancers reveal subgroup-specific cellular origins. *Nature* **530**, 57–62 (2016). 26814967 10.1038/nature16546 PMC5168934

[21] Blank, M. C. et al. Multiple developmental programs are altered by loss of *Zic1* and *Zic4* to cause Dandy–Walker malformation cerebellar pathogenesis. *Development* **138**, 1207–1216 (2011). 21307096 10.1242/dev.054114 PMC3042874

[22] Grinberg, I. & Millen, K. The ZIC gene family in development and disease. *Clin. Genet.* **67**, 290–296 (2005). 15733262 10.1111/j.1399-0004.2005.00418.x

[23] Twigg, S. R. F. et al. Gain-of-function mutations in *ZIC1* are associated with coronal craniosynostosis and learning disability. *Am. J. Hum. Genet.* **97**, 378–388 (2015). 26340333 10.1016/j.ajhg.2015.07.007 PMC4564895

[24] Bahl, S., Carroll, J. S. & Lupien, M. Chromatin variants reveal the genetic determinants of oncogenesis in breast cancer. *Cold Spring Harb. Perspect. Med.* **12**, a041322 (2022). 36041880 10.1101/cshperspect.a041322 PMC9524388

[25] Grillo, G. & Lupien, M. Cancer-associated chromatin variants uncover the oncogenic role of transposable elements. *Curr. Opin. Genet. Dev.* **74**, 101911 (2022). 35487182 10.1016/j.gde.2022.101911

[26] Northcott, P. A. et al. Subgroup-specific structural variation across 1,000 medulloblastoma genomes. *Nature* **488**, 49–56 (2012). 22832581 10.1038/nature11327 PMC3683624

[27] Skowron, P. et al. The transcriptional landscape of Shh medulloblastoma. *Nat. Commun.* **12**, 1749 (2021). 33741928 10.1038/s41467-021-21883-0 PMC7979819

[28] Suzuki, H. et al. Recurrent noncoding U1 snRNA mutations drive cryptic splicing in SHH medulloblastoma. *Nature* **574**, 707–711 (2019). 31664194 10.1038/s41586-019-1650-0 PMC7141958

[29] Aruga, J. & Millen, K. J. ZIC1 function in normal cerebellar development and human developmental pathology. *Adv. Exp. Med. Biol.* **1046**, 249–268 (2018). 29442326 10.1007/978-981-10-7311-3_13

[30] Cavalli, F. M. G. et al. Intertumoral heterogeneity within medulloblastoma subgroups. *Cancer Cell* **31**, 737–754 (2017). 28609654 10.1016/j.ccell.2017.05.005 PMC6163053

[31] Mermel, C. H. et al. GISTIC2.0 facilitates sensitive and confident localization of the targets of focal somatic copy-number alteration in human cancers. *Genome Biol.* **12**, R41 (2011). 21527027 10.1186/gb-2011-12-4-r41 PMC3218867

[32] Boeva, V. et al. Control-FREEC: a tool for assessing copy number and allelic content using next-generation sequencing data. *Bioinformatics* **28**, 423–425 (2012). 22155870 10.1093/bioinformatics/btr670 PMC3268243

[33] Frank, C. L. et al. Regulation of chromatin accessibility and Zic binding at enhancers in the developing cerebellum. *Nat. Neurosci.* **18**, 647–656 (2015). 25849986 10.1038/nn.3995 PMC4414887

[34] Wilson, L. J. & Wingate, R. J. T. Temporal identity transition in the avian cerebellar rhombic lip. *Dev. Biol.* **297**, 508–521 (2006). 16806151 10.1016/j.ydbio.2006.05.028

[35] Leto, K. et al. Consensus paper: cerebellar development. *Cerebellum* **15**, 789–828 (2016). 26439486 10.1007/s12311-015-0724-2 PMC4846577

[36] Englund, C. et al. Unipolar brush cells of the cerebellum are produced in the rhombic lip and migrate through developing white matter. *J. Neurosci.* **26**, 9184–9195 (2006). 16957075 10.1523/JNEUROSCI.1610-06.2006 PMC6674506

[37] Haldipur, P. et al. Spatiotemporal expansion of primary progenitor zones in the developing human cerebellum. *Science* **366**, 454–460 (2019). 31624095 10.1126/science.aax7526 PMC6897295

[38] Hovestadt, V. et al. Resolving medulloblastoma cellular architecture by single-cell genomics. *Nature* **572**, 74–79 (2019). 31341285 10.1038/s41586-019-1434-6 PMC6754173

[39] Shen, H. & Laird, P. W. Interplay between the cancer genome and epigenome. *Cell* **153**, 38–55 (2013). 23540689 10.1016/j.cell.2013.03.008 PMC3648790

[40] Hitchins, M. P. Constitutional epimutation as a mechanism for cancer causality and heritability? *Nat. Rev. Cancer* **15**, 625–634 (2015). 26383139 10.1038/nrc4001

[41] Flavahan, W. A. et al. Altered chromosomal topology drives oncogenic programs in SDH-deficient GISTs. *Nature* **575**, 229–233 (2019). 31666694 10.1038/s41586-019-1668-3 PMC6913936

[42] Garzia, L. et al. A hematogenous route for medulloblastoma leptomeningeal metastases. *Cell* **172**, 1050–1062 (2018). 29474906 10.1016/j.cell.2018.01.038 PMC6346737

[43] Tao, R. et al. *MYC* drives group 3 medulloblastoma through transformation of Sox2^+^ astrocyte progenitor cells. *Cancer Res.* **79**, 1967–1980 (2019). 30862721 10.1158/0008-5472.CAN-18-1787 PMC6467710

[44] Langmead, B. & Salzberg, S. L. Fast gapped-read alignment with Bowtie 2. *Nat. Methods* **9**, 357–359 (2012). 22388286 10.1038/nmeth.1923 PMC3322381

[45] Zhang, Y. et al. Model-based analysis of ChIP–seq (MACS). *Genome Biol.* **9**, R137 (2008). 18798982 10.1186/gb-2008-9-9-r137 PMC2592715

[46] Li, H. et al. The sequence alignment/map format and SAMtools. *Bioinformatics* **25**, 2078–2079 (2009). 19505943 10.1093/bioinformatics/btp352 PMC2723002

[47] Ramírez, F. et al. deepTools2: a next generation web server for deep-sequencing data analysis. *Nucleic Acids Res.* **44**, W160–W165 (2016). 27079975 10.1093/nar/gkw257 PMC4987876

[48] Quinlan, A. R. & Hall, I. M. BEDTools: a flexible suite of utilities for comparing genomic features. *Bioinformatics* **26**, 841–842 (2010). 20110278 10.1093/bioinformatics/btq033 PMC2832824

[49] Whyte, W. A. et al. Master transcription factors and mediator establish super-enhancers at key cell identity genes. *Cell* **153**, 307–319 (2013). 23582322 10.1016/j.cell.2013.03.035 PMC3653129

[50] Lawrence, M. et al. Software for computing and annotating genomic ranges. *PLoS Comput. Biol.* **9**, e1003118 (2013). 23950696 10.1371/journal.pcbi.1003118 PMC3738458

[51] Dobin, A. et al. STAR: ultrafast universal RNA-seq aligner. *Bioinformatics* **29**, 15–21 (2013). 23104886 10.1093/bioinformatics/bts635 PMC3530905

[52] Anders, S., Pyl, P. T. & Huber, W. HTSeq-A Python framework to work with high-throughput sequencing data. *Bioinformatics* **31**, 166–169 (2015). 25260700 10.1093/bioinformatics/btu638 PMC4287950

[53] Love, M. I., Huber, W. & Anders, S. Moderated estimation of fold change and dispersion for RNA-seq data with DESeq2. *Genome Biol.* **15**, 550 (2014). 25516281 10.1186/s13059-014-0550-8 PMC4302049

[54] Servant, N. et al. HiC-Pro: an optimized and flexible pipeline for Hi-C data processing. *Genome Biol.* **16**, 259 (2015). 26619908 10.1186/s13059-015-0831-x PMC4665391

[55] Lareau, C. A. & Aryee, M. J. Hichipper: a preprocessing pipeline for calling DNA loops from HiChIP data. *Nat. Methods* **15**, 155–156 (2018). 29489746 10.1038/nmeth.4583 PMC10572103

[56] Li, H. & Durbin, R. Fast and accurate short read alignment with Burrows–Wheeler transform. *Bioinformatics* **25**, 1754–1760 (2009). 19451168 10.1093/bioinformatics/btp324 PMC2705234

[57] Danecek, P. et al. Twelve years of SAMtools and BCFtools. *GigaScience* **10**, giab008 (2021). 33590861 10.1093/gigascience/giab008 PMC7931819

[58] Loh, P.-R. et al. Reference-based phasing using the Haplotype Reference Consortium panel. *Nat. Genet.* **48**, 1443–1448 (2016). 27694958 10.1038/ng.3679 PMC5096458

[59] Aldinger, K. A. et al. Spatial and cell type transcriptional landscape of human cerebellar development. *Nat. Neurosci.* **24**, 1163–1175 (2021). 34140698 10.1038/s41593-021-00872-y PMC8338761

[60] Reimand, J., Kull, M., Peterson, H., Hansen, J. & Vilo, J. g:Profiler—a web-based toolset for functional profiling of gene lists from large-scale experiments. *Nucleic Acids Res.* **35**, W193–W200 (2007). 17478515 10.1093/nar/gkm226 PMC1933153

[61] Gu, Z., Eils, R. & Schlesner, M. Complex heatmaps reveal patterns and correlations in multidimensional genomic data. *Bioinformatics* **32**, 2847–2849 (2016). 27207943 10.1093/bioinformatics/btw313

[62] Lee, J. J. Y. et al. *ZIC1* is a context-dependent medulloblastoma driver in the rhombic lip. Custom scripts v1.0. *Zenodo*10.5281/zenodo.13940242 (2024).

