# Supplementary material for: ZIC1 is a context-dependent medulloblastoma driver in the rhombic lip
Source: Nat Genet. 2025 Jan 3;57(1):88–102. doi: 10.1038/s41588-024-02014-z (PMC11735403; doi:10.1038/s41588-024-02014-z)
Supplement: Supplementary file 1 — Supplementary Note (full materials and methods). [file 41588_2024_2014_MOESM1_ESM.pdf]

nature genetics

Supplementary information

https://doi.org/10.1038/s41588-024-02014-z

ZIC1 is a context-dependent medulloblastoma driver in the rhombic lip

In the format provided by the authors and unedited

Supplementary Information

Full materials and methods:

Crosslinking primary tumor tissues

Primary frozen tumors were pulverized with mortar, pestle and liquid nitrogen as previously described[1]. Briefly, pulverized tissue pieces were crosslinked for 10 minutes in 1% formaldehyde (Sigma, F8775), quenched with glycine (0.125M final concentration), immediately spun down (2500g, 5 min), washed once with PBS and kept at -80C until downstream use.

RNA Seq on primary MB tumors

RNA was isolated from primary MB tissues and libraries were generated and sequenced at Genome BC as previously described[2,3] or at The Centre for Applied Genomics (TCAG), using NEB Ultra II Directional mRNA (NEB, E7760) Library Prep Kit. TCAG libraries were sequenced on HiSeq 2500 high throughput module PE126.

DNA methylation array

HumanMethylation450 methylation array and 850K Methylation EPIC array were generated as previously described at TCAG[2,4]. DNA methylation array results were used to annotate the subgroup identities of the MB tumors as previously described[4].

Chromatin Immunoprecipitation (ChIP)

Tumors were processed for chromatin immunoprecipitation (ChIP) as previously described with minor modifications[1]. Prior to sample preparation, cell lysis buffer, nuclear lysis buffer and cell dilution buffer were supplemented with protease inhibitor cocktails (Sigma, 11836170001). To start,10mg of dynabeads A (Invitrogen 10002D) and G (Invitrogen 10004D) were washed three times with 5 mg/mL bovine serum albumin (BSA) in PBS (PBS/BSA), resuspended in 400 uL of PBS/BSA and 5 ug of anti H3K27Ac antibody (Active Motif, 39133), H3K27me3 antibody (Diagenode, C15410069) (for MB tissues), H3K27me3 antibody (Cell Systems Technology, 9733) (for MB051 cells only) or FLAG antibody (Sigma, F1804). The dynabead mixture was then incubated for 6 hours to overnight in PBS/BSA, rotating at 6 rpm in 4 degrees Celsius until addition to the chromatin lysate.

For sample preparation, nuclei were isolated from tissues using dounce homogenizer and cell lysis buffer (10mM Tris-HCL ph8, 10mM NaCl, 0.2%NP40). Isolated nuclei were spun down 2500g at 4C for 5 minutes and resuspended in 300uL nuclear lysis buffer (1% SDS, 10mM EDTA, 50mM Tris-HCl pH8.1). Chromatin lysates were fragmented to 200-300 bp using Bioruptor (Diagenode) with the following settings: high amplitude – 30s on, 30s off for 30-45 cycles. 10 uL of lysate was kept as input control, set aside in 4C overnight. Remaining volume was mixed with 900 uL of cell dilution buffer (1% Triton, 2mM EDTA, 150mM NaCl, 20mM Tris-HCl pH 8.1). Dynabead mixture was then washed twice with PBS/BSA, resuspended in 800 uL of cell dilution buffer and mixed with the chromatin lysate + cell dilution buffer.

Diluted chromatin was incubated in cold room overnight on a rotator (6 rpm). On the following day, dynabeads were collected on magnetic stands and resuspended in 1

mL of LiCl RIPA Wash Buffer (50mM HEPES pH 7.6, 1mM EDTA, 0.7% Na Deoxycholate, 1% NP-40, 0.5M LiCl). Resuspended beads were placed in rotator (6 rpm) at coldroom for 5 minutes. Wash step was repeated 5 times, beads were washed once with TE buffer (10mM Tris pH 8.1, 1 mM EDTA) and resuspended in 100 uL of Decrosslinking buffer (1% SDS and 0.1M NaHCO3). Decrosslinking buffer was added to input samples at the same time as corresponding immunoprecipitation samples. Immunoprecipitation and input lysates were decrosslinked overnight on thermomixer at 65C (900 rpm). Following day, 20uL of Proteinase K (20mg/mL) was added to chromatin supernatant and incubated for 1 hour at 55C. Reverse crosslinked DNA was purified using GenepHlow Gel/PCR Kit (FroggaBio, DFH300). Subset of ChIP cohort were not processed in-house and submitted to Active Motif for commercial ChIP-Seq service (cohort 2). Sample cohort information are available in supplementary table.

ChIP sequencing library generation

In-house ChIP-Seq libraries were prepared using NEB Ultra DNA Library Prep Kit for Illumina (NEB7645) as instructed by the kit (cohort 1). Additional batch of H3K27ac libraries (cohort 2) were generated using service from Active Motif.

H3K27ac HiChIP library preparation

Tumors used for HiChIP were resected from operating room, dissociated mechanically and enzymatically (Sigma, 11097113001), crosslinked in 1% Formaldehyde (Sigma, F8775) for 5 minutes, snap frozen in liquid nitrogen and stored at -80C until use. In total, 3 Group3 (G3), 3 Group4 (G4) and 2 SHH MB tumors were

processed for HiChIP library generation as previously described with minor modifications (Chang, 2015). Prior to sample preparation, Cell Lysis Buffer, Nuclear Lysis Buffer and Cell Dilution Buffer were supplemented with protease inhibitor cocktails (Sigma, 11836170001). Briefly, 10 million crosslinked cells were resuspended in Cell Lysis Buffer (10mM Tris-HCL ph8, 10mM NaCl, 0.2%NP40), spun down at 2500g for 5 minutes, resuspended in 50 uL of 0.5% SDS and incubated at 62C for 8 minutes. SDS was quenched by adding 25 uL of 10% Triton X-100, 145 uL of H2O and nuclei were incubated for 15 minutes at 37C. Chromatin was digested in situ by adding 25 uL of 10X DpnII buffer (NEB B0543) and 10 uL of 10U/uL DpnII (NEB R0543) and incubating at 37C 900rpm for 2 hours. DpnII was then heat inactivated at 62C for 20 minutes, nuclei were spun down and resuspended in 250uL of 1X NEB2 buffer. Restriction fragments were repaired with biotin-dATP by addition of 37.5 uL of 0.4 mM biotin-dATP (Invitrogen, 1924016), 1.5 uL of 10 mM dCTP (Invitrogen, 18253013), 1.5 uL of 10 mM dGTP (Invitrogen, 18254011), 1.5 uL of 10 mM dTTP (Invitrogen, 18255018), 8 uL of 5U/uL DNA Polymerase I, Large (Klenow) Fragment (NEB, M0210). Tubes were incubated at 37C for 50 minutes to facilitate end repair. Proximity ligation was performed by addition of 947 uL of ligation master mix, consisting of 120uL 10X NEB T4 DNA ligase buffer (NEB, B0202), 100uL of 10% Triton X-100, 12 uL 10mg/mL BSA, 5 uL 400 U/uL T4 DNA ligase (NEB, M0202) and 663 uL of H2O. Following incubation at 16C overnight, nuclei were collected through centrifugation at 2500g for 5 minutes and resuspended in 300 uL of Nuclear Lysis Buffer (1% SDS, 10mM EDTA, 50mM Tris-HCl pH8.1). Chromatin was fragmented to average size of 1000 bp using bioruptor with following settings - High Amplitude, 30s on 30s off, 5 cycles. Sonicated chromatin was

diluted with 5X volume of Cell Dilution Buffer. 7.5 ug of H3K27Ac antibody (Abcam, ab4729) was added to the tube and incubated overnight at 4C with rotation (6 rpm). Following day, 60 uL of Dynabeads A (Invitrogen 10002D) and G (Invitrogen 10004D) were washed twice in 0.5% BSA in PBS, once with Cell Dilution Buffer and added to each sample tube. Samples were incubated for 2 hours at 4C with rotation (6 rpm), beads were separated on magnetic stand and washed three times with 500 uL Low Salt Wash Buffer (20 mM Tris-HCl pH 7.5, 2 mM EDTA, 0.1% SDS, 1% Triton X-100, 150 mM NaCl), three times with 500 uL High Salt Wash Buffer (20 mM Tris-HCl pH 7.5, 2 mM EDTA, 0.1% SDS, 1% Triton X-100, 500 mM NaCl), 500 uL of LiCl Wash Buffer (10 mM Tris-HCl pH 7.5, 1 mM EDTA, 250 mM LiCl, 1% NP40, 1% Sodium deoxycholate) and once with 500 uL of TE (10mM Tris pH 8.1, 1 mM EDTA) Buffer. Beads were resuspended in 150uL of elution buffer (1% SDS and 0.1M NaHCO3) and incubated at room temperature for 15 minutes on thermomixer (1000 rpm). Supernatant was saved, additional 150 uL of elution buffer was added to the beads and incubated at 65C for 15 minutes on thermomixer. (1000 rpm). Two supernatant elutions were combined (300 uL) and proteinase K treated (15uL of 20mg/mL) at 55C for 45 minutes. Samples were incubated at 68C for 3 hours afterward to fully reverse crosslink chromatin. DNA was purified using Zymo DNA Clean and Concentrate (Zymo, D4013) as per manufacturer’s instructions and eluted in 10uL of H2O. 6 uL of Streptavidin T1 magnetic beads (Invitrogen, 65601) was washed with 0.5 mL of Tween Wash Buffer (5mM Tris-HCl pH 7.5, 0.5 mM EDTA pH 8.0, 1M NaCl, 0.05% Tween-20), resuspended in 10 uL of 2X Biotin Binding Buffer (10 mM Tris-HCl pH 7.5, 1 mM EDTA pH 8.0, 2M NaCl) and added to purified DNA. Sample tubes were incubated at room temperature for 15 minutes on

thermomixer (700 rpm). After capture, beads were washed twice with 500 uL Tween Wash Buffer, incubating at 55C for 2 minutes with shaking (1000 rpm) for each wash. Beads were washed by 50 uL 1X Nextera Tagment DNA TD buffer (25 uL of 2X buffer + 25 uL of H2O), and tagmented with Illumina adapters using Nextera DNA Sample Preparation Kit (Illumina, FC-121-1030). Beads were washed once with 500 uL of 50 mM EDTA at 50C for 30 minutes with occasional mixing, twice with 500 uL of Tween Wash Buffer for 2 minutes and once with 500 uL of 10mM Tris-HCl pH 7.5 for 1 minute at room temperature. Beads were separated on magnet and resuspended in 25 uL of Q5 Hotstart mastermix, 1 uL of Nextera Index primer 1 (orange tube), 1 uL of Nextera Index Primer 2 (white tube) and 23 uL of H2O. DNA was amplified for 8-9 cycles with the following PCR conditions: 72°C, 5 min; 98°C, 1 min; 8 cycles of (98°C, 15 sec; 63°C, 30 sec; 72°C, 1 min); 72°C, 1 min; 4°C, hold. SureSelect beads were used to purify libraries with size distribution 300-700 bp.

RNA-Seq on ZIC1 mutant construct transduced cells

For RNA-seq in D283 cells, cells were infected with control (pCDH-mcherry) or ZIC1 viruses (pCDH-mcherry_ZIC1 WT/mutants) at high MOI to ensure more than 90% of cells were infected. Cells were then cultured for 3 days and then collected for RNA-seq. For RNA-seq in D425 cells, cells were collected after proliferation assay. Mcherry percentage was analyzed by flow cytometry for each sample to ensure more than 90% of cells were mcherry+ at the time of collection.

For RNA-seq in cerebellar cells or GNPs, cerebellar cells were isolated from Atoh1-GFP mice at P4 as described above. Cells were infected with control (pCDH-

mcherry) or ZIC1 viruses (pCDH-mcherry_ZIC1 WT) and cultured in GNP culture medium with SHH for 3 days. An aliquot of cerebellar cells was collected for RNA-seq. The rest of the cells were digested and sorted to enrich for infected GNPs (mcherry+/GFP+). After sorting, cell pellets were collected for RNA-seq. Two biological replicates were prepped for each construct (mCherry+ vs. WT ZIC1) for each cell type (granule cells and GNP).

Isolation of cerebellar granule cells or GNPs

Cerebellar cells were isolated from the cerebellum as described previously [5]. Briefly, cerebellum from postnatal day 5 (P5) mice were digested with high glucose DPBS (ThermoFisher) containing 10 U/ml papain (Worthington), 200 µg/ml L-cysteine and 250 U/ml DNase (Sigma) for 30 minutes. Tissue was triturated to obtain a single cell suspension and then centrifuged through a 35% and 65% Percoll gradient (Sigma). Cells in the layer between 35% and 65% Percoll were washed once with DPBS containing 0.02% BSA and resuspended in GNP culture medium (Neurobasal supplemented with B27 (50 x), Sodium pyruvate (100 x), Penicillin-Streptomycin (100 x) and Glutamax (100 x)). Granule cells or GNPs were enriched by depleting the adherent cells through two incubations in PDL-coated plates for 20 minutes each time. Enriched granule cells and GNPs were cultured with GNP culture medium supplemented with 3 ug/ml SHH (Peprotech) in PLD-coated plates. For isolation of pure GNPs, cerebellar cells were isolated from Atoh1-GFP mice at P5 as described above. After washing once with DPBS containing 0.02% BSA, cells were suspended with DPBS containing 5%

FBS (ThermoFisher). GNPs with strong GFP expression (~40%) were sorted and cultured with the GNP culture medium as described above.

G3 MB cell culture for MTS and proliferation assay

For MTS and cell proliferation assay with ZIC1/4 constructs, D425 and D283 were cultured in suspension with Neurocult media (Stemcell Technologies, Cat#05750). 1 full bottle (450mL) was supplemented with 5 mL N-2 Supplement (THERMO FISHER, Cat # 17502-048), 10 mL B-27 Supplement minus vitamin A (THERMO FISHER, Cat # 12587-010), 5 mL Antibiotic: Antimycotic (100X), 5 mL Glutamax (THERMO FISHER, Cat # 35050-061), 1 mL Bovine Serum Albumin (SIGMA-ALDRICH, Cat # A8412) and 1 mL Heparin (1mg/mL) (SIGMA-ALDRICH, Cat # H3393). Right before use, 2.5 uL Human rEGF (200ug/ml) and 125 uL Human bFGF (4ug/ml) were added to 50 mL of complete Neurocult media.

For mutant ZIC1 construct experiments, D425 was cultured in suspension with neurocult media recipe described above. D283 was cultured with EMEM + 10% FBS in suspension.

MTS and proliferation assay for G3 MB cell lines using BFP, ZIC1, ZIC4, ZIC1/4 constructs

MTS assay was conducted on D425 cells transduced with BFP empty vector, ZIC1, ZIC4 and ZIC1/4 constructs according to manufacturer's instructions (Promega #G3580). 2000 cells were seeded in 100 uL complete neurocult media supplemented with growth factors for each 96 well position. 6 wells were used for each construct per

day per biological replicate. On day 3, 20 uL of MTS dye were added for each well and incubated at 37 degrees for 4 hours prior to taking measurement. Day 6 samples were topped up with 100 uL of fresh media on day 3. On day 6, 35 uL of MTS dye was added for each well from day 6 plates and incubated at 37 degrees for 4 hours prior to taking measurement. 3 Biological replicates were used for the experiment per construct.

For proliferation assay with BFP empty vector and ZIC1/4 constructs, 50k cells were seeded in 1 mL Neurocult media per 24 well plate. Cells were fed on day 3 by transferring to T25 suspension flasks and topping up with 4 mL of fresh neurocult media. Additional 5 mL media top up was provided on day 5. Cells were counted on day 1, 3, 5 and 7. Experiments were conducted in triplicates for each cell line per day per construct.

D283 cell competition assay with ZIC1 mutant constructs

D283 cells were infected with control (pCDH-mcherry) or ZIC1 viruses (pCDH-mcherry_ZIC1 WT/mutants) at proper virus/cell ratio to ensure about 40% ~ 70% cells being infected and a similar infection efficiency across different samples in a single experiment. Three days after infection, an aliquot of cells was collected and examined by flow cytometry for baseline percentage of mcherry+ cells for each sample. Cells were then cultured in triplicates for 15 days and cell aliquots were collected and examined for the percentage of mcherry+ cells at regular intervals. To analyze the data, percentage of mcherry+ cells at each time point was normalized to that of baseline (Day 3) for each sample.

D425 cell proliferation assay with ZIC1 mutant constructs

D425 cells were infected with control (pCDH-mcherry) or ZIC1 viruses (pCDH-mcherry_ZIC1 WT/mutants) at high MOI to ensure more than 90% of cells were infected. Three days after infection, mcherry+ cells were further enriched by flow cytometry and expanded by culturing for additional 4 days. Cells were then counted and plated at an equal number in triplicates for each sample (Day 0). Cell number was counted on Day 2, 4 and 6 after plating.

Cycloheximide (CHX) pulse chase

D283 cells were infected with ZIC1 viruses (pCDH-GFP_FLAG-ZIC1 WT/mutants) at virus/cell ratio to ensure 60% ~ 80% transduction efficiency across different samples in a single experiment. Three to four days after infection, cells were collected for western blot (0 hours) or were treated with 50 ug/ml CHX (Sigma) for 2, 5, 8, 12 hours. Protein concentration was determined by BCA assay using Pierce™ BCA Protein Assay Kit (ThermoFisher).

For CHX pulse chase assay in enriched granule cells and GNPs, cells collected from P6 cerebellum were infected with ZIC1 viruses (80% ~90% transduction efficiency) and cultured with GNP culture medium supplemented with 3 ug/ml SHH (Peprotech) in PLD-coated plates for 3 days. Cells were then treated with 20 ug/ml CHX for indicated times before collecting for western blot.

ZIC1 Immunohistochemistry on developing human cerebellum slides

Immunohistochemistry was performed as previously described[6]. Briefly, midsagittal paraffin-embedded sections of 11, 14, 17 PCW human cerebellums were deparaffinized, rehydrated and treated with the ZIC1 antibody (Sigma Aldrich; Catalog number: HPA004098) at a dilution of 1:100. Following immunostaining, sections were counterstained with DAPI and mounted using Vectashield mounting medium (Vector labs; Catalog number H-1000).

ZIC1 Immunofluorescence on MB051 harvested from NSG mice

Whole brains were collected from MB051 (patient derived Group 3 MB xenograft line) injected NSG mice upon end point. Collected brains were fixed in 4% PFA overnight. Next day (day 2), after discarding 4% PFA, brains were washed with PBS (5 times) and soaked in 10% sucrose overnight at 4 degrees. Next day (day 3), 10% sucrose was discarded, the brains were washed with PBS (5 times) and soaked in 30% sucrose overnight at 4 degrees. Next day (day 4), 30% sucrose was discarded, brains were washed with PBS (5 times) and embedded in OCT to create OCT blocks. These blocks were stored at -80C for long term storage. OCT blocks were placed at -20C for 4 hours prior to creating cryosection slides. Sample slides were stored at -80C until used for immunofluorescence.

On the first day of immunofluorescence, tissue slides were air dried for 20 minutes at room temperature, washed with 10% Triton X-100 in PBS (4 x 10 minutes), blocked in 10% Triton X-100 5% goat serum in PBS for 1 hour and stained with primary antibody (HPA004098) in 10% Triton X-100 5% goat serum in PBS for overnight at 4C. On the second day, tissue slides were washed with 10% Triton X-100 in PBS (4 x 10

minutes), stained with secondary antibody (Thermo Fisher, A27039) in PBS + 10% triton + 5% goat serum for one hour at room temperature. Afterward, the slides were washed with 10% Triton X-100 in PBS (4 x 10 minutes), stained with DAPI in 10% Triton X-100 with PBS for 5 minutes and washed with Triton X-100 with PBS (3 x 3 minutes). Slides were visualized on a Leica DMi8 microscope.

Primers used for the study

Primers used for qRT-PCR and Sanger sequencing for the study are described in the supplementary table.

Neuronal differentiation score quantification

Neuronal differentiation levels were estimated from bulk RNA-Seq data by combining expression level of previously published 39 genes highly expressed in neuron like cells from Group 3 and Group 4 medulloblastoma tumors[7]. Group 3 and Group 4 tumors identified to express ZIC1/4 transcripts as monoallelic or biallelic were subsetted from the bulk RNA-Seq DESeq2 object. Afterward, read counts for the G3/G4 MB neuronal genes were normalized to the average count calculated across the monoallelic/biallelic cohort. Normalized expression level of all genes were summed to create a single neuronal differentiation score for all G3/G4 ZIC1/4 monoallelic and biallelic samples.

Derivation of human glutamatergic lineage trajectory from human cerebellum single cell RNA-Seq

Human glutamatergic lineage differentiation trajectory was defined from published human developing cerebellum as previously described[3]. For identification of excitatory Deep Cerebellar Nuclear neurons (eDCN), early and late UBC cluster were first aggregated to create a new ‘pan UBC’ cluster. Afterward, the ‘pan UBC’ cluster was subclustered with high resolution (0.5). New subcluster with all TBR1+ cells were identified as eDCN cluster.

Somatic variant calling

For WGS samples with matching normal DNA, somatic variants were called using eight variant callers as previously described[8]. For WGS samples without matching normal DNA, pseudo normal DNA bam file was generated using 1000 genome data and used as control. 26 randomly selected same sex bam files from 1000 genome project were realigned using identical parameters as tumor DNA and down-sampled to 200/26 coverage using samtools ‘view -s’ command. Resulting bams were merged and sorted using samtools. For these samples, all mutations registered in gnomAD SNP database from Genome Analysis Tool Kit (GATK) Resource Bundle were removed from the variant calls.

Identifying subgroup-specific histone marks

Merged H3K27ac, H3K27me3 and SE bed files were generated by merging all peak coordinates for each mark with bedtools merge. Reads for merged coordinates were obtained from bam files using featureCounts v 1.6.2 with parameters -s 0 -B -d 0 -p -D -Q 10 2000 -C [9]. Subgroup enriched H3K27Ac, H3K27me3 peaks and SEs were defined

by using DESeq2 differential expression analysis on the read count matrix for each histone mark and SE. Batch effects were corrected in the linear model by adding the batch information in design formula *'~ batch + subgroup'*, where subgroup has levels 'WNT', 'SHH', 'G3', and 'G4'. Differential peaks were defined as those with a Benjamini-Hochberg adjusted p value < 0.01 and absolute log2foldchange greater than 1.5 for H3K27Ac, 1.0 for H3K27me3 and 1.0 for SE. Subgroup specific peaks were defined by intersecting differential peaks from all three way comparisons – WNT specific peaks were defined as intersection between WNT enriched peak list vs. SHH, vs. G3 and vs. G4. For G3/4 and WNT/SHH enriched peaks, those with absolute log2foldchange smaller than 1 compared to matched subgroup but significantly different to both of the other subgroups with log2foldchange greater than 1 were regarded as G3/4 or WNT/SHH specific peaks.

Subgroup enriched H3K27me3 peaks were also identified by a complementary method examining the recurrence of peaks in subgroups instead of the comparing peak intensities. A peak was defined as 'subgroup specifically recurrent' if present in at least 3 samples within a subgroup but present at most 1 time in each other subgroup. For G3 and 4 recurrent peaks, those that are recurrent in G3 and/or G4 but not in SHH or WNT were also considered.

Identification of MB enhancer gene interactome

We incorporated two methods to identify enhancers-genes regulatory network with HiChIP loops and Spearman's rank correlation test for H3K27ac signals and genes expression levels. Enhancer gene pairs were connected according to the loop anchors

that were either inside enhancer or promoter region of gene (upstream 2kb from transcription start site). Read counts of each gene and enhancer were normalized by sample library size. We filtered genes for protein coding status related to loops. After finalizing the enhancer gene pairs in all subgroups, a correlation test was then applied to the list of curative enhancer gene pairs from HiChIP data. P-values calculated were combined and corrected for multiple testing using Bioconductor package qvalue. Correlation coefficients with FDRs less than 0.1 were preserved. For each enhancer, genes were ranked by their coefficients, and the top first gene was selected as the potential target gene. If the second best or third best correlating genes for that enhancer had the difference between spearman correlation coefficients for the top first correlating genes were less than 0.1, the second/third best correlating gene was also regarded as potential target gene. Finally, we refined the enhancer gene interactomes list by taking enhancer gene pairs both identified in the HiChIP loops and showed with significantly strong correlations.

Peak saturation analysis

ChIP cohort samples were randomly ordered and number of identifiable peaks were calculated for each N from 1 to total number of samples (123 for 27ac, 63 for 27me3). For example, for N = 3, total number of unique peaks from 3 randomly picked samples was collected. Sampling was repeated until all samples in the cohort were exhausted. Total of 10 iterations were performed to generate average number of peaks as well as standard deviations for each N.

Core regulatory networks identification

Identifying core regulatory circuits (CRC) for interactions among SE-associated TFs was conducted using two measurements, i.e., 1) inward and outward binding degree (IN/OUT) and 2) CRC enrichment score (https://github.com/linlabcode/CRC) with slight modification. The inward degree for TF_i was defined as the number of TFs with binding motifs in the SE associated with TF_i. The outward degree was defined as the number of SEs containing binding motifs for TF_i. IN/OUT degrees were calculated for all SE associated TFs. For SEs not directly overlapping annotated genes, nearest gene was assigned as the regulatory target, if it contained H3K27Ac marks at the promoter. All SE-associated TFs annotated to regulate another TF were included in the node-list for network construction. Auto-regulatory TFs were defined as SE associated TFs that self-regulate the associated overlaying SEs on top of other SE targets. Auto-regulatory circuits were defined as circuits comprised exclusively of auto-regulatory TFs that regulate all other constituents of the circuit. The CRC enrichment score for a given TF_i per sample was defined as percentage of TFs that form auto-regulatory circuits with TFi / total number of TFs that form auto-regulatory circuits. Emphasis of the modified CRC enrichment score was to directly examine overall TF connectivity within auto-regulatory circuits instead of assessing total number of associated auto-regulatory circuits.

Subgroup specific regulatory TFs were identified by using both IN/OUT degree and enrichment score. For subgroup specific regulatory TFs measured by enrichment score, average score for each auto-regulatory TF_i was computed, and student t test was conducted across each subgroup. A subgroup specific TF_i was defined as those with p-values of TF_i against the rest of subgroups smaller than 0.05. A WNT/SHH or G3/4 specific TF_i was nonsignificant between close subgroups. Subgroup average inward and

outward degree for each TF was calculated, and the average total degree (IN degree + OUT degree) was multiplied with recurrence. Total degree of a TF was then scaled by the maximum degree across all subgroups. Subgroup specific TF was identified when the max degree for the TF of other three subgroups was below the bottom 10% quantile of all TFs.

Transcriptional regulatory network

To generate extended TF regulatory network, motif analysis of TF binding within enhancers was expanded to all enhancers. Putative Nucleosome-Free Regions (NFR) were identified by recognizing 'dips' in 27Ac signal flanked by 2 higher 27Ac signal summits using an adapted algorithm[10]. Enriched TF binding sites were determined for all of the NFR valleys within each subgroup using FIMO in MEME suite v 5.0.5 with p value threshold of 1×10^{-4} [11]. TF position-weight matrices (PWM) were taken from Transfac and JASPAR 2014. For each TF, contingency tables containing the number of peaks and SEs overlapping and non-overlapping with the respective transcription factor were constructed. The significance of enrichment of TFs in enhancers with subgroup-specific activity was determined by comparing TF enrichment in subgroup specific peaks with those in subgroup conserved peaks using the Chi-squared test. The resulting p-values were corrected for multiple testing (FDR < 0.01). TF enrichments were calculated as the odds ratio between observed read counts over expected read counts.

Bayesian model for RNA-Seq and ChIP-Seq monoallelic calls.

Bayesian model was used to normalize ChIP/RNA-Seq SNP frequency by the allelic frequency in WGS as previously described[12]. Briefly, to infer an allelic imbalance from genotypes while integrating technical bias information, we assume that the tumor purity of WGS samples, RNA-Seq samples, and ChIP-Seq data were the same and that the haplotype ratios are exactly consistent with allele ratio. We jointly inferred the true allelic imbalance ratio of a given SNP in ChIP-Seq/RNA-Seq data from allele depth in all data sets based on the allele ratio of the same heterozygous SNP in WGS. For the i_{th} sample, assume *n* denoted the total number of reads covering a SNP position at *j* and *m* the number of reads reporting the alternative allele (n, m ∈ N). A beta-binomial distribution, i.e., the binomial distribution in which the probability of success is integrated out given that it follows the beta distribution:

$$m_{ij} \sim Bin\left(n_{ij},\, \theta_{ij}\right)$$

Where θ_{ij} was modeled by beta distribution:

$$\theta_{ij} \sim Beta\left(\alpha_{ij},\, \beta_{ij}\right)$$

Which $\alpha,\ \beta$ were the shape parameters in beta distribution.

The beta-binomial distribution controlled for overdispersion, i.e., the increased variance in next-generation sequencing data cannot be captured in the standard binomial model. We re-parameterize α and β in terms of the precision of the beta-binomial distribution to gain a more intuitive interpretation of the parameters. *M*, (precision) and the mean probability that a reference read is observed followed basic binomial characteristics, θ:

$$\alpha = \theta \times M$$

$$\beta = (1-\theta) \times M$$

Alternative allele ratio from RNA-Seq or ChIP-Seq sample were normalized by allele ratio from WGS sample as previously described[12]:

$$\theta = \frac{\varepsilon \times \rho}{\varepsilon \times \rho + (1-\rho) \times (1- \varepsilon)}$$

Which ρ denoted allele ratio in WGS data and ε denotes true allele imbalance ratio from RNA-Seq or ChIP-Seq data (the probability that the reads captured by antibody are from alternative allele), and θ is the allele ratio observed from ChIP-Seq or RNA-Seq data. This formulation was the process to obtain the posterior probability of the allele imbalance ratio. And the WGS allele ratios were served as the prior distribution.

$$p(\varepsilon|\rho, \mu, \lambda) = p(f(\varepsilon, \rho)|\mu, \lambda)\frac{\partial f(\varepsilon, \rho)}{\partial \varepsilon}$$

We set precision M to 1,000 to get the best prediction. In order to compute the true allele imbalance ratio ε of SNPs among subgroups in a probabilistic framework, we took advantage of the parametric model to determine the distribution of the true allele imbalance ratio. To that end we randomized the ε from 0 to 1 with 20,000 times sampling from the probability density function of ε and to see the probability when ε is less or equal to 0.5. The nominal p-value for each one-sided test was then determined by the number of simulated instances where ε was larger/smaller than 0.5. The true allele imbalance ratio was determined as the point with highest probability density.

Monoallelic event identification.

Reads that contained Ns in their cigar string from RNA-Seq data were split by GATK (v 4.1.2.0) SplitNCigarReads function[13]. Haplotypes of the counterpart SNPs in RNA-Seq and ChIP-Seq data were calculated using bcftools mpileup and call with

parameters --min-BQ 20, --min-MQ 20, -Ou, -mA. The cutoff of allele depth of each SNP in ChIP-Seq data and RNA-Seq data was set as ≥ 5 and ≥ 10 respectively in order to obtain robust genotypes and allele frequencies. For monoallelic RNA-Seq analysis, we only considered SNPs located in exon regions. Additionally, SNPs in polyA regions were removed because of the unreliable variant allele frequencies[14]. To avoid false positive monoallelic SEs as result of copy number driven LOH, we excluded monoallelic super enhancers overlapping with copy-number deletions identified from WGS Control FREEC output described above.

For a heterozygous SNP_i within a sample in WGS data, we determined the statistically inferred true allele imbalance ratio of its counterpart nucleotide in ChIP-Seq/RNA-Seq data by Bayesian model. For the convenience of computation, the allele imbalance ratio of a heterozygous SNP_i was converted to the difference between the ratio of dominant allele and 0.5. We only considered genes/peaks with at least one heterozygous site. True allele imbalance ratio and FDR adjusted p values were computed for all sites inside a gene/peak. To evaluate per-gene/peak allele imbalance ratio, the allele imbalance ratio of each site was weighted by its allele depth, as allele depth reflected the strength of the gene expression or histone signal and the inference accuracy increased when allele depth increased. We also combined the FDR et each site using Stouffer's method, weighted by allele depth as well. To exclude randomized monoallelic heterozygous SNPs, phased information was used to ensure the sites in a monoallelic gene/peak shared the same dominant allele.

We then defined a monoallelic expressed gene or enriched peak that has combined FDR < 0.05 and has a gene/peak level estimated allele imbalance ratio ≥ 0.4

(with dominant allele ratio ≥ 0.9). And we defined a dual-allelic expressed gene or enriched peak with FDR < 0.05 and estimated allele imbalance ratio ≤ 0.2 (with dominant allele ratio ≥ 0.5 and ≤ 0.7). Genes with dominant allele ratio > 0.7 and < 0.9 were defined as allelic biased.

Summary of ZIC1/4 epigenetic repression, CNA and SNV across MB

For breakdown of ZIC1/4 allelic expression pattern, copy gain in SHH, copy loss in G3/G4 MB and SNVs in the validation cohort with both RNA-Seq and WGS data available (N = 251), germline heterozygous SNPs were called and used even if the heterozygosity was lost due to copy number deletion in the tumor. Samples harboring germline SNPs within ZIC1/4 exons with allelic ratio in blood between [0.3, 0.7]) and read depth ≥ 7 in both blood and tumor were also included in the summary output (piechart). Subgroup enriched recurrent CNAs (copy gain in SHH, copy loss in G3, G4 MB) discovered from Affymetrix SNP6 array data were inferred from WGS Control-FREEC output as described above.

Identification of subgroup enriched genes with ZIC1 motifs in promoter

Homer package (v 4.9) was used to identify transcription factors (TFs) whose promoters harbor ZIC1 binding motifs. Then, subtype-specific differentially expressed genes (DEGs) for SHH, WNT, Group 3 and Group 4 MB were identified by mutual comparison between the RNA-seq profiles of these groups and normal samples. For example, Group 4 MB-specific DEGs were identified by comparing the RNA-seq profiles of Group 4 MB samples with the RNA-seq profiles of Group 3 MB, SHH MB, WNT MB

and normal cerebellum samples. Next, we identified the overlapping genes between the subtype specific DEGs and TFs that are targeted by ZIC1.

Normalized read calculation for FLAG-ZIC1 ChIP-Seq in D283

For FLAG ZIC1 ChIP-Seq on D283 cells transduced with wildtype and G4 mutant ZIC1 constructs, raw sequencing data was aligned to hg19 genome using bowtie2. Sequencing depth normalized read counts were obtained for each peak (number of reads on a peak * 1,000,000 / total number of mapped reads) for each library using bedtools multicov.

Normalized read calculation for FLAG-ZIC1 ChIP-Seq in GNP

For FLAG ZIC1 ChIP-Seq on GNP cells transduced with wildtype and G4 mutant ZIC1 constructs, raw sequencing data was aligned to mm10 genome using bowtie2. Raw read counts on merged peak coordinates were obtained using bedtools multicov. These counts were subsequently normalized by sequencing depth of the libraries.

Protein paint illustration

Protein paint illustration for ZIC1 was generated using the St. Jude protein paint online resource (https://proteinpaint.stjude.org/).

References

1. Mack, S. C. *et al.* Therapeutic targeting of ependymoma as informed by oncogenic enhancer profiling. *Nature* 553, 101–105 (2018).
2. Skowron, P. *et al.* The transcriptional landscape of Shh medulloblastoma. *Nat Commun* 12, (2021).
3. Hendrikse, L. D. *et al.* Failure of human rhombic lip differentiation underlies medulloblastoma formation. *Nature* 609, 1021–1028 (2022).
4. Cavalli, F. M. G. *et al.* Intertumoral Heterogeneity within Medulloblastoma Subgroups. *Cancer Cell* 31, (2017).
5. Tao, R. *et al.* *MYC* Drives Group 3 Medulloblastoma through Transformation of Sox2+ Astrocyte Progenitor Cells. *Cancer Res* 79, 1967–1980 (2019).
6. Haldipur, P. *et al.* Spatiotemporal expansion of primary progenitor zones in the developing human cerebellum. *Science (1979)* 366, 454–460 (2019).
7. Hovestadt, V. *et al.* Resolving medulloblastoma cellular architecture by single-cell genomics. *Nature* 572, (2019).
8. Suzuki, H. *et al.* Recurrent noncoding U1 snRNA mutations drive cryptic splicing in SHH medulloblastoma. *Nature* 574, 707–711 (2019).
9. Liao, Y., Smyth, G. K. & Shi, W. FeatureCounts: An efficient general purpose program for assigning sequence reads to genomic features. *Bioinformatics* 30, (2014).
10. Ramsey, S. A. *et al.* Genome-wide histone acetylation data improve prediction of mammalian transcription factor binding sites. *Bioinformatics* 26, (2010).
11. Bailey, T. L. *et al.* MEME Suite: Tools for motif discovery and searching. *Nucleic Acids Res* 37, (2009).
12. de Santiago, I. *et al.* BaalChIP: Bayesian analysis of allele-specific transcription factor binding in cancer genomes. *Genome Biol* 18, (2017).
13. van der Auwera, G., O’Connor, B. & Safari, an O. M. Company. Genomics in the Cloud: Using Docker, GATK, and WDL in Terra. *Genomics in the Cloud* (2020).
14. Wang, R., Nambiar, R., Zheng, D. & Tian, B. PolyA-DB 3 catalogs cleavage and polyadenylation sites identified by deep sequencing in multiple genomes. *Nucleic Acids Res* 46, (2018).
