# Supplementary material for: ZIC1 is a context-dependent medulloblastoma driver in the rhombic lip
Source: Nat Genet. 2025 Jan 3;57(1):88–102. doi: 10.1038/s41588-024-02014-z (PMC11735403; doi:10.1038/s41588-024-02014-z)
Supplement: Supplementary file 2 — Reporting Summary [file 41588_2024_2014_MOESM2_ESM.pdf]

nature portfolio

Corresponding author(s): Frank Huang
Paul Northcott
Michael Taylor

Last updated by author(s): Oct 10, 2024

Reporting Summary

Nature Portfolio wishes to improve the reproducibility of the work that we publish. This form provides structure for consistency and transparency in reporting. For further information on Nature Portfolio policies, see our Editorial Policies and the Editorial Policy Checklist.

Statistics

For all statistical analyses, confirm that the following items are present in the figure legend, table legend, main text, or Methods section.

n/a	Confirmed	
☐	☒	The exact sample size (*n*) for each experimental group/condition, given as a discrete number and unit of measurement
☐	☒	A statement on whether measurements were taken from distinct samples or whether the same sample was measured repeatedly
☐	☒	The statistical test(s) used AND whether they are one- or two-sided<br>*Only common tests should be described solely by name; describe more complex techniques in the Methods section.*
☐	☒	A description of all covariates tested
☐	☒	A description of any assumptions or corrections, such as tests of normality and adjustment for multiple comparisons
☐	☒	A full description of the statistical parameters including central tendency (e.g. means) or other basic estimates (e.g. regression coefficient) AND variation (e.g. standard deviation) or associated estimates of uncertainty (e.g. confidence intervals)
☐	☒	For null hypothesis testing, the test statistic (e.g. *F*, *t*, *r*) with confidence intervals, effect sizes, degrees of freedom and *P* value noted<br>*Give P values as exact values whenever suitable.*
☒	☐	For Bayesian analysis, information on the choice of priors and Markov chain Monte Carlo settings
☒	☐	For hierarchical and complex designs, identification of the appropriate level for tests and full reporting of outcomes
☒	☐	Estimates of effect sizes (e.g. Cohen's *d*, Pearson's *r*), indicating how they were calculated

Our web collection on statistics for biologists contains articles on many of the points above.

Software and code

Policy information about availability of computer code

Data collection	No software was used to collect data.
Data analysis	Bowtie2 2.2.1<br>https://github.com/BenLangmead/bowtie2<br>MACS2 2.1.1.20160309<br>https://pypi.org/project/MACS2/<br>Deeptools 3.1.3<br>https://github.com/deeptools/deepTools<br>Samtools 1.5<br>https://github.com/samtools/<br>Bedtools 2.27<br>https://github.com/arq5x/bedtools2<br>ROSE 0.1<br>https://bitbucket.org/young_computation/rose/src/master/<br>GenomicRanges<br>https://bioconductor.org/packages/release/bioc/html/GenomicRanges.html<br>STAR 2.7.4<br>https://github.com/alexdobin/STAR<br>HTSeq 0.6.0<br>https://github.com/simon-anders/htseq<br>DESeq2 1.26.0

https://github.com/mikelove/DESeq2
FeatureCounts 1.6.2
https://rdrr.io/bioc/Rsubread/man/featureCounts.html
CRC
https://github.com/linlabcode/CRC
FIMO 5.0.5 51
https://memesuite.org/meme/doc/fimo.html
Bowtie2 2.3.4
https://github.com/BenLangmead/bowtie2
HiC-pro 2.9.0
https://github.com/nservant/HiC-Pro
Hichipper 0.7.3
https://github.com/aryeelab/hichipper
BWA-mem 0.7.8
https://github.com/lh3/bwa
Platypus 0.8.1
https://github.com/andyrimmer/Platypus
EAGLE2
https://alkesgroup.broadinstitute.org/Eagle/#Xeagle2
Bcftools 1.9
http://www.htslib.org/download/
Affymetrix Power Tools 1.18.2 ThermoFisherScientific
https://www.thermofisher.com/ca/en/home/lifescience/microarrayanalysis/microarrayanalysis-partnersprograms/affymetrixdevelopersnetwork/affymetrixpower-tools.html
GISTIC 2.0.23
https://www.genepattern.org/modules/docs/GISTIC_2.0#gsc.tab=0
G profiler
https://biit.cs.ut.ee/gprofiler/Control-FREEC 10.3 32 http://boevalab.inf.ethz.ch/FREEC/
Genome Analysis Tool Kit 4.1.2.0
https://github.com/broadinstitute/gatk/releases
Complexheatmap 2.2.0
https://jokergoo.github.io/ComplexHeatmap-reference/book/
Homer 4.9
http://homer.ucsd.edu/homer/

Unique codes that were used in the study are available at the github page:
https://github.com/jjy-lee/ZIC_medulloblastoma

For manuscripts utilizing custom algorithms or software that are central to the research but not yet described in published literature, software must be made available to editors and reviewers. We strongly encourage code deposition in a community repository (e.g. GitHub). See the Nature Portfolio guidelines for submitting code & software for further information.

Data

Policy information about availability of data

All manuscripts must include a data availability statement. This statement should provide the following information, where applicable:

- Accession codes, unique identifiers, or web links for publicly available datasets
- A description of any restrictions on data availability
- For clinical datasets or third party data, please ensure that the statement adheres to our policy

The FLAG-ChIP-Seq, RNA-Seq data generated from ZIC1 mutant construct transduced G3 MB cell lines and granule cells have been deposited in the Gene Expression Omnibus (GEO) database under the accession numbers GSE217639, GSE217571 and GSE217638.
(Reviewer token: aladwoeqfhmphwv).

Bulk H3K27ac, H3K27me3 ChIP-Seq, RNA-Seq, WGS and H3K27ac hichip data generated from primary MB tumor samples in this study have been deposited in the European Genome-Phenome Archive (EGA) database under the accession code EGAS00001006741. The published MB bulk RNA-Seq data referenced in this study are available in the EGA database under the accessions EGAS00001001953, EGAD00001004347, EGAD00001004435, EGAS00001005826, EGAD00001001899 and EGAD00001004958. The published MB WGS data referenced in this study are available in the EGA database under the accessions EGAS00001001953, EGAD00001003125 and EGAD00001004347. The published MB 27ac ChIP-Seq data referenced in this study are available in the EGA database under the accessions EGAS00001001953. The Affymetrix SNP 6.0 data referenced during the study are available in the GEO database under the accession GSE37385. Expression array used for transcript abundance comparison between medulloblastoma subtypes are available in the GEO database under the accession GSE132269.

Multiple databases were used for annotation of SNPs and promoter, which were referenced in this study. These include the GRCh37 dbSNP151 (https://ftp.ncbi.nlm.nih.gov/snp/organisms/human_9606_b151_GRCh37p13/VCF/), GENCODE (v.19) (https://www.gencodegenes.org/human/release_19.html), the hg19 reference genome (https://hgdownload.soe.ucsc.edu/goldenPath/hg19/bigZips/), the hs37d5 reference genome (https://ftp943trace.ncbi.nih.gov/1000genomes/ftp/technical/reference/phase2_reference_assembly_sequence/), ERCC spike-in sequence (https://www.encodeproject.org/files/ENCFF908UQN/) and Caltech profile 3 spike-in sequence (https://www.encodeproject.org/references/946 ENCSR193ZXE/). snRNA-seq data from the developing human cerebellum were obtained through correspondence from Aldinger et al. 2021 and are available through the Human Cell Atlas (https://www.covid19cellatlas.org/aldinger20), the UCSC Cell Browser (https://cbl950dev.cells.ucsc.edu) or from Database of Genotypes and Phenotypes (dbGaP)(accession number phs001908.v2.p1). Bulk RNA-seq data from the developing human cerebellum were obtained through correspondence from Haldipur et al. 2019 and are available through the dbGaP (accession number phs001908.v2.p1).

Original codes used for the study are available via GitHub(https://github.com/jjy-lee/ZIC_medulloblastoma/).

Research involving human participants, their data, or biological material

Policy information about studies with human participants or human data. See also policy information about sex, gender (identity/presentation), and sexual orientation and race, ethnicity and racism.

Reporting on sex and gender	N/A
Reporting on race, ethnicity, or other socially relevant groupings	Patients diagnosed with cerebellar pediatric brain tumors were recruited from McGill University Health Centre and the Hospital for Sick Children/The Arthur and Sonia Labatt Brain Tumour Research Centre Biobank.<br><br>Primary tumors used in the study were obtained from the Medulloblastoma Advanced Genomics International Consortium (MAGIC) and International Cancer Genome Consortium (ICGC). All materials were collected after receiving written informed consents, including consent to publish the generated data, as per guidelines from Research Ethics Board from the following institutes: Agostino Gemelli University Hospital, Children's Hospital of Minnesota, Cooperative Human Tissue Network, David Geffen School of Medicine at University of California Los Angeles, Duke University, Emory University, Erasmus University Medical Centre, German Cancer Research Centre (DKFZ), Hospital Cantonal De Geneve, Hospital Infantil de Mexico Federico Gomez, Hospital Sant Joan de Deu, Ludwig Maximilans University, Masaryk University, McGill University, McMaster University, Memorial Sloan Kettering Cancer 969 Centre, Miami Children's Hospital, Portugese Cancer Institute, Queensland Children's Tumor Bank, Seattle Children's Hospital Fred Hunchinson Cancer Research Centre, Seoul National University Children's Hospital, Stanford University School of Medicine, The Chinese University of Hong Kong, Tohoku University, University of California San Francisco, University Health Network, Universitats Kinderklinik, Universite de Lyon, University of Arkansas, University of Calgary, University of Debrecen Medical and Health Science Centre, University of Pittsburgh, University of Ulsan Asan Medical Centre, University of Warsaw Children's Memorial Health Institute, Vanderbilt Medical Centre and Wolfson Children's Hospital.<br><br>Human cerebellar samples were obtained under approval from the Seattle Children's Research Institute IRB. Samples were collected with consent and in accordance with institutional and legal ethics guidelines, from the Human Developmental Biology Resource (HDBR), University College London and Newcastle University, United Kingdom, the Birth Defects Research Laboratory (BDRL) at the University of Washington, USA, and the Hôpital Necker-Enfants Malades in Paris, France.
Population characteristics	N/A
Recruitment	N/A
Ethics oversight	REB MCH003-26 approved by McGill University Health Centre (Montreal). REB 0020020238 and 1000055059 approved by the Hospital for Sick Children (Toronto).

Note that full information on the approval of the study protocol must also be provided in the manuscript.

Field-specific reporting

Please select the one below that is the best fit for your research. If you are not sure, read the appropriate sections before making your selection.

☒ Life sciences ☐ Behavioural & social sciences ☐ Ecological, evolutionary & environmental sciences

For a reference copy of the document with all sections, see nature.com/documents/nr-reporting-summary-flat.pdf

Life sciences study design

All studies must disclose on these points even when the disclosure is negative.

Sample size	Statistical methods were not used to predetermine the sample size. Sample sizes were chosen based on the availability of the primary tumors from the tumor bank.
Data exclusions	Tumors were excluded from the study if molecular classifier tumor identity turned out to be not medulloblastoma. Except these cases, no data were excluded from the study.
Replication	Experiments were performed in technical and biological replicates, and similar experiments performed across two different labs (Northcott lab, Taylor lab) lead to similar results.
Randomization	Randomization was not relevant to our study, as we were interested in interrogating molecular differences between known tumor identities.
Blinding	Blinding was not relevant to our study, as we were interested in interrogating molecular differences between known tumor identities.

Reporting for specific materials, systems and methods

We require information from authors about some types of materials, experimental systems and methods used in many studies. Here, indicate whether each material, system or method listed is relevant to your study. If you are not sure if a list item applies to your research, read the appropriate section before selecting a response.

Materials & experimental systems

n/a	Involved in the study
☐	☒ Antibodies
☐	☒ Eukaryotic cell lines
☒	☐ Palaeontology and archaeology
☐	☒ Animals and other organisms
☒	☐ Clinical data
☒	☐ Dual use research of concern
☒	☐ Plants

Methods

n/a	Involved in the study
☐	☒ ChIP-seq
☒	☐ Flow cytometry
☒	☐ MRI-based neuroimaging

Antibodies

Antibodies used

H3K27ac Active Motif 39133
H3K27me3 Diagenode C15410069
H3K27me3 Cell Signalling Tech 9733
FLAG Sigma-Aldrich F1804
ZIC1 Sigma-Aldrich HPA004098
ZIC4 ThermoFisher Scientific PA5-56392
H3 Abcam 1791
GAPDH Cell Signalling Tech 2118
Actin Cell Signalling Tech 8457
Rabbit IgG secondary antibody Thermofisher A27039

Validation

H3K27ac antibody has been validated by ChIP-Seq (PMID 29258295), ChIP-qPCR, immunofluorescence, western blot and dot blot analysis (Active Motif website).

H3K27me3 (Diagenode) antibody has been validated by ChIP-qPCR, ChIP-Seq (PMID: 24553142), dot blot and western blot (Diagenode website).

H3K27me3 (Cell Signaling) antibody has been validated by western blot, immunohistochemistry, immunofluorescence, flow cytometry, ChIP-Seq (PMID: 33259802) and cut&run (Cell Signaling Technology website).

FLAG antibody has been validated by immunoblotting, immunoprecipitation, immunohistochemistry, immunofluorescence, immunohistochemistry (Sigma-Aldrich website) and ChIP-Seq (PMID: 28215080).

Histone ChIP-Seq antibodies have also been validated with ChIP-qPCR within the aboratory, using primers against positive and negative control regions.

ZIC1 antibody has been validated by immunohistochemistry and immunofluorescence (Sigma-Aldrich website).

ZIC4 antibody has been validated by immunohistochemistry and immunofluorescence (Thermofisher website).

H3 antibody has been validated by western blot (Abcam website).

GAPDH antibody has been validated by western blot, immunohistochemistry, immunofluorescence and flow cytometry (Cell Signaling Technology website).

Actin antibody has been validated by western blot, immunofluorescence and flow cytometry (Cell Signaling Technology website).

Western blot antibodies have been validated with appropriate positive and negative control samples within the laboratory.

Eukaryotic cell lines

Policy information about cell lines and Sex and Gender in Research

Cell line source(s)

D283 (G3 MB cell line, male, PMID: 4056828), D425 (G3 MB cell line, male, PMID: 1904513), BT2019051 (patient derived G3 MB xenograft, male, derived from this study)D283 (G3 MB cell line, male, PMID: 4056828), D425 (G3 MB cell line, male, PMID: 1904513), BT2019051 (patient derived G3 MB xenograft, male, derived from this study)

Authentication

D283 and D425 were authenticated by STR profiling. BT2019051 was generated at the Hospital for Sick Children and passaged only in vivo.

Mycoplasma contamination

Cell lines were not tested for mycoplasma contamination.

Commonly misidentified lines
(See ICLAC register)

N/A

Animals and other research organisms

Policy information about studies involving animals; ARRIVE guidelines recommended for reporting animal research, and Sex and Gender in Research

Laboratory animals	Mus Musculus, NOD scid gamma mouse, 6-10 weeks of age
Wild animals	This study did not involve wild animals.
Reporting on sex	Findings in the study do not apply to one sex. Sex was not considered in the study design. Sex based analysis was only performed for determining bias in H3K27me3 peak presence on BCOR promoter (Extended Figure 2). For other analysis, sex based analysis was not performed, as there was no apparent bias in the manifestation of the observed genetic/epigenetic phenomenon.
Field-collected samples	This study did not involve samples collected from the field.
Ethics oversight	All mouse breeding and procedures were performed as approved by The Centre for Phenogenomics.

Note that full information on the approval of the study protocol must also be provided in the manuscript.

Plants

Seed stocks	*Report on the source of all seed stocks or other plant material used. If applicable, state the seed stock centre and catalogue number. If plant specimens were collected from the field, describe the collection location, date and sampling procedures.*
Novel plant genotypes	*Describe the methods by which all novel plant genotypes were produced. This includes those generated by transgenic approaches, gene editing, chemical/radiation-based mutagenesis and hybridization. For transgenic lines, describe the transformation method, the number of independent lines analyzed and the generation upon which experiments were performed. For gene-edited lines, describe the editor used, the endogenous sequence targeted for editing, the targeting guide RNA sequence (if applicable) and how the editor was applied.*
Authentication	*Describe any authentication procedures for each seed stock used or novel genotype generated. Describe any experiments used to assess the effect of a mutation and, where applicable, how potential secondary effects (e.g. second site T-DNA insertions, mosiacism, off-target gene editing) were examined.*

ChIP-seq

Data deposition

☒ Confirm that both raw and final processed data have been deposited in a public database such as GEO.

☒ Confirm that you have deposited or provided access to graph files (e.g. BED files) for the called peaks.

Data access links<br>*May remain private before publication.*	https://www.ncbi.nlm.nih.gov/geo/query/acc.cgi?acc=GSE217639<br>https://www.ncbi.nlm.nih.gov/geo/query/acc.cgi?acc=GSE217571<br>https://www.ncbi.nlm.nih.gov/geo/query/acc.cgi?acc=GSE217638<br>(Reviewer token: aladwoeqfhmphwv)<br><br>Bulk H3K27ac, H3K27me3 ChIP-Seq, RNA-Seq, WGS and H3K27ac hichip raw data generated from primary MB tumor samples in this study have been deposited in the European Genome-Phenome Archive (EGA) database under the accession code EGAS00001006741.
Files in database submission	narrowPeak and broadPeak files generated from MACS2 as well as count matrices for G3 MB cells transduced with ZIC1 constructs
Genome browser session<br>(e.g. UCSC)	https://genome.ucsc.edu/s/jjylee/hg19_ZIC1_locus_MB_ChIP

Methodology

Replicates	Primary tumors - single H3K27ac and/or H3K27me3 ChIP-Seq libraries were generated for different biological medulloblastoma tumors without technical replicates. 102 samples for H3K27ac, 63 samples for H3K27me3.<br><br>Cell lines with FLAG tagged ZIC1 constructs - at least two biological replicates were generated for WT ZIC1 ChIP-Seq and Group 4 mutant ZIC1 ChIP-Seq.
Sequencing depth	Each ChIP-Seq library was sequenced with at least 30M reads, typically resulting in >25M uniquely mapped reads. Primary tumor samples were sequenced with 126 bp (27ac, 27me3) or 101 bp (27ac - active motif) paired end reads. For cell lines, samples were sequenced with 151 bp paired end reads.
Antibodies	H3K27ac Active Motif 39133<br>H3K27me3 Diagenode C15410069

H3K27me3 Cell Signalling Tech 9733
FLAG Sigma-Aldrich F1804

Peak calling parameters

H3K27ac samples with inputs
macs2 callpeak -t IP_bam -c input_bam -f BAMPE -g hs --nomodel -B -q 1e-2

H3K27ac samples without inputs
macs2 callpeak -t IP_bam -f BAMPE -g hs --nomodel -B -q 1e-2

H3K27me3 samples
macs2 callpeak -t -t IP_bam -c input_bam -f BAMPE -g hs --nomodel --broad -B -q 1e-5 --broad-cutoff 1e-4

FLAG tagged ZIC1 ChIP-Seq (D283)
macs2 callpeak -t IP_bam -c input_bam -f BAMPE -g hs --nomodel -B -q 1e-5

FLAG tagged ZIC1 ChIP-Seq (GNP)
macs2 callpeak -t IP_bam -c input_bam -f BAMPE -g hs --nomodel -B -q 5e-2

Data quality

For H3K27ac, on average, each sample exhibited 30k peaks with FDR < 1e-2 and fold enrichment > 5. For H3K27me3, on average, each sample exhibited 5k peaks with FDR < 1e-4 and fold enrichment > 5. Unsupervised hierarchical clustering lead to robust recapitulation of known molecular subgroups of medulloblastoma for both marks, suggesting that the generated data set are able to identify known biological identities in unbiased manner.

Software

Bowtie2 2.2.1 https://github.com/BenLangmead/bowtie2
MACS2 2.1.1.20160309 https://pypi.org/project/MACS2/
Deeptools 3.1.3 https://github.com/deeptools/deepTools
Samtools 1.5 https://github.com/samtools/
Bedtools 2.27 https://github.com/arq5x/bedtools2
ROSE 0.1 https://bitbucket.org/young_computation/rose/src/master/
GenomicRanges https://bioconductor.org/packages/release/bioc/html/GenomicRanges.html
CRC https://github.com/linlabcode/CRC
FIMO 5.0.5 https://memesuite.org/meme/doc/fimo.html
Homer 4.9 http://homer.ucsd.edu/homer/

Original codes used for analysis of ChIP-Seq data are available at GitHub (https://github.com/jjy-lee/ZIC_medulloblastoma/).
