# Supplementary figures and images for: ZIC1 is a context-dependent medulloblastoma driver in the rhombic lip
Source: Nat Genet. 2025 Jan 3;57(1):88–102. doi: 10.1038/s41588-024-02014-z (PMC11735403; doi:10.1038/s41588-024-02014-z)

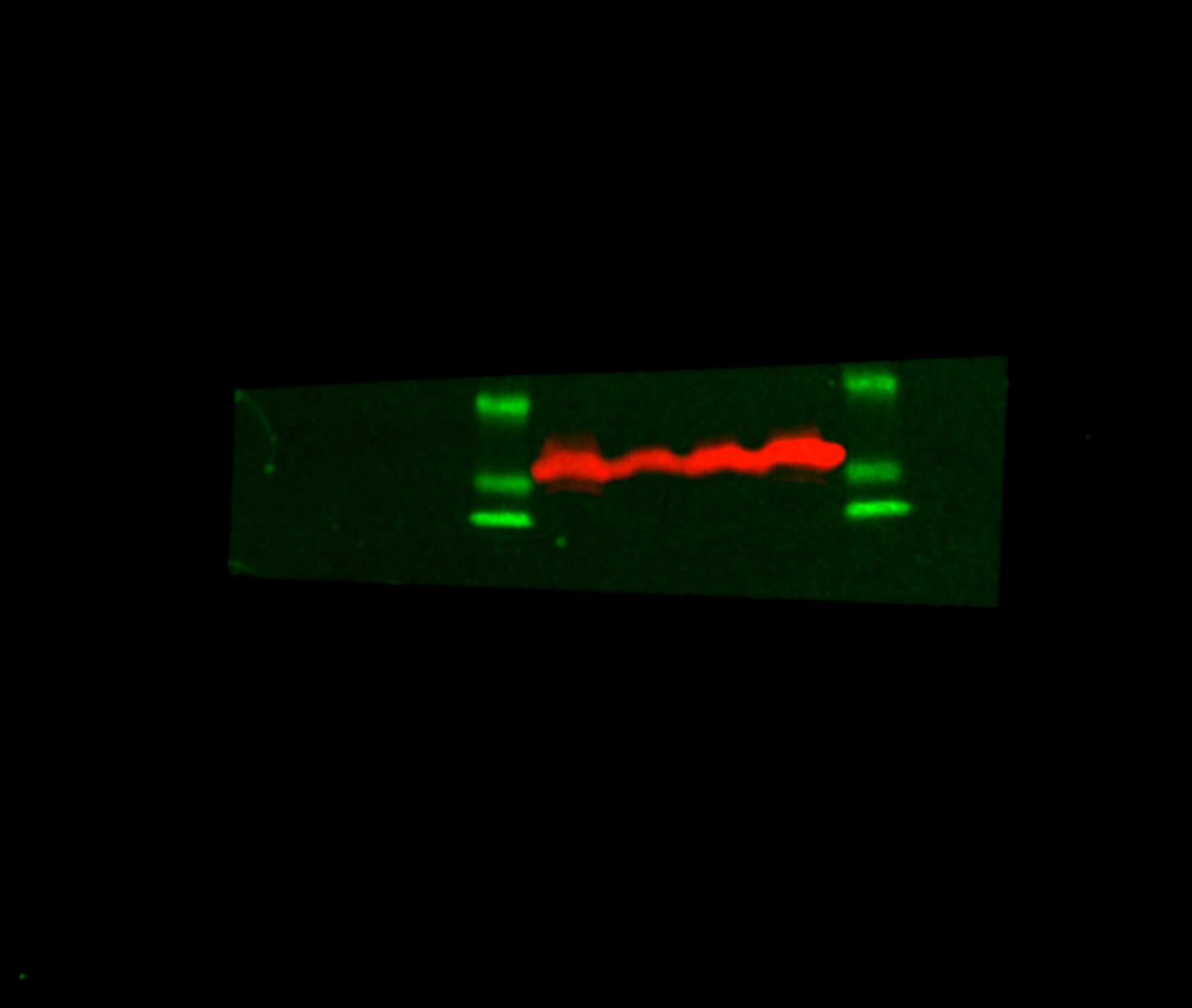

Supplement: Supplementary file 5 — Uncropped and unprocessed gel images. [file 41588_2024_2014_MOESM5_ESM.zip › Fig_6c_D283_ZIC4_H3.tif]

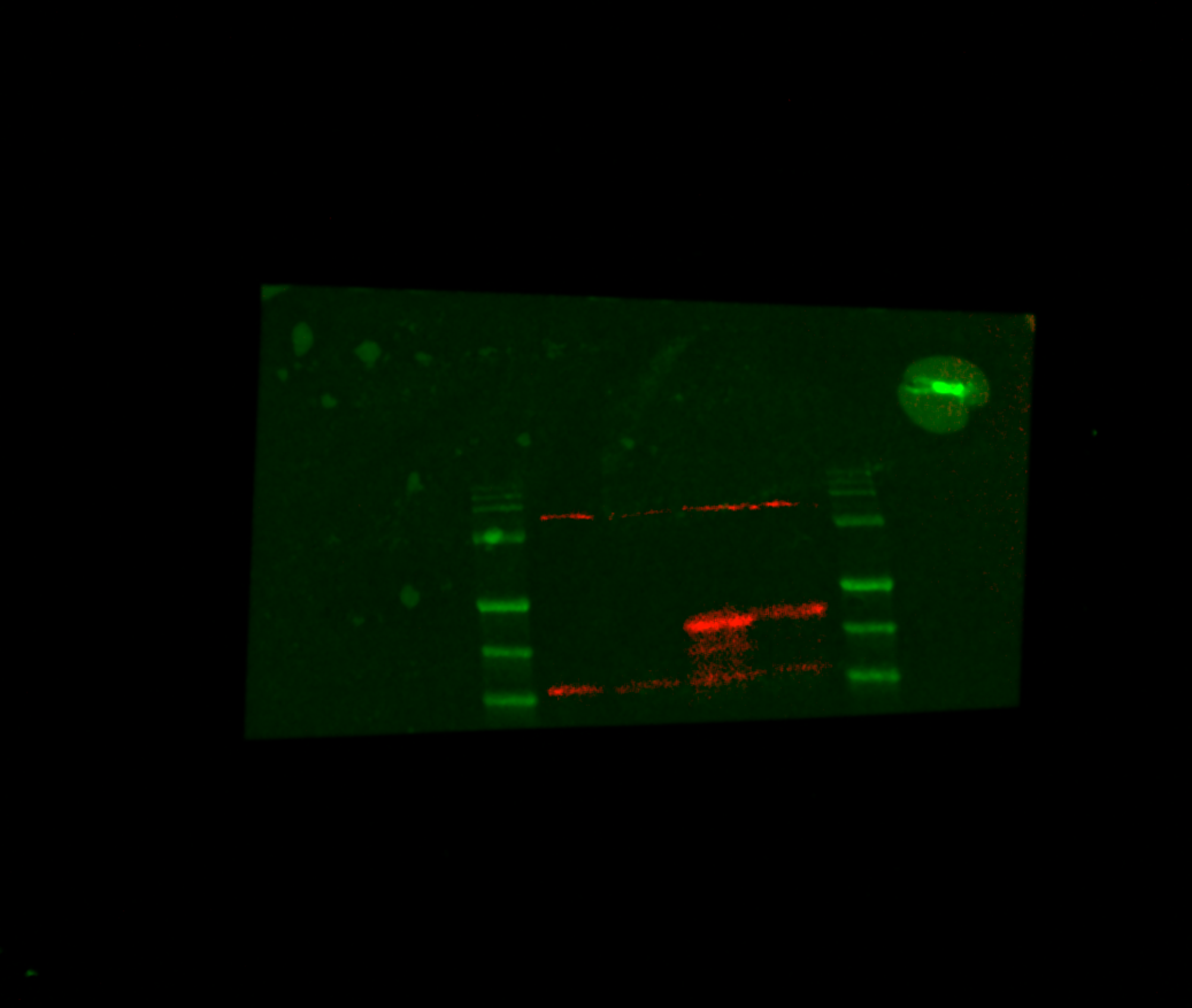

Supplement: Supplementary file 5 — Uncropped and unprocessed gel images. [file 41588_2024_2014_MOESM5_ESM.zip › Fig_6c_D283_ZIC4_ZIC4.tif]

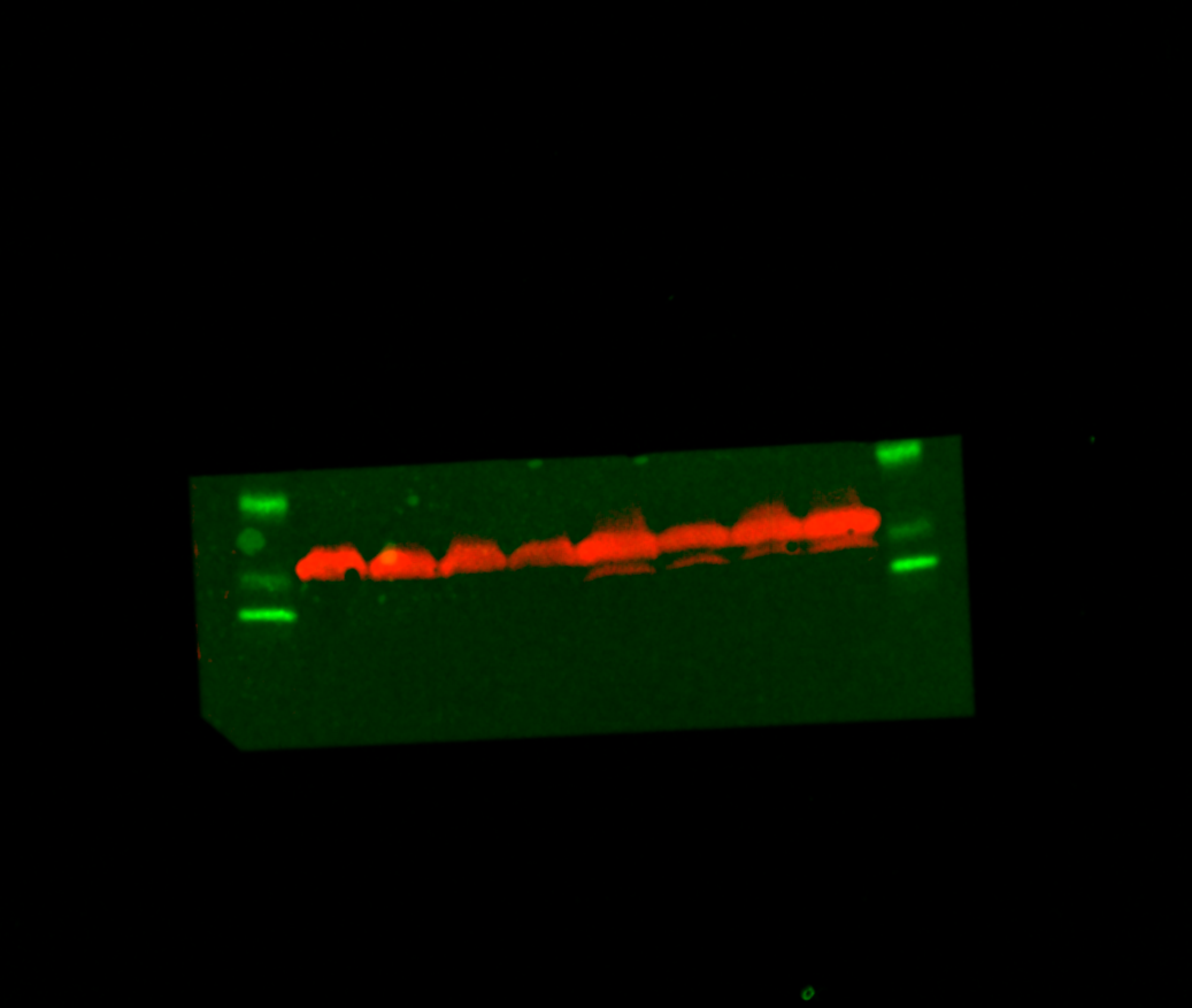

Supplement: Supplementary file 5 — Uncropped and unprocessed gel images. [file 41588_2024_2014_MOESM5_ESM.zip › Fig_6c_D425_D283_ZIC1_H3.tif]

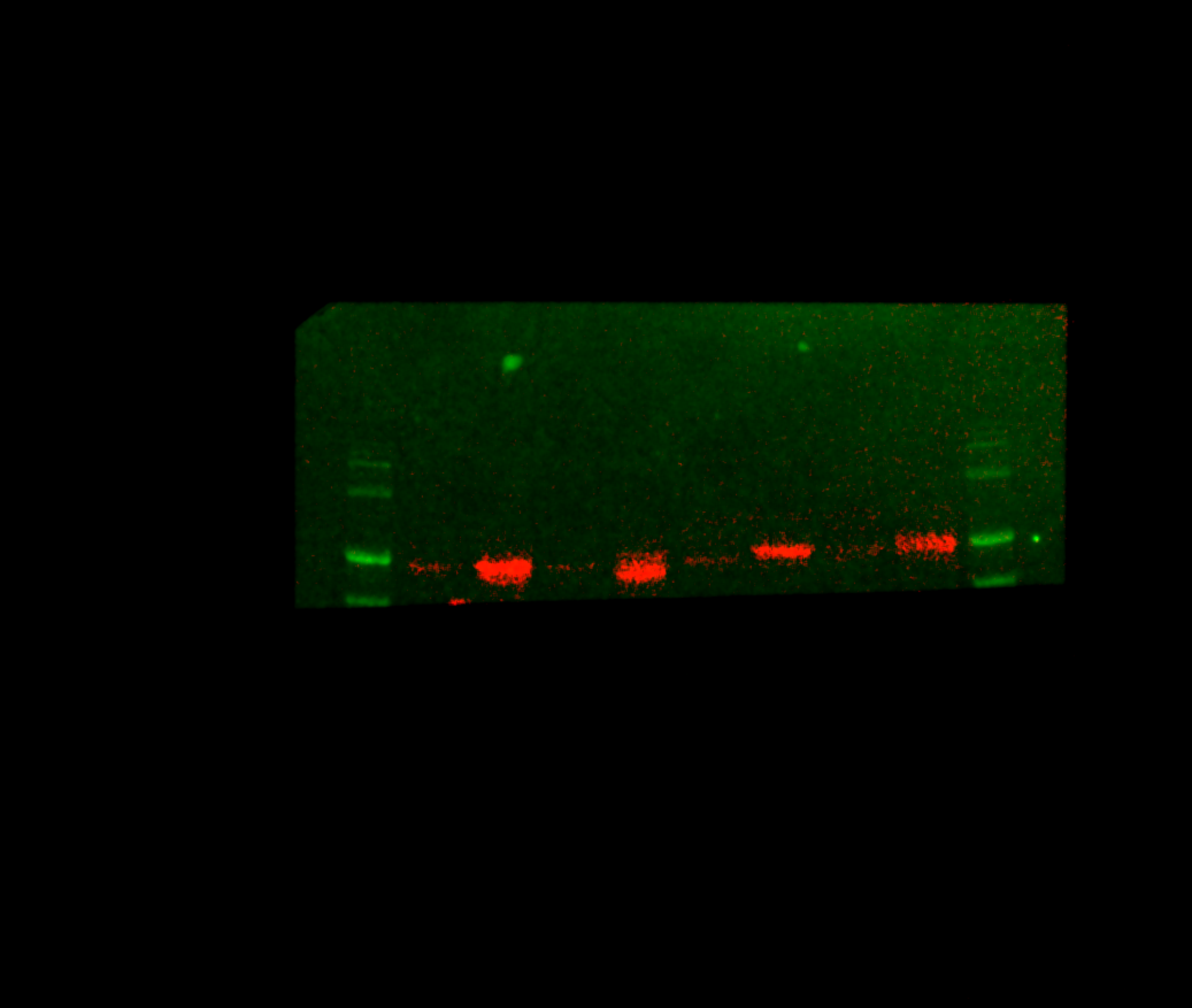

Supplement: Supplementary file 5 — Uncropped and unprocessed gel images. [file 41588_2024_2014_MOESM5_ESM.zip › Fig_6c_D425_D283_ZIC1_ZIC1.tif]

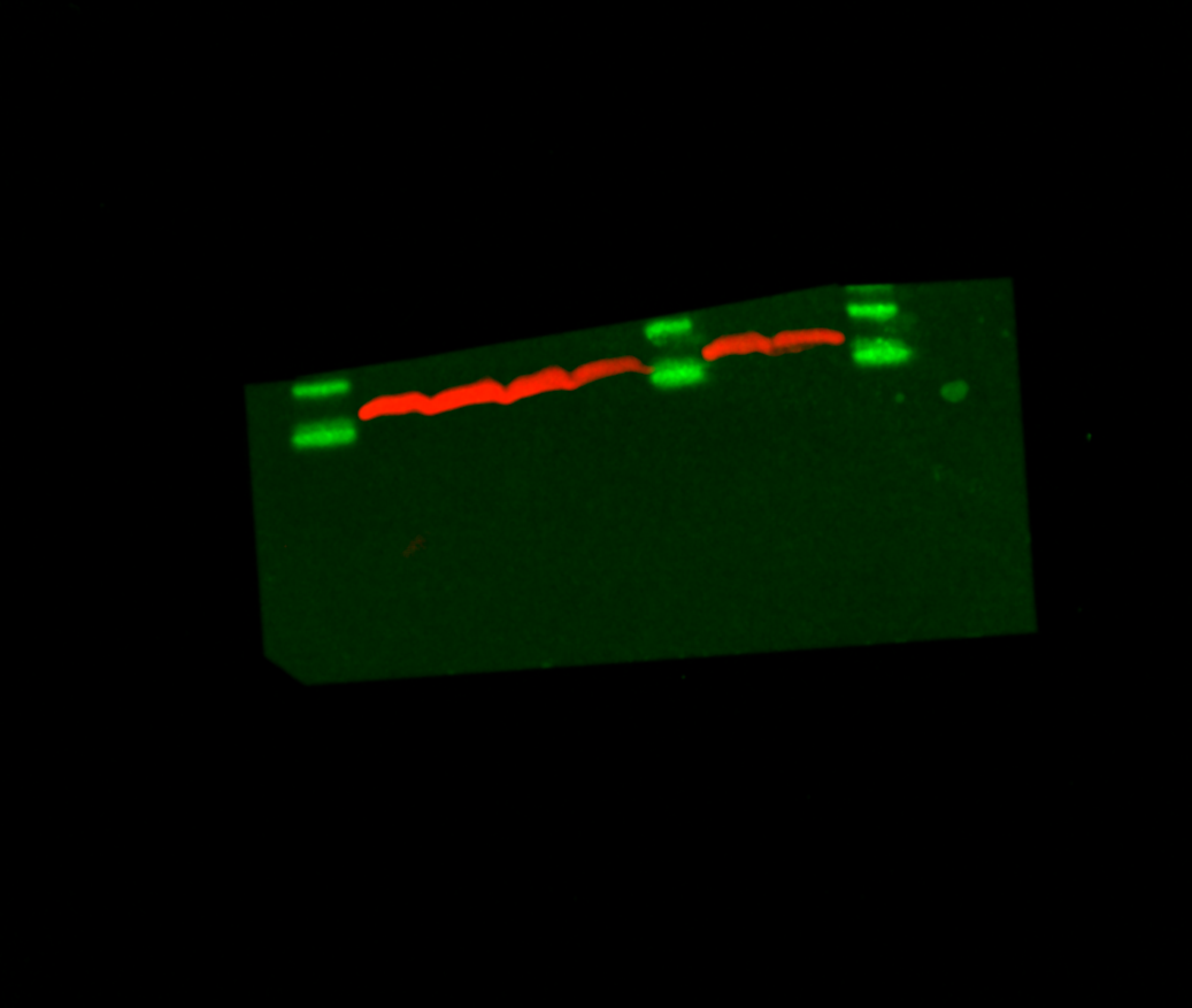

Supplement: Supplementary file 5 — Uncropped and unprocessed gel images. [file 41588_2024_2014_MOESM5_ESM.zip › Fig_6c_D425_ZIC4_H3.tif]

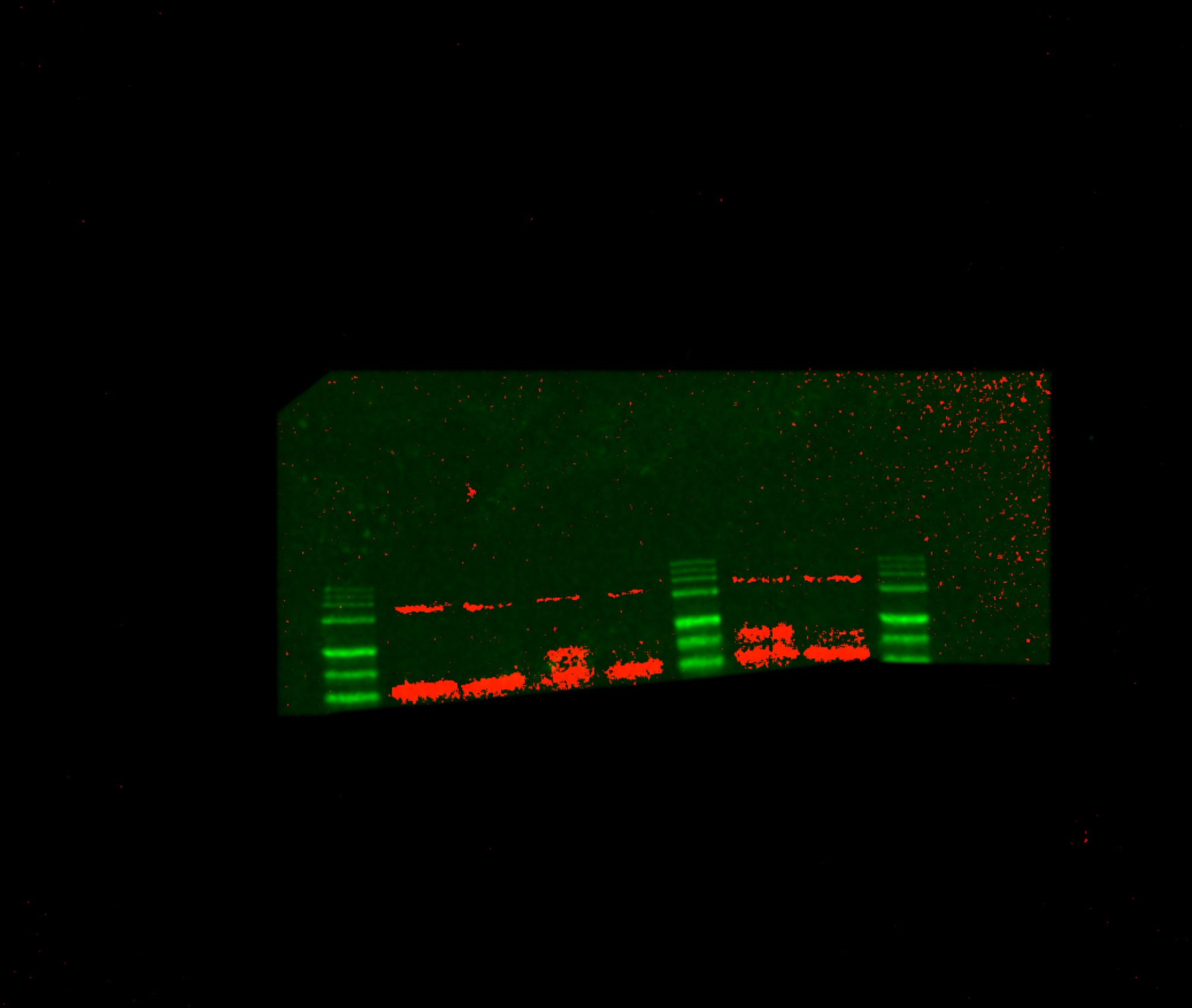

Supplement: Supplementary file 5 — Uncropped and unprocessed gel images. [file 41588_2024_2014_MOESM5_ESM.zip › Fig_6c_D425_ZIC4_ZIC4.tif]

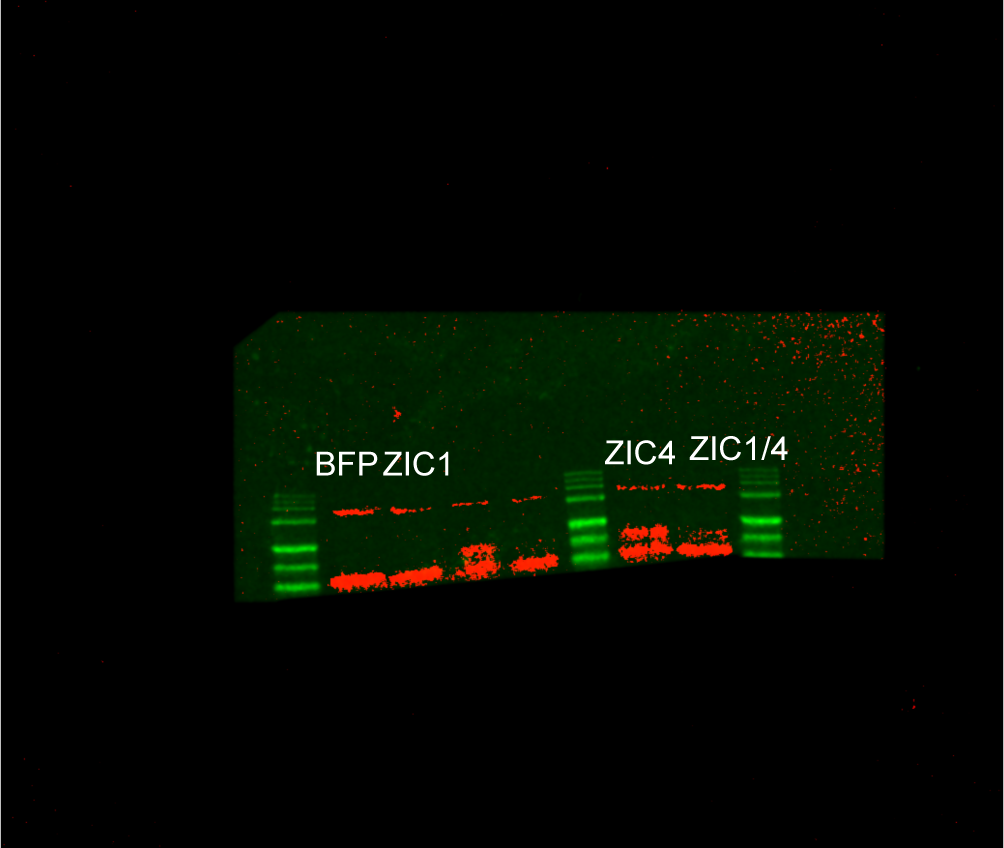

Supplement: Supplementary file 5 — Uncropped and unprocessed gel images. [file 41588_2024_2014_MOESM5_ESM.zip › Fig_6c_D425_ZIC4_ZIC4_labeled.tif]

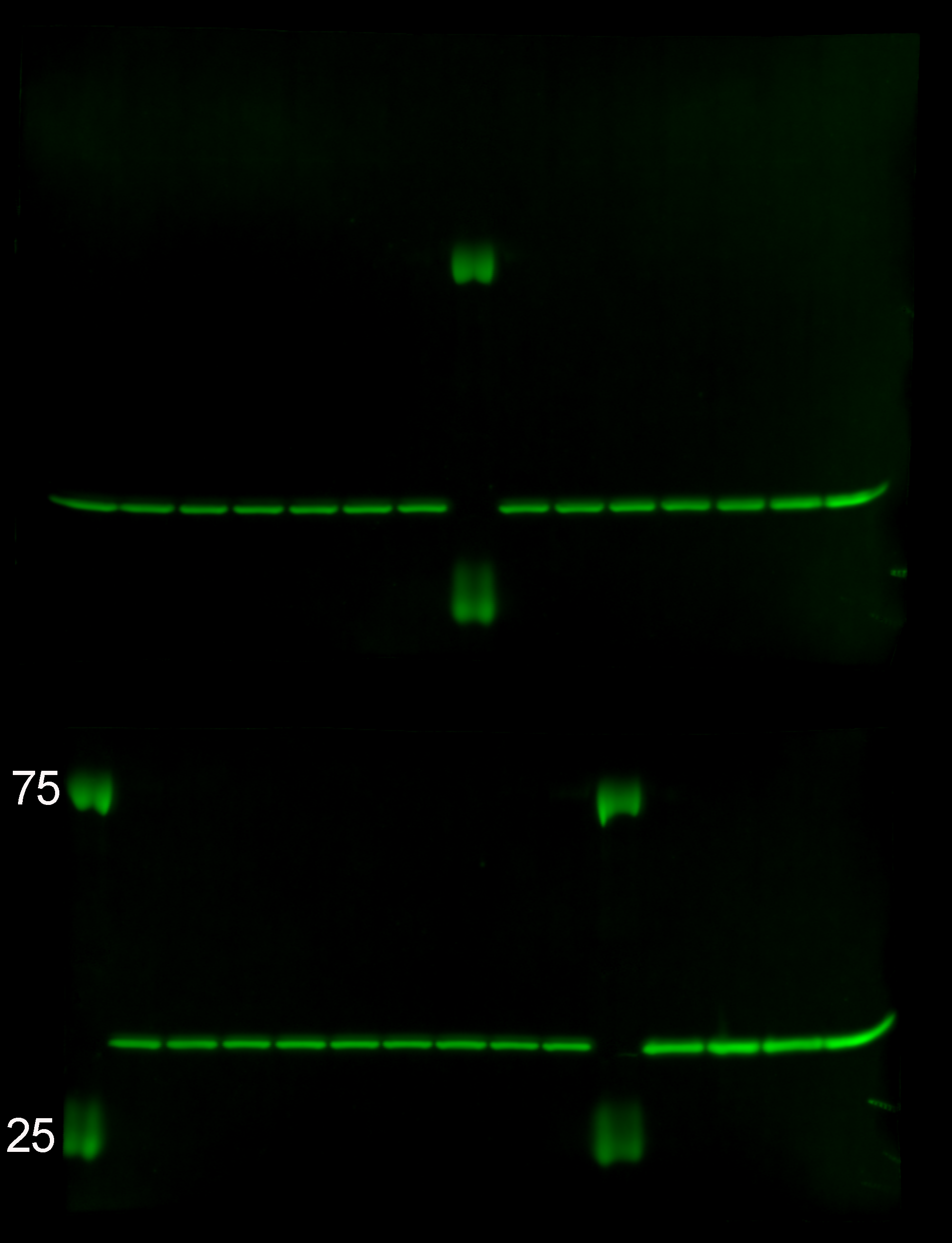

Supplement: Supplementary file 6 — Uncropped and unprocessed gel images. [file 41588_2024_2014_MOESM6_ESM.zip › Fig_7e_D283_zic1-g-GAPDH.tif]

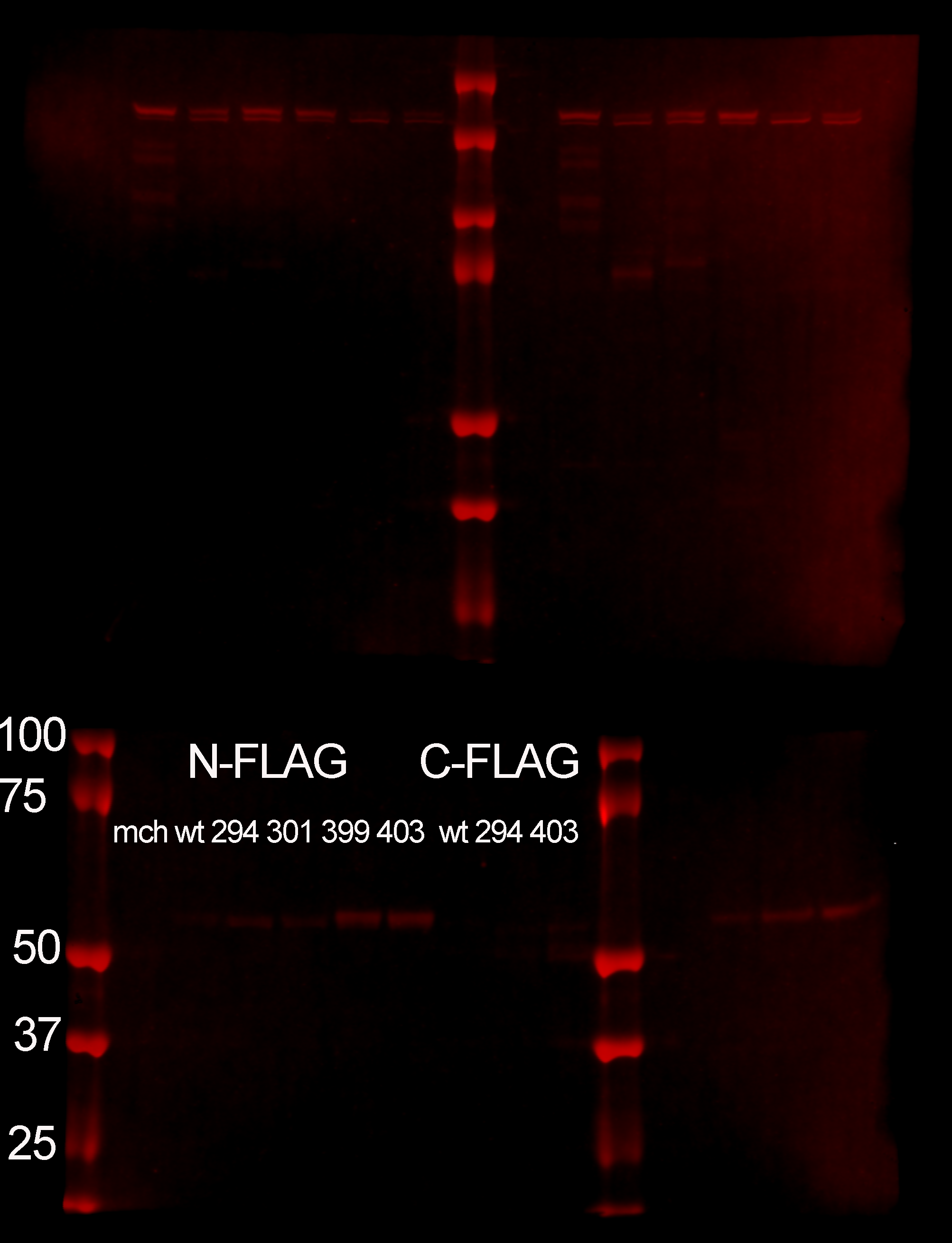

Supplement: Supplementary file 6 — Uncropped and unprocessed gel images. [file 41588_2024_2014_MOESM6_ESM.zip › Fig_7e_D283_zic1-r-FLAG.tif]

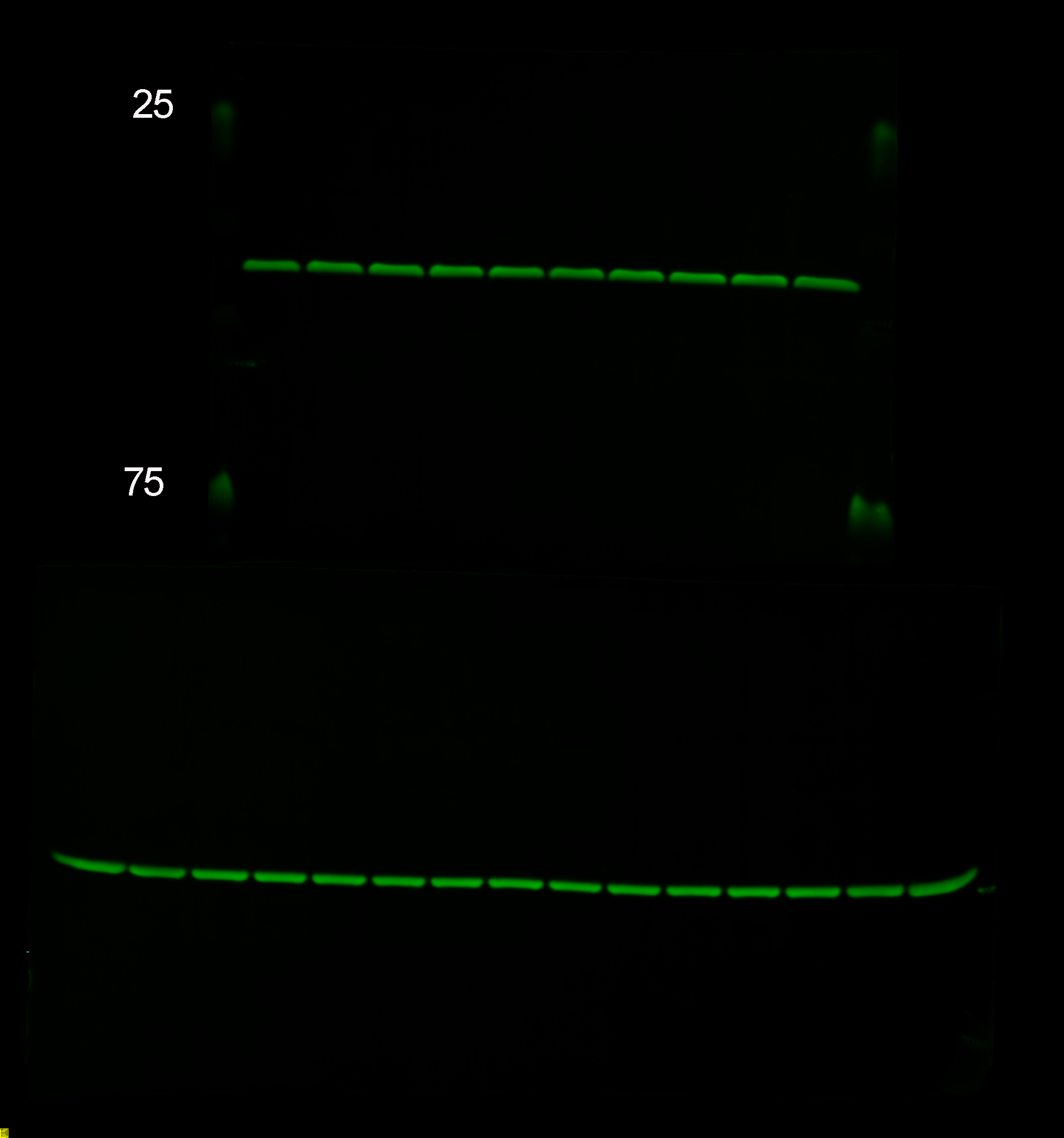

Supplement: Supplementary file 6 — Uncropped and unprocessed gel images. [file 41588_2024_2014_MOESM6_ESM.zip › Fig_7g_D283_zic1_chx_g_actin.tif]

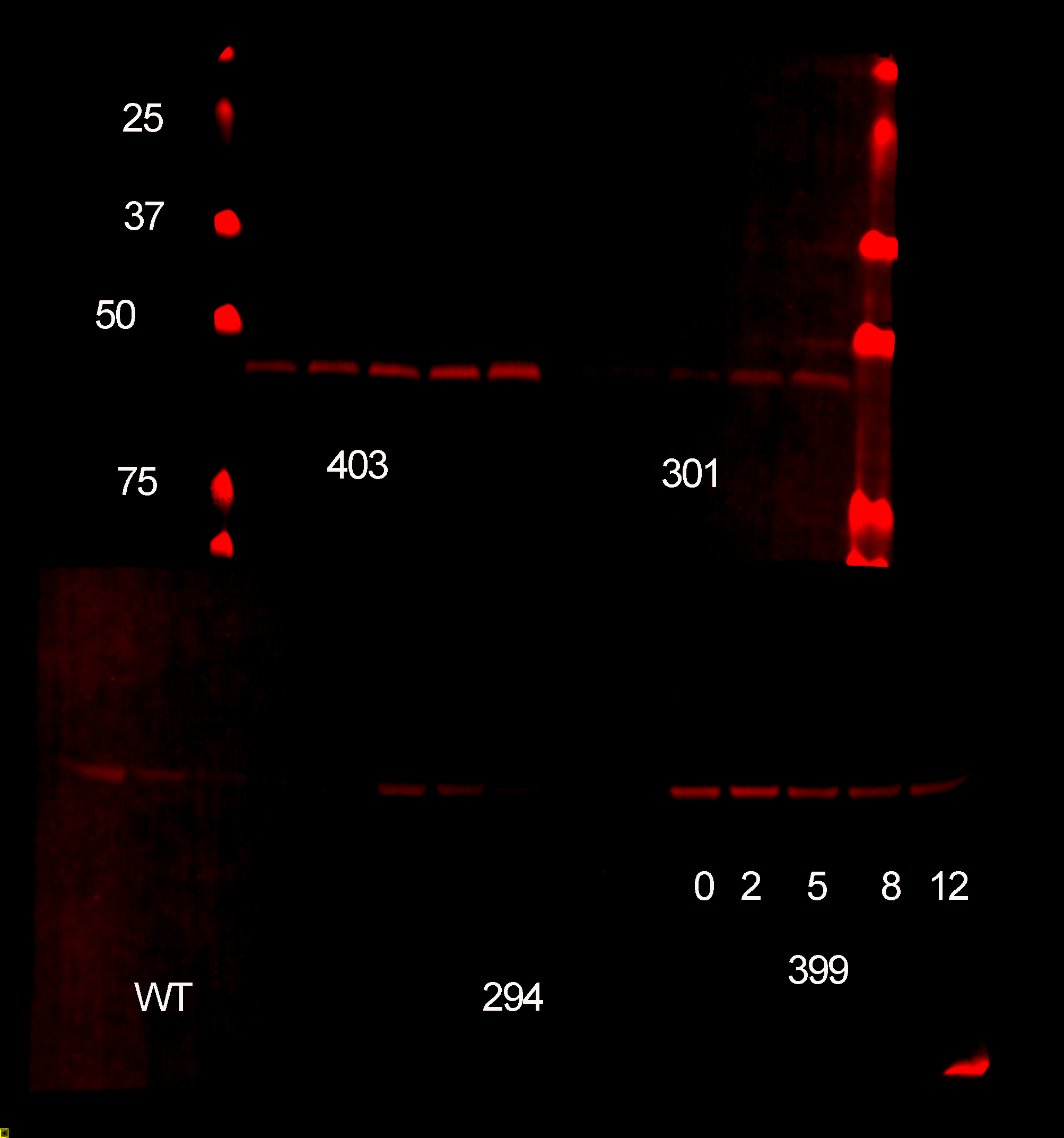

Supplement: Supplementary file 6 — Uncropped and unprocessed gel images. [file 41588_2024_2014_MOESM6_ESM.zip › Fig_7g_D283_zic1_chx_r_FLAG.tif]

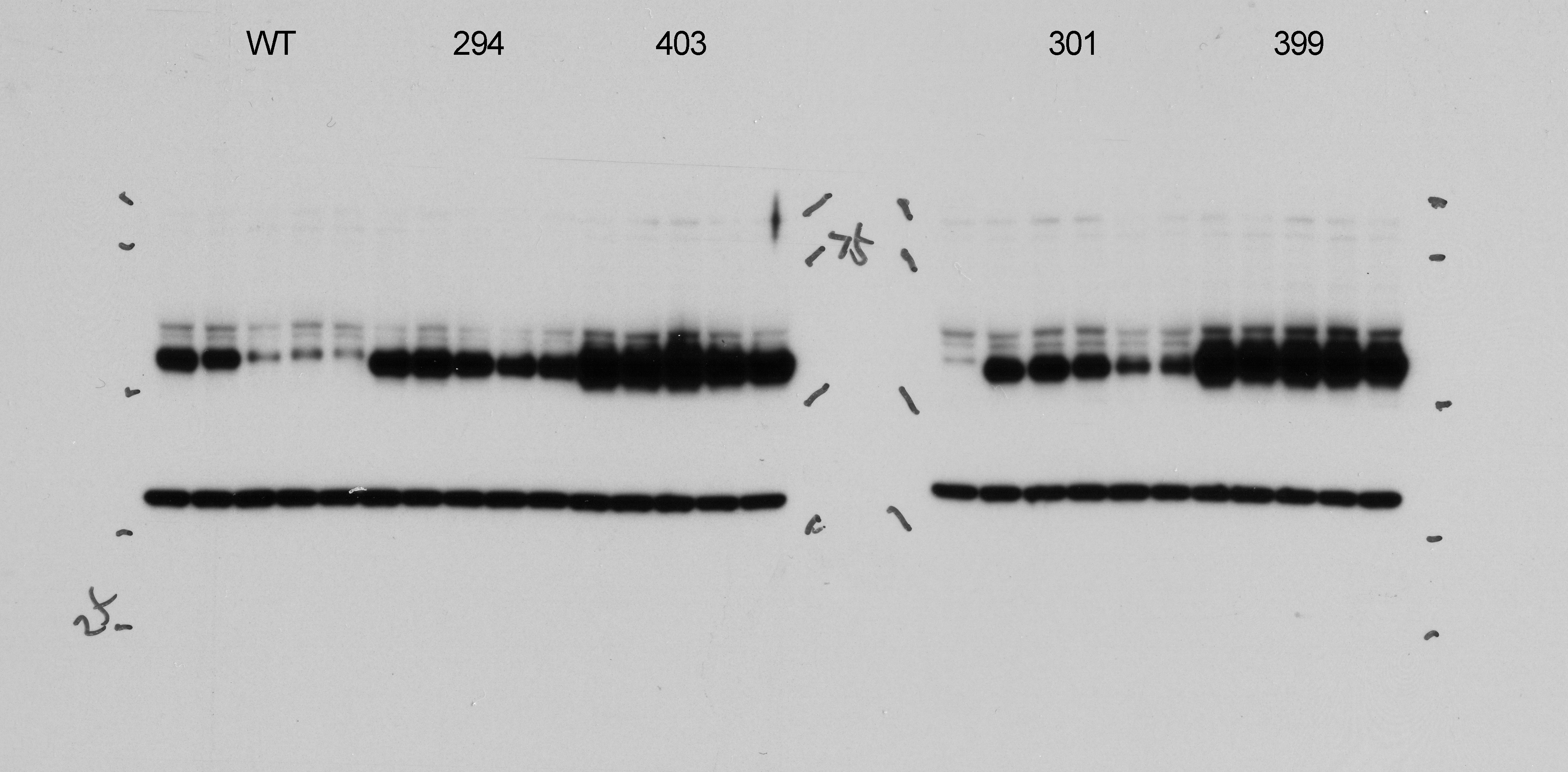

Supplement: Supplementary file 8 — Uncropped and unprocessed gel images. [file 41588_2024_2014_MOESM8_ESM.zip › Fig_8c_GNP_Zic1_CHX.tif]
